# Characterization of secretomes provides evidence for adipose-derived mesenchymal stromal cells subtypes

**DOI:** 10.1186/s13287-015-0209-8

**Published:** 2015-11-11

**Authors:** Natalia Kalinina, Daria Kharlampieva, Marina Loguinova, Ivan Butenko, Olga Pobeguts, Anastasia Efimenko, Luidmila Ageeva, George Sharonov, Dmitry Ischenko, Dmitry Alekseev, Olga Grigorieva, Veronika Sysoeva, Ksenia Rubina, Vassiliy Lazarev, Vadim Govorun

**Affiliations:** Faculty of Medicine, Lomonosov Moscow State University, 31-5, Lomonosovsky av, Moscow, 119191 Russia; Department of Molecular Biology and Genetics, SRI of Physical-Chemical Medicine, 1a, Malaya Pirogovskaya, Moscow, 119435 Russia

**Keywords:** Adipose-derived mesenchymal stromal cells, Secretome profiling, Hypoxic response, Regeneration, Cell therapy

## Abstract

**Introduction:**

This study was aimed at deciphering the secretome of adipose-derived mesenchymal stromal cells (ADSCs) cultured in standard and hypoxic conditions to reveal proteins, which may be responsible for regenerative action of these cells.

**Methods:**

Human ADSCs were isolated from 10 healthy donors and cultured for 3–4 passages. Cells were serum deprived and cell purity was assessed using multiple cell surface markers. Conditioned media was collected and analyzed using LC-MS with a focus on characterizing secreted proteins.

**Results:**

Purity of the ADSC assessed as CD90+/CD73+/CD105+/CD45-/CD31- cells was greater than 99 % and viability was greater than 97 %. More than 600 secreted proteins were detected in conditioned media of ADSCs. Of these 100 proteins were common to all cultures and included key molecules involved in tissue regeneration such as collagens and collagen maturation enzymes, matrix metalloproteases, matricellular proteins, macrophage-colony stimulating factor and pigment epithelium derived factor. Common set of proteins also included molecules, which contribute to regenerative processes but were not previously associated with ADSCs. These included olfactomedin-like 3, follistatin-like 1 and prosaposin. In addition, ADSCs from the different subjects secreted proteins, which were variable between different cultures. These included proteins with neurotrophic activities, which were not previously associated with ADSCs, such as mesencephalic astrocyte-derived neurotrophic factor, meteorin and neuron derived neurotrophic factor. Hypoxia resulted in secretion of 6 proteins, the most prominent included EGF-like repeats and discoidin I-like domains 3, adrenomedullin and ribonuclease 4 of RNase A family. It also caused the disappearance of 8 proteins, including regulator of osteogenic differentiation cartilage-associated protein.

**Conclusions:**

Human ADSCs with CD90+/CD73+/CD105+/CD45-/CD31-/PDGFRβ+/NG2+/CD146+(−) immunophenotype secrete a large array of proteins, the most represented group is comprised of extracellular matrix components. Number of secreted proteins is largely unaffected by prolonged hypoxia. Variability in the secretion of several proteins from cultured ADSCs of individual subjects suggests that these cells exist as a heterogeneous population containing functionally distinct subtypes, which differ in numbers between donors.

**Electronic supplementary material:**

The online version of this article (doi:10.1186/s13287-015-0209-8) contains supplementary material, which is available to authorized users.

## Introduction

Multipotent mesenchymal stromal cells, isolated from bone marrow or adipose tissue (ADSC), enhance tissue regeneration upon transplantation [1], by guiding the amplification and differentiation of resident stem cells as well as by stimulating the growth of blood vessels and nerves [2–4]. Recent studies indicated that ADSCs effects on regeneration are mostly mediated by their ability to produce a wide range of bioactive molecules (growth factors, cytokines, etc.) [5, 6] as well as extracellular vesicles [7]. Therefore, the secretome of cultured ADSCs was suggested as an alternative for cell therapy and many efforts to decipher its contents were made using various approaches (reviewed in [8]). Several candidate factors, which mediate the beneficial effects of the ADSCs secretome on tissue regeneration, were identified, including vascular endothelial growth factor (VEGF), hepatocyte growth factor (HGF), insulin-like growth factor ( IGF-1), platelet-derived growth factor (PDGF-BB), angiopoietin-like 4 protein, and brain-derived neurotrophic factor (BDNF) [9, 10]. However, the content of factors necessary for the stimulation of tissue regeneration by ADSCs remains only partially characterized. The efficiency of ADSC-based therapies varies between different donors, but the results of the experimental and clinical studies analyzing the impact of donor-specific factors, including age, sex and concomitant disorders, on the efficiency of cell therapy were controversial [11–14]. Molecular mechanisms underlying donor-dependent variations of ADSC activities remain to be elucidated as well as a set of biomarkers, which would allow predicting ADSCs regenerative activity in vivo.

Several authors including us have used hypoxia as a tool to further enhance the regenerative potential of ADSCs, because hypoxic treatment caused coordinated changes of expression of genes involved in the stimulation of regeneration [15–17]. We analyzed secretomes of ADSCs derived from ten healthy female donors of similar age cultured in standard (21 % O_2_) or hypoxic (1 % O_2_) conditions. More than 600 secreted proteins were detected in conditioned media of ADSCs, many of which may promote tissue regeneration; their number is largely unaffected by prolonged hypoxia. Despite an identical immunophenotype, growth characteristics and differentiation abilities, only 100 proteins were common to all cultures. In addition, ADSCs from the different subjects secreted proteins which were variable between different cultures, including ones responsible for tissue regeneration. Variability in the secretion of several proteins by ADSCs of individual subjects suggests that these cells exist as a heterogeneous population containing functionally distinct subtypes which differ in numbers between patients.

## Methods

### ADSCs culture and conditioned medium harvesting

Human ADSCs were isolated from subcutaneous adipose tissue obtained from ten female donors during abdominal surgery [1]. All donors gave their informed consent and the local ethics committee of city clinical hospital #31 (Moscow, Russia) approved the study protocol. All donors were < 50 years old and did not have obesity or acute inflammation (Additional file 1: Table S1). All ADSC cultures were isolated from the same fat depot. This significantly limited the size of the initial sample. Therefore, we had to culture cells up to the third or fourth passages to collect a sufficient amount of material for analysis. Cells were cultured in AdvanceSTEM Mesenchymal Stem Cell Media containing 10 % AdvanceSTEM Supplement (HyClone, South Logan, Utah, USA), 1 % antibiotic–antimycotic solution (HyClone) at 37 °C in 5 % CO2 incubator. Cells were passaged at 70 % confluency using HyQTase solution (HyClone). For the experiments, ADSCs of the third or fourth passages were seeded at a density of 7x10^3^ cells/cm^2^ on uncoated culture plates (Corning, NY, USA) a day before the experiment. ADSCs were washed thoroughly five times with HBSS without Ca^2+^ and Mg^2+^ supplemented with x100 MEM amino acid (Gibco, Waltham, MA USA) on a rotary platform placed in a CO_2_ incubator to remove any residues of AdvanceSTEM Supplement. After the last wash, HBSS was replaced by serum-free AdvanceSTEM Mesenchymal Stem Cell Media supplemented with x100 MEM amino acid (Gibco). For assessment of secretome content ADSCs were cultured either under standard conditions (5 % CO_2_ and 21 % O_2_) or under hypoxia (5 % CO_2_ and 1 % O_2_) at 37 °C for 48 h [18]. At the end of the incubation, conditioned medium was harvested and cultured cells were subjected to an analysis of immunophenotype and viability. The volume of harvested medium was measured and 100 mM PMSF solution and 200 mM EDTA solution were added to 2 and 5 mM concentration, respectively. Cell debris was removed by centrifugation for 15 min at 200 g. The resulting supernatant was filtered through a 0.45 μm filter (BD Falcon, San Jose, CA, USA) and snap-frozen in liquid N_2_. The quantity and viability of the ADCSs were assessed at the end of the experiment using a Countess cell counter (Invitrogen, Waltham, MA, USA).

### ADSCs immunophenotyping

The limited size of starting material did not allow us to perform immunophenotype analysis before the initial plating of cells. To confirm that ADSCs are multipotent mesenchymal stromal cells we analyzed their immunophenotype according to published criteria [19]. For phenotypic analysis cells were stained with four different antibody combinations: (1) CD45/IgG-PE/IgG-PerCP-Cy5.5/IgG-PC5; (2) CD45/СD7-PE/IgG-PerCP-Cy5.5; (3) CD45/CD73-PE/CD105-PerCP-Cy5.5; and (4) CD45/IgG-PE/CD90-PC5. For patients 7–10 CD90-PC5 was replaced with CD90-PE and IgG-PC5 was removed. Cells from patients 2–9 were additionally stained with antibodies against neuro-glial proteoglycan 2 (NG2-FITC, Chemicon), platelet-derived growth factor receptor B (PDGFRB-PE, BD Pharmingen), CD31-Alexa647 (BD Pharmingen, San Jose, CA, USA), CD34-PerCP (BD Pharmingen), and CD146-PE (BD Pharmingen). After 1 h staining at +4 °C cells were washed once with PBS and analyzed live within 1 h using a LSR Fortessa flow cytometer (BD Biosciences) and FACSDiva software (BD). PerCP-Cy5.5 and PC5 were analyzed with 640 nm red laser excitation that directly excite Cy5.5 or Cy5 dye and 710–750 nm emission filter.

### ADSCs differentiation assays

ADSCs abilities to differentiate into osteogenic and adipogenic directions were tested in vitro using standard differentiation and analysis protocols [11] in a normoxic environment. Briefly, osteogenic differentiation was induced by plating 6×10^4^ of ADSCs onto a 24-well plate and incubating in AdvanceSTEM Mesenchymal Stem Cell Media containing 10 % AdvanceSTEM Supplement (HyClone), 1 % antibiotic–antimycotic solution (HyClone), containing 10^−8^ М dexamethasone, 10 mМ β-glycerol-2-phosphate, 0.2 mМ 2-phospho-L-ascorbic acid (Invitrogen, USA) for 21 days. Differentiation efficiency was analyzed using Alizarin Red S staining for calcium accumulation. Adipogenic differentiation was induced by three cycles of consecutive incubation for six days in growth medium containing 10^−6^ М dexamethasone, 10 μM insulin, 200 μM indomethacin and 0.5 mM 3-isobutyl-1-methylxanthine (Invitrogen, USA) followed by three days incubation in growth medium containing 10 μM insulin. Cells accumulated intracellular lipids which were analyzed using Oil-Red-O staining.

### Gel-free digestion of the protein sample with DTT

The pellet was resuspended in 50 μl 100 mM ammonium bicarbonate with 10 mM DTT (BioRad, Hercules, California, USA) and 1 μl protease inhibitor Mix (GE Healthcare, Pittsburgh, PA, USA), mixed in a vortex, and heated at 100 °C for five min. After cooling to room temperature, the insoluble material was removed by centrifugation at 15,000 g for five min. Supernatant was separated and protein concentration was determined by Bradford (Bradford Protein Assay Kit, BioRad). In the supernatant, reduced protein disulfide bonds were alkylated with 30 mM iodoacetamide (BioRad) in 100 mM ammonium bicarbonate at room temperature and in the dark for 30 min and 10 mM DTT (BioRad) in 100 mM ammonium bicarbonate was added iteratively. Upon alkylated trypsin (Trypsin Gold, Mass Spectrometry Grade, Promega, Madison, WI, USA) in ratio trypsin:protein equal 1:30 was added to the supernatant and incubated at 37 °C overnight. For inactivation of trypsin activity a stock solution of trifluoroacetic acid (Sigma, St. Louis, MO, USA) (final concentration in sample 0.5 %) was added to the digest. Small amounts of the digest for liquid chromatography/mass spectrometry (LC-MS) analysis were desalted on ZipTip (Millipore, Billerica, MA, USA).

### Liquid chromatography/mass spectrometry analysis and protein identification

Analysis was performed on a TripleTOF 5600+ mass-spectrometer with a NanoSpray III ion source (ABSciex, Concord, Ontario, Canada) coupled to a NanoLC Ultra 2D+ nano-HPLC system (Eksigent, Concord, Ontario, Canada). The high-performance liquid chromatography (HPLC) system was configured in a trap-elute mode. For a sample loading buffer and buffer A, the mix of 98.9 % water, 1 % methanol, 0.1 % formic acid (v/v) was used. Buffer B was 99.9 % acetonitrile, 0.1 % formic acid (v/v). The trap column was conditioned prior to use by the same solvent as the column itself (95 % of solution A (H20 + 1 % MeOH + 0.1 % formic acid) and 5 % of solution B (AcN + 0.1 % formic acid) during 25 min with a flow rate of 300 nl/min.

Samples were loaded on a trap column Chrom XP C18 3 microm 120 Å 350 microm*0.5 mm (Eksigent, Dublin, CA, USA) at a flow rate of 3 ul/min over 10 min and eluted through the separation column 3C18-CL-120 (3 microm 120 Å) 75 microm*150 mm (Eksigent) at a flow rate of 300 nl/min. The gradient was from 5 to 40 % of buffer B in 120 min. The column and the precolumn were regenerated between runs by washing with 95 % of buffer B for seven min and equilibrated with 5 % of buffer B for 25 min. To ensure the absence of carryover both the column and the precolumn were thoroughly washed with a blank trap-elute gradient that included five seven-min 5-95-95-5 %B waves followed by 25 min 5 % buffer B equilibration between different samples.

Mass spectra were acquired in a positive ion mode. The information-dependent mass-spectrometer experiment included one survey MS1 scan followed by 50 dependent MS2 scans. MS1 acquisition parameters were as follows: mass range for analysis and subsequent ion selection for MS2 analysis was 300–1250 m/z, signal accumulation time was 250 ms. Ions for MS2 analysis were selected on the basis of intensity with the threshold of 250 cps and the charge state from 2 to 5. MS2 acquisition parameters were as follows: resolution of quadrupole was set to UNIT (0.7 Da), measurement mass range was 200–1800 m/z, optimization of ion beam focus was to obtain maximal sensitivity, signal accumulation time was 50 ms for each parent ion. Collision activated dissociation was performed with nitrogen gas with collision energy ramping from 25 to 55 V within 50 ms signal accumulation time. Analyzed parent ions were sent to a dynamic exclusion list for 15 s in order to get an MS2 spectra at the chromatographic peak apex (minimum peak width throughout the gradient was about 30 s). Instrument reproducibility is controlled during routine process and technical duplicates were not run. Temporal biases were not reduced.

For protein identification, .wiff data files were analyzed with ProteinPilot 4.5 revision 1656 (ABSciex) using the search algorithm Paragon 4.5.0.0 revision 1654 (ABSciex) and a standard set of identification settings to search against Uniprot Swissprot (dated 20131002) database. The following parameters were used: alkylation of cysteine - iodoacetamide, trypsin digestion, TripleTOF 5600 equipment, and species: Homo sapiens, thorough search with additional statistical FDR analysis. Peptide identifications were processed with default settings by a ProteinPilot software built-in ProGroup algorithm. Our approach allows us to detect 90 % of peptides with a concentration > 5 fMol and 1,500 of the most presented proteins. The final protein identification list was obtained with the threshold reliable protein ID unused score calculated by ProteomicS Performance Evaluation Pipeline Software (PSPEP) algorithm for 1 % global FDR from fit and with the requirement for more than two unique peptides for each protein.

### RNA isolation and transcriptome analysis

To confirm the expression of identified proteins, we performed gene array experiments. Total cellular RNA was isolated from normoxic ADSCs using a RNeasy Kit (Qiagen, Venlo, The Netherlands, cat # 74104) according to the manufacturer’s instructions. Five hundred nanograms of total RNA was labeled and hybridized on HumanHT-12 v4 Expression BeadChip (Cat. no. BD-103-0204; Illumina, San Diego, CA, USA), according to the manufacturer’s recommendations (Illumina Gene Expression Profiling Assay Guide). BeadChips were scanned with the Illumina iScan Reader. Data were imported into GenomeStudio (Illumina) and analyzed using built-in modules. Signals with detection *p* value <0.05 were considered as significant.

### Real-time PCR

To confirm changes of protein content under hypoxic treatment, we performed real-time PCR using total RNA isolated from normoxic and hypoxic ADSCs. cDNA was synthesized using Fermentas Reverse Transcription Reagents (Fermentas, Vilnius, Lithuania) with oligo-dT and RevertAid™ M-MuLV Reverse Transcriptase (Fermentas) according to the manufacturer’s instructions. Real-time PCR was performed using ready-to-use reaction mix, containing DNA polymerase, SYBR Green and ROX (Evrogen, Moscow, Russia) in 7500 Fast Real-time PCR system (Applied Biosystems, South Logan, Utah, USA). The following oligonucleotide primers were used for amplification:

VEGFA: forward CAACATCACCATGCAGATTATGC, reverse GCTTTCGTTTTTGCCCCTTTC; EDIL: forward AAATGGAGGTATCTGTTTGCCAG, reverse CCCCTCGGTATGCTTCACTTATT; RNASE4: forward TGCAGAGGACCCATTCATTGC, reverse TCAAGTTGCAGTAGCGATCAC; ADML: forward TGCCCAGACCCTTATTCGG, reverse AGTTGTTCATGCTCTGGCGG; CRTAP: forward GAAGCATCCTGATGACGAAATGA, reverse AGTTCTCACCGTTGTATGCCC; HSP90AB2P: forward AGTTGGACAGTGGTAAAGAGCT, reverse TCCACTACTTCTTTGACCTGCA; GCSF: forward CCCTCCCCATCCCATGTATTTATC, reverse ACCTATCTACCTCCCAGTCCAG; EEF1A1: forward TGTCGTCATTGGACACGTAGA, reverse ACGCTCAGCTTTCAGTTTATCC.

Fold change of mRNA expression in hypoxic samples was calculated using the 2^-ΔΔCt^ method, EEF1A1 was used as a reference gene.

### Protein electrophoresis and Western blotting

To confirm ADSC response to hypoxia, HIF-1 alpha content was analyzed using Western blotting. Protein electrophoresis was carried out under denaturing conditions with sodium dodecyl sulfate according to Laemmli [20]. Cells lysed in buffer with 1 % Triton X-100 were separated in 10 % 1 mm PAAG (30 μg of protein per lane) at 120 V before the tracking dye release. Protein molecular weight was estimated using a pre-stained protein ladder (BioRad). Separated proteins were transferred to a PVDF membrane (Millipore) by semi-dry electroblotting [21] at 25 V for 45 min in buffer for protein transfer. The membrane with transferred protein was incubated in phosphate buffer (PBS) with 5 % fat-free milk and 0.01 % Tween-20 for 1 h. The membrane was incubated with primary mouse monoclonal antibodies to HIF-1 alpha (Abcam, Cambridge, UK) overnight, followed by four washes in PBS with 0.01 % Tween-20. Then membranes were incubated with secondary anti-mouse antibodies conjugated with horseradish peroxidase (R&D) and washed with PBS with 0.01 % Tween-20. Protein bands were visualized with BioMax roentgen film (Kodak, Rochester, NY, USA) by a chemiluminescence technique. Luminescence was initiated by luminol reaction with hydrogen peroxide (ECL, Amersham, Pittsburgh, PA, USA) catalyzed by horseradish peroxidase conjugated with secondary antibodies. Protein amounts in samples were normalized by GAPDH protein content.

### Enzyme-linked immunosorbent assay

ADSC secretomes were analyzed for accumulation of granulocyte-colony stimulating factor (G-CSF) using Quantikine enzyme-linked immunosorbent assay (ELISA) (#DCS50, R&D Systems, Minneapolis, MN, USA) according to the manufacturer’s instructions. Concentration of G-CSF in individual samples was normalized to total protein concentration measured by Bradford assay.

### Statistics and bioinformatics

Identified proteins were analyzed for the possibility of secretion using SignalP (http://www.cbs.dtu.dk/services/SignalP), SecretomeP (http://www.cbs.dtu.dk/services/SecretomeP) and ExoCarta (http://www.exocarta.org) databases and further subjected to bioinformatic analysis. To determine over-represented proteins for both hypoxia and control samples we used a hypergeometric test (confidence level P-value < = 0.05). Functional annotation clustering was performed using DAVID Bioinformatics Resources 6.7 (https://david.ncifcrf.gov), using default settings. Functional clusters with *p*-value <0.05 were considered strongly enriched in the annotation categories [22]. Statistical analysis of real-time PCR and ELISA data was performed using Statistica 8.0 software. Values are expressed as mean ± standard error of the mean (SEM). Data were assessed for normality of distribution using the Kolmogorov-Smirnov test. Differences between treatment and control groups were then analyzed using Student’s *t*-test. Statistical significance was defined as *p*-value <0.05.

## Results

### Characterization of ADSCs secretomes

**Fig. 1:**
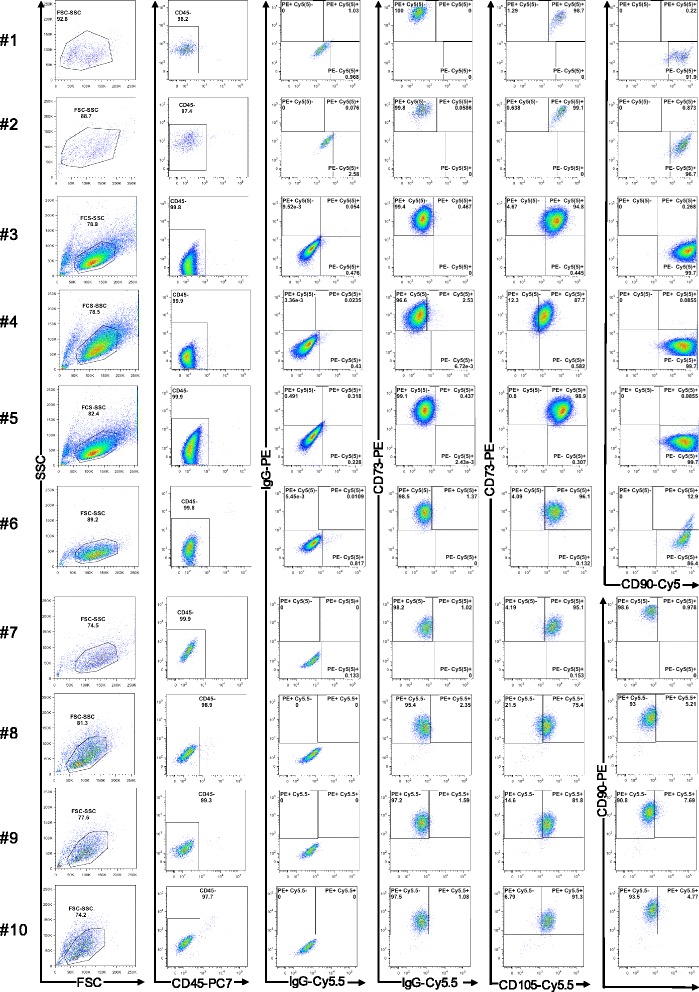
Representative flow cytometry plots of MSC surface markers expression on ADSC obtained from ten different donors. Plots from *left* to *right* in each *row*: forward and side scattered plot of analyzed population; CD45 expression; control IgGs staining; CD73 expression; CD73 and CD105 expression; CD90 expression. *MSC* mesenchymal stromal cells, *ADSC* adipose-derived mesenchymal stromal cells

ADSCs of third or fourth passages cultured in serum-free medium for 48 h demonstrated more than 97 % viability at the end of the incubation, and > 99 % of these cells possessed CD105^+^CD73^+^CD90^+^CD45^−^ immunophenotype (Fig. 1) suggested for multipotent mesenchymal stromal cells [19]. They were capable of osteogenic and adipogenic differentiation (Additional file 2: Figure S1). More than 90 % of these cells expressed the perycite markers PDGFRβ and NG2 proteoglycan. In contrast to these proteins, the expression of CD146 antigen varied between ADSCs populations from different donors, 22 ± 14.5 % of cells were positively stained with specific antibody (Additional file 3: Figure S2). Using LC-MS, 606 secretory proteins were identified in ADSCs conditioned medium (for protein catalogue see Additional file 1: Table S2). Of these, 452 proteins were considered as secreted through a classical pathway (endoplasmic reticulum/Golgi apparatus-dependent pathway), because a signal peptide was predicted by the Signal Peptide Database, while 157 proteins were secreted through nonclassical pathways. The expression of mRNAs corresponding to the identified proteins was confirmed using Illumina Gene Expression Profiling Assay (Additional file 4: Table S3).

Despite similar anthropometric characteristics of donors included in the study, the same culture conditions, growth characteristics, immunophenotype and differentiation potential of ADSCs, only 100 proteins were common to all cultures (Additional file 1: Table S4). Functional annotation clustering has revealed 72 clusters. Those with the highest enrichment score and protein counts included extracellular matrix (ECM) proteins, proteins involved in cell adhesion, and matrix proteases as well as their inhibitors (Table 1 and Additional file 1: Table S4). Proteins common to all ADSC cultures also included molecules involved in blood vessel development, wound healing, immune response, and neuron projection development and regeneration, such as M-CSF, PEDF, DKK3, olfactomedin-like 3, follistatin-like 1, and prosaposin (Table 1 and Additional file 1: Tables S2 and 4).

**Table 1:** Functional categories of common proteins identified in all normoxic ADSC secretomes

GO term	Count^a^	%	*P* value**	Proteins (Uniprot acc no.)	Examples
GO:0005578 ~ proteinaceous extracellular matrix	41	41.4	<0.001	P02751, P05997, P01033, P20908, P27797, Q99715, P02461, Q16610, P16035, P09486, P28300, P07942, P11047, P98160, P98095, P12110, P14543, P12111, Q9UBX5, P02452, P23142, P55268, Q16363, P35555, Q15582, P08123, O95967, P07585, P24821, P51884, P12109, P08254, P08253, Q08629, P21810, Q12805, Q15063, Q08380, Q14767, P13611, P03956	Collagens, collagen-maturation enzymes, collagen interacting proteins, elastin-associated molecules, matricellular proteins, laminins
GO:0005509 ~ calcium ion binding	35	35.4	<0.001	Q02818, P35442, P19022, P27797, P09871, Q92626, P12814, P09486, P11021, O43707, P07996, P98095, P00736, P14543, Q9UBX5, Q15293, P23142, Q12841, P80303, P15289, P35555, O95967, O94985, Q9BRK5, P06396, P13497, P08254, P08253, Q08629, P67936, Q12805, O43852, Q14767, P13611, P03956	calsyntenin 1; heat shock 70 kDa protein 5; reticulocalbin 1; stromal cell derived factor 4; calumenin; nucleobindin 1,2; calreticulin
GO:0007155 ~ cell adhesion	31	31.3	<0.001	Q16270, P35442, P02751, P20908, P19022, Q99715, P02461, P12814, P07942, P11047, P07996, P98160, P12110, P12111, P14543, Q9UBX5, P05067, P55268, Q16363, Q15582, P35579, O94985, P24821, Q8IUX7, P13497, P12109, Q08629, Q15063, Q08380, O14498, P13611	Lectin 3 binding protein; BMP1; AE binding protein 1;N-cadherin; IGFBP-7; TGFBIP
GO:0006928 ~ cell motion	14	14.1	<0.001	P02751, P20908, P19022, P60709, P05067, P35579, Q13822, P09493, P06753, Q08629, P67936, P11047, P07996, P13611	Extracellular lysophospholipase D; tropomyosins 1, 3, 4
GO:0008233 ~ peptidase activity	13	13.1	<0.001	P30101, O14773, P16870, P07858, P09871, P05067, Q8IUX7, P13497, P08254, P08253, P05121, P03956, P00736	MMP1, 2; carboxypeptidase E; cathepsin B; tripeptidyl peptidase I
GO:0004866 ~ endopeptidase inhibitor activity	12	12.1	<0.001	P16035, P36955, P01033, P12111, P05155, P05121, P11021, P50454, P05067, P07093, Q6YHK3, P01034	PEDF; TIMP1,2; PAI-1, 2; cystatin C
GO:0001568 ~ blood vessel development	11	11.1	<0.001	P20908, P19022, P08253, P02452, P28300, P21810, Q16363, P02461, P07996, P35579, P08123	N-cadherin; thrombospondin 1, 2; biglycan; lysyl oxidase
GO:0042060 ~ wound healing	10	10.1	<0.001	P02751, P09493, P20908, P05155, P06396, P05121, Q9UBX5, P28300, P02461, O95967	Fibulins 4, 5; collagen, type III; fibronectin 1
GO:0006955 ~ immune response	10	10.1	0.037	P61769, P05155, P26022, O75326, P10909, P09871, Q92626, P07996, Q13822, P00736	pentraxin 3; Sem7A; β2-microglobulin; clusterin
GO:0060348 ~ bone development	7	7.1	<0.001	P05997, P13497, P08253, P09486, P02452, P17936, P98160	Perlecan; osteonectin; BMP1; IGFBP 3
GO:0031175 ~ neuron projection development	6	6.1	0.027	P10909, P07942, P60709, P05067, P55268, P13611	Versican; amyloid beta (A4) precursor protein
GO:0060284 ~ regulation of cell development	5	5.1	0.046	P16035, P36955, P19022, P27797, P17936	N-cadherin; PEDF; TIMP2; IGFBP 3
GO:0031099 ~ regeneration	4	4	0.011	P06396, P05121, P55268, P13611	PAI-1, laminin S; gelsolin
GO:0007178 ~ transmembrane receptor protein serine/threonine kinase signaling pathway	4	4	0.030	Q12841, P02461, P08123, Q14767	CD109; DKK3; follistatin-like 1
GO:0016860 ~ intramolecular oxidoreductase activity	3	3	0.027	P30101, P07237, P41222	prolyl 4-hydroxylase; prostaglandin D2 synthase

*ADSC* adipose-derived mesenchymal stromal cells, *GO* Gene Ontogeny, *TGFBIP* transforming growth factor, beta-induced, 68 kDa, *IGFBP* insulin-like growth factor binding protein

^a^Proteins annotated to different functional clusters partially overlap

**p value according to kappa statistics (DAVID Bioinformatics Resources, https://david.ncifcrf.gov) [22, 47]

In addition to common proteins, ADSCs from different subjects secreted proteins, which varied between different cultures (Additional file 1: Table S4). Together common and variable proteins secreted by ADSCs fit over 400 GO terms with *p* value <0.05. Interestingly, terms with the highest protein counts matched GO terms identified for common proteins. Thus, 101 ECM proteins were identified in ADSCs secretomes (GO:0005578 ~ proteinaceous extracellular matrix, see Additional file 1: Table S5 for proteins list), which corresponds to 30 % of all known human proteins included in this category. Overall, ADSCs secretomes contained 41 proteins involved in blood vessel development (see Additional file 1: Table S6 for proteins list) and 24 proteins grouped into GO:0030182 ~ neuron differentiation term (see Additional file 1: Table S7). These included angiopoietin-like 2 and −4, gremlin-1 and −2, inhibin A, IGFBP 3–7 and −10, IL-6 and −8, SCF, MIF, urokinase, SDF-4, PDGF-D and VEGF-C. Several proteins, which were not previously associated with ADSCs were also found in their secretomes. These included proteins with neurotrophic activities, such as caprin 1, mesencephalic astrocyte-derived neurotrophic factor, meteorin and neuron derived neurotrophic factor.

### ADSCs secretome content is largely unaffected by prolonged hypoxia

**Fig. 2:**
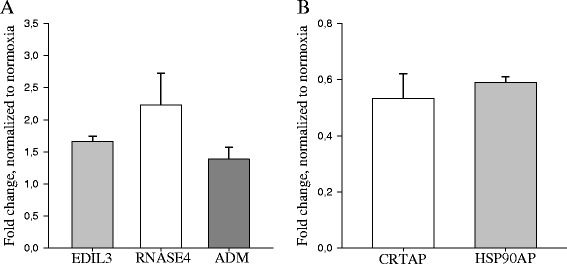
Hypoxia-induced changes of mRNAs, encoding overrepresented proteins. **a** mRNAs encoding proteins overrepresented in hypoxic secretomes. **b** mRNAs encoding proteins overrepresented in normoxic secretomes. mRNA fold changes are expressed using 2^-ΔΔCt^, n = 18

**Fig. 3:**
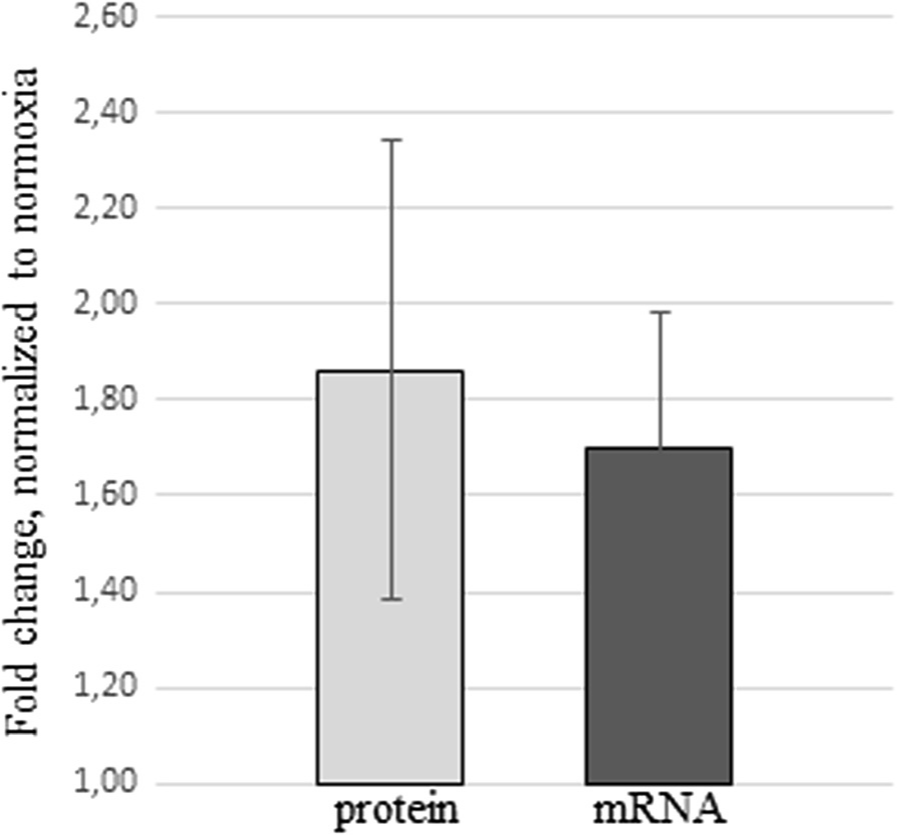
Hypoxia-induced changes of G-CSF mRNA and protein in ADSCs secretomes. Protein concentration was measured by ELISA and expressed as a mean of fold changes calculated for each donor. mRNA fold changes are expressed using 2^-ΔΔCt^, n = 16 for both analyses. *G-CSF* granulocyte-colony stimulating factor, *ADSCs* adipose-derived mesenchymal stromal cells, *ELISA* enzyme-linked immunosorbent assay

To enhance the secretion of proteins involved in tissue regeneration, we subjected ADSCs to hypoxia (1 % O_2_ for 48 h) [18]. Cells responded to hypoxia in a similar manner, by 5.6 ± 0.4 fold up-regulation of the content of HIF-1α (Additional file 5: Figure S3) as well as by 2.82 ± 1.28 fold elevation of known HIF-1α target VEGFA mRNA. In secretomes of hypoxic ADSCs 616 secretory proteins were identified; 480 of these were considered to be secreted through a classical pathway, while 142 proteins were secreted through nonclassical pathways (Table 2, Additional file 5: Figure S3). Overall, ADSC incubation in hypoxia resulted in secretion of six proteins which were not found in normoxic secretomes (Table 3). These included proteins involved in the regulation of angiogenesis, such as EGF-like repeats and discoidin I-like domains 3 (EDIL), ribonuclease 4 of RNase A family and adrenomedullin. Eight proteins were found in hypoxic samples less frequently compared to normoxia (Table 3). Using real-time PCR we confirmed that hypoxia has affected the expression of corresponding mRNAs (Fig. 2). We also examined if hypoxia affects the expression of rarely detected proteins such as G-CSF, which was found only in one hypoxic but not in normoxic secretomes. Concentration of G-CSF in secretomes of normoxic ADSC was 13.4 ± 8.5 fmol, which is close to the detection limit of LS/MS analysis. In hypoxia, G-CSF mRNA and protein were up-regulated 1.7 ± 0.3 and 1.9 ± 0.5 fold, respectively (Fig. 3).

**Table 2:** ADSC secretomes overview

Parameter	Normoxia	Hypoxia
Total number of proteins found in secretomes	608	620
Number of common proteins shared by all secretomes	100	79
Number of classically secreted proteins	451	480
Number of proteins secreted via nonclassical pathways	157	140
Number of overrepresented proteins*	7	2
Number of unique proteins^a^	1	4

*ADSC* adipose-derived mesenchymal stromal cells

^a^proteins found only in normoxic or hypoxic secretomes (>3 samples, *p* >0.1)

*p <0.1 (hypergeometric test)

**Table 3:** Overrepresented proteins in secretomes of normoxic or hypoxic ADSCs

Uniprot_acc #	Protein name	*P* value
Proteins overrepresented in secretomes of normoxic ADSCs		
P35556	fibrillin 2	0.08
P35052	glypican 1	0.07
P29401	transketolase	0.08
O75718	cartilage associated protein	0.04
P48723	heat shock protein 70 kDa family, member 13	0.08
Q14257	reticulocalbin 2, EF-hand calcium binding domain	0.08
P30048	peroxiredoxin 3	0.08
Q9UL46	proteasome activator subunit 2 (PA28 beta)	0.07
Proteins overrepresented in secretomes of hypoxic ADSCs		
Q8N6G6	ADAMTS-like 1	0.04
P19823	inter-alpha (globulin) inhibitor H2	0.04
O43854	EGF-like repeats and discoidin I-like domains 3	0.1
P34096	ribonuclease, RNase A family, 4	0.1
P35318	adrenomedullin	0.1
P78324	Tyrosine-protein phosphatase non-receptor type substrate 1 (SIPRA)	0.1

Protein distribution by functional clusters in hypoxic secretomes was similar to normoxia and the rank order or significance of functional clusters did change between normoxic and hypoxic conditions. The most represented group was GO:0005578 ~ proteinaceous extracellular matrix (98 proteins). Within this cluster four additional proteins were detected, which have not been found in normoxic secretomes. However, these were found only in one or two secretomes each (of different donors); therefore, this finding must be considered with caution. At the same time, seven ECM proteins, which have been detected in normoxic secretomes were not found in hypoxic samples (Additional file 1: Table S5). The disappearence of only one of these, namely cartilage associated protein (CRTAP) was significant (Table 3); other proteins were found only in one or two normoxic secretomes each.

## Discussion

Since ADSCs are promising material for cell therapy and tissue engineering, their secretome has been analyzed in many studies using various qualitative and quantitative approaches (for details see [8, 23] and references therein) in order to understand the physiology of these cells. Our data are consistent with published observations regarding ADSCs secretome content and provide an expansion of known catalogues of proteins secreted by ADSCs. Thus, 75 % of identified secretory proteins possess a signal peptide and, therefore, are likely to be secreted by classical ER-Golgi apparatus pathway [24]. Other proteins are suggested to undergo unconventional secretion, via non-vesicular and vesicular pathways [25]. For example, annexin A2, which was found in the most secretomes analyzed in the current study, is produced via direct translocation across the plasma membrane [26]. ADSCs secretomes also contain proteins secreted via exosomes (e.g., inhibin A and SCF [7]) and through so called “Golgi bypass” (eg., serglycin [27]).

Most previous proteomic studies have analyzed the content of pooled secretomes rather than of samples obtained from individual donors [8, 23]. This study indicates that despite similar characteristics, which are routinely assessed for ADSCs, including their differentiation abilities and stromal immunophenotype [19], these cells can differ in the spectrum of produced proteins. Such differences could be explained by the presence of multiple subtypes specialized in the production of particular proteins. Here we demonstrate that analyzed ADSCs cultures differ in the proportion of cells expressing CD146 (MCAM) surface antigen. Furthermore, other membrane proteins, including receptors for hormones and neuromediators could possibly serve as definitive markers for such ADSC subpopulations [28]. Future proteomic analysis of ADSC subpopulations purified according to the expression of those markers is important as it can reveal cells with specialized activities. The usage of such purified cells will allow an increase in the efficiency of ADSC clinical applications and reduce possible side effects.

Consistent with other reports, the largest functional cluster in the ADSC secretome is comprised of ECM proteins. Such protein profile is in line with the stromal characteristics of ADSCs. Adequate production of ECM components is necessary for tissue homeostasis and regeneration [29, 30], because these molecules not only provide a scaffold for cells and soluble molecules, but also regulate angiogenesis and inflammation (i.e., thrombospondin 1 [31], periostin [32], and collagen-derived peptides).

Among proteins common to all ADSC secretomes several molecules not previously associated with ADSCs were identified, including DKK3, olfactomedin-like 3, follistatin-like 1, and prosaposin. These molecules might also contribute to tissue morphogenesis and regeneration induced by ADSCs [33–35]. For example, olfactomedin-like 3 was demonstrated to be important for tumor angiogenesis and vessel stabilization [34]. Considering these data, we can suggest that olfactomedin-like 3 is important for the ability of ADSCs to stimulate the growth of blood vessels. Among variably present molecules, we found several proteins with neurotrophic activities, such as caprin 1, mesencephalic astrocyte-derived neurotrophic factor, meteorin, and neuron derived neurotrophic factor. These factors, together with neurotrophins previously found in ADSCs conditioned medium, including BDNF, NGF and GDNF may account for the beneficial action of these cells or their secretomes on the regeneration of brain and peripheral nerves [4, 6]. Uncovering the content of neurotrophins produced by ADSCs allows the development of a defined preparation of recombinant factors, which might substitute cell therapy in the treatment of neurodegenerative disorders in the future. Such enrichment of ADSCs secretome with neurotrophins is not surprising and is in line with the location of MSCs in close proximity to peripheral nerves in vivo [36].

The in vivo decrease of oxygen tension below its physiological level (2-9 % O_2_ for ADSCs) can trigger regeneration processes [37–39]. Cells within a damaged area respond to hypoxia by stabilization of hypoxia inducible factor-1 (HIF-1α) [40], which binds to HIF-1 response elements and drives the expression of mRNA and miR encoding genes [41, 42]. In our study we demonstrate that hypoxia stimulates the expression and secretion of angiogenic proteins, including EGF-like EDIL, RNASE4 and adrenomedullin. Ribonuclease 4 was not previously reported as a target of HIF-1a; however, it shares a common promoter with angiogenin, which is regulated by this transcription factor. Previously, angiogenin was found in MSC secretomes [12]. Here we demonstrate that RNASE4 prevails in the ADSCs secretome and might be responsible for angiogenic and anti-apoptotic activities of these cells [43]. This is consistent with known mechanisms of long-term adaptation to hypoxia and supports our previous findings suggesting that hypoxia causes simultaneous up-regulation of angiogenesis stimulators together with down-regulation of its inhibitors in ADSCs [3, 16]. Prolonged hypoxia also led to the disappearance of proteins regulating osteogenesis and ECM remodeling, such as cartilage-associated protein (uniprot ID#O75718) and osteoglycin (uniprot ID #P20774). This is consistent with previous reports, indicating that low O_2_ concentration suppresses osteogenic differentiation of MSCs [44, 45]. This is also in line with a study of Fraizer TP et al., which nicely quantified the level of proteins in the ADSCs secretomes and demonstrates that exposure of these cells to low O_2_ reduces ECM components [46].

## Conclusions

Taken together our results suggest that human ADSCs, defined as CD90+/CD73+/CD105+/CD45-/CD31-/PDGFRbeta+/NG2+/CD146 + (−) cells, secrete a large array of proteins, the most represented group being comprised of extracellular matrix components. The number of secreted proteins is largely unaffected by prolonged hypoxia. Variability in the secretion of several proteins from cultured ADSCs of individual subjects suggests that these cells exist as a heterogeneous population containing functionally distinct subtypes, which differ in numbers between donors. Despite the fact that this requires further investigation, the present study provides a basis for further elucidation of the functional heterogeneity of these cells.

## Additional files

Additional file 1: Table S1. ADSC donor and culture characteristics. **Table S2.** Common proteins identified in all secretomes of normoxic ADSCs. **Table S4.** Proteins identified in 1–10 secretomes of normoxic ADSCs. **Table S5.** List of extracellular matrix proteins found in secretomes of normoxic ADSCs. **Table S6.** List of proteins involved in blood vessel development, which were found in secretomes of normoxic ADSCs. **Table S7.** List of proteins involved in neuron differentiation, which were found in secretomes of normoxic ADSCs. (DOCX 118 kb) Additional file 2: Figure S1. Representative images of ADSCs undergoing adipogenic or osteogenic differentiation. A1-A7. – Cells incubated in adipogenesis inducing medium for 27 days. C1-C7. - Cells incubated in osteogenesis inducing medium for 21 day. A, B. – Cells stained with Oil-red O to detect lipid accumulations. C, D. - Cells stained with Alizarin red S to detect calcium accumulations. Nuclei were counterstained by hematoxilin. Scale = 100 μm. (TIFF 1643 kb) Additional file 3: Figure S2. Representative flow cytometry charts and plots of additional surface markers expression on ADSC obtained from eight donors (#2-9). A. - Expression of pericyte markers, PDGFRβ and NG2 glycoprotein. Pink areas – specific antibodies, blue areas – isotype antibodies. B. – Expression of CD146 together with CD34 or CD31. (TIFF 262 kb) Additional file 4: Table S3. Data of transcriptome profiling of normoxic ADSCs. (DOCX 87 kb) Additional file 5: Figure S3. ADSC response to hypoxia. A. – Representative Western blots showing the accumulation of HIF-1alpha after the incubation at 1 % O_2_ during 48 h. B. – Real time PCR analysis of VEGF mRNA in hypoxic and normoxic ADSCs (n = 20, * - *p* <0.05 vs. normoxia). (TIFF 100 kb)

## Abbreviations

ADSCs: Adipose-derived mesenchymal stromal cells
BDNF: Brain derived neurotrophic factor
DKK3: Dickkopf-related protein
DTT: Dithiothreitol
ECM: Extracellular matrix
EGF: Epidermal growth factor
G-CSF: Granulocyte-colony stimulating factor
GDNF: Glial cell derived neurotrophic factor
HGF: Hepatocyte growth factor
HIF-1: Hypoxia inducible factor-1
IGF-1: Insulin-like growth factor
IGFBP: Insulin-like growth factor-binding protein
IL: Interleukin
LS/MS: Liquid chromatography/mass spectrometry
MCAM: Melanoma cell adhesion molecule
M-CSF: Monocyte colony stimulating factor
MEM: Minimum essential medium
MIF: Macrophage migration inhibitory factor
NG2: Neural/glial antigen 2
NGF: Beta-nerve growth factor
PDGF: Platelet-derived growth factor
PDGFRβ: platelet-derived growth factor receptor beta
PEDF: Pigment epithelium-derived factor
RNASE4: Ribonuclease 4
SCF: Stem cell factor
SDF: Stromal cell derived factor
VEGF: Vascular endothelial growth factor
