# Supplementary material for: Characterization of secretomes provides evidence for adipose-derived mesenchymal stromal cells subtypes
Source: Stem Cell Res Ther. 2015 Nov 11;6:221. doi: 10.1186/s13287-015-0209-8 (PMC4642680; doi:10.1186/s13287-015-0209-8)
Supplement: Additional file 1: Table S1. — ADSC donor and culture characteristics. Table S2. Common proteins identified in all secretomes of normoxic ADSCs. Table S4. Proteins identified in 1–10 secretomes of normoxic ADSCs. Table S5. List of extracellular matrix proteins found in secretomes of normoxic ADSCs. Table S6. List of proteins involved in blood vessel development, which were found in secretomes of normoxic ADSCs. Table S7. List of proteins involved in neuron differentiation, which were found in secretomes of normoxic ADSCs. (DOCX 118 kb) [file 13287_2015_209_MOESM1_ESM.docx]

**Table S1. ADSC donor and cultures characteristics**

| **Donor** | **Age** | **BMI** | **Race** | **ADSC cultures** | | |
| --- | --- | --- | --- | --- | --- | --- |
|  |  |  |  | doubling time, hrs | osteogenic differentiation | adipogenic differentiation |
| 1 | 43 | 21 | Caucasian | 106 | + | + |
| 2 | 28 | 20 | Caucasian | 80 | + | + |
| 3 | 35 | 21 | Caucasian | 121 | + | + |
| 4 | 48 | 24 | Caucasian | 71 | + | + |
| 5 | 40 | 25 | Caucasian | 165 | + | + |
| 6 | 43 | 23 | Caucasian | 118 | + | + |
| 7 | 23 | 22 | Caucasian | 148 | + | + |
| 8 | 46 | 28 | Caucasian | 68 | + | + |
| 9 | 47 | 26 | Caucasian | 95 | + | + |
| 10 | 38 | 24 | Caucasian | 120 | + | + |

**Table S2. Common proteins identified in all secretomes of normoxic ADSCs**

| **UNIPROT_ACC** | **PROTEIN NAME** | **BIOLOGICAL FUNCTION*** |  |
| --- | --- | --- | --- |
| **Proteinaceous extracellular matrix GO:0005578** | | | |
| **Collagens** | | | |
| P02452 | collagen, type I, alpha 1 | Members of group I collagen (fibrillar forming collagen). |  |
| P08123 | collagen, type I, alpha 2 |  |  |
| P02461 | collagen, type III, alpha 1 | Collagen type III occurs in most soft connective tissues along with type I collagen. Involved in regulation of cortical development. Is the major ligand of GPR56 in the developing brain and binding to GPR56 inhibits neuronal migration and activates the RhoA pathway by coupling GPR56 to GNA13 and possibly GNA12. |  |
| P20908 | collagen, type V, alpha 1 | Type V collagen is a member of group I collagen (fibrillar forming collagen). It is a minor connective tissue component of nearly ubiquitous distribution. Type V collagen binds to DNA, heparan sulfate, thrombospondin, heparin, and insulin |  |
| P05997 | collagen, type V, alpha 2 |  |  |
| P12109 | collagen, type VI, alpha 1 | I acts as a cell-binding protein.  This isoform is involved in control of adipocyte hypertrophy and obesity [39]. |  |
| P12110 | collagen, type VI, alpha 2 |  |  |
| P12111 | collagen, type VI, alpha 3 |  |  |
| P27658 | collagen, type VIII,  alpha 1 | Major component of the basement membrane of endothelial cells. Necessary for migration and proliferation of vascular smooth muscle cells and thus, has a potential role in the maintenance of vessel wall integrity and structure. Vastatin, the C-terminal fragment comprising the NC1 domain, inhibits aortic endothelial cell proliferation. | |
| Q99715 | collagen, type XII, alpha 1 | Type XII collagen interacts with type I collagen-containing fibrils, the COL1 domain could be associated with the surface of the fibrils, and the COL2 and NC3 domains may be localized in the perifibrillar matrix | |
| **Collagen maturation enzymes** | | | |
| Q02809 | procollagen-lysine 1, 2-oxoglutarate 5-dioxygenase 1 | Forms hydroxylysine residues in -Xaa-Lys-Gly- sequences in collagens. These hydroxylysines serve as sites of attachment for carbohydrate units and are essential for the stability of the intermolecular collagen cross-links. | |
| Q15113 | procollagen C-endopeptidase enhancer | Binds to the C-terminal propeptide of type I procollagen and enhances procollagen C-proteinase activity.  C-terminal processed part of PCPE (CT-PCPE) may have an metalloproteinase inhibitory activity. | |
| P28300 | Protein-lysine 6-oxidase | Post-translational oxidative deamination of peptidyl lysine residues in precursors to fibrous collagen and elastin. In addition to cross-linking of extracellular matrix proteins, may have a direct role in tumor suppression. | |
| P13497 | bone morphogenetic protein 1 | Cleaves the C-terminal propeptides of procollagen I, II and III. Induces cartilage and bone formation. Responsible for the proteolytic activation of lysyl oxidase. | |
| **Collagen interacting proteins** | | | |
| P50454 | serpin peptidase inhibitor, clade H (collagen binding protein 1) | Binds specifically to collagen. Could be involved as a chaperone in the biosynthetic pathway of collagen. | |
| Q15582 | transforming growth factor, beta-induced, 68kDa | Binds to type I, II, and IV collagens. This adhesion protein may play an important role in cell-collagen interactions. In cartilage, may be involved in endochondral bone formation. | |
| **Elastin-associated molecules** | | | |
| Q12805 | EGF-containing fibulin-like extracellular matrix protein 1 | Binds EGFR, the EGF receptor, inducing EGFR autophosphorylation and the activation of downstream signaling pathways. May play a role in cell adhesion and migration. May function as a negative regulator of chondrocyte differentiation. May regulate glial cell migration, differentiation and neurite outgrowth. | |
| O95967 | EGF-containing fibulin-like extracellular matrix protein 2 | Molecules associated with elastic fibres | |
| P35555 | fibrillin 1 | Fibrillins are structural components of 10-12 nm extracellular calcium-binding microfibrils, which occur either in association with elastin or in elastin-free bundles. Fibrillin-1-containing microfibrils provide long-term force bearing structural support. Regulates osteoblast maturation by controlling TGF-beta bioavailability and calibrating TGF-beta and BMP levels, respectively | |
| Q14767 | latent transforming growth factor beta binding protein 2 | May play an integral structural role in elastic-fiber architectural organization and/or assembly. | |
| **Matricellular proteins** | | | |
| P21810 | biglycan | Small leucine-rich proteoglycan. May be involved in collagen fiber assembly. Regulates angiogenesis and bone formation by depositing VEGF-A [40]. | |
| P07585 | decorin | Stromal-specific proteoglycan. Small leucine-rich proteoglycan. May affect the rate of collagen fibrils formation. | |
| P02751 | fibronectin 1 | Fibronectins bind cell surfaces and various compounds including collagen, fibrin, heparin, DNA, and actin. Fibronectins are involved in cell adhesion, cell motility, opsonization, wound healing, and maintenance of cell shape. Involved in osteoblast compaction through the fibronectin fibrillogenesis cell-mediated matrix assembly process, essential for osteoblast mineralization. Participates in the regulation of type I collagen deposition by osteoblasts. | |
| P23142 | fibulin 1 | Incorporated into fibronectin-containing matrix fibers. May play a role in cell adhesion and migration along protein fibers within the extracellular matrix (ECM). Could be important for certain developmental processes and contribute to the supramolecular organization of ECM architecture, in particular to those of basement membranes. Could play a significant role in modulating the neurotrophic activities of APP, particularly soluble APP | |
| P98095 | fibulin 2 | Its binding to fibronectin and some other ligands is calcium dependent. | |
| Q9UBX5 | fibulin 5 | Promotes adhesion of endothelial cells through interaction of integrins and the RGD motif. Could be a vascular ligand for integrin receptors and may play a role in vascular development and remodeling. | |
| P51884 | lumican | Small leucine-rich proteoglycan. Promotes chemokines secretion. | |
| Q08380 | lectin, galactoside-binding, soluble, 3 binding protein | Promotes intergrin-mediated cell adhesion. May stimulate host defense against viruses and tumor cells. | |
| P14543 | nidogen 1 | Sulfated glycoprotein widely distributed in basement membranes and tightly associated with laminin. Also binds to collagen IV and perlecan. It probably has a role in cell-extracellular matrix interactions. | |
| P09486 | osteonectin | Appears to regulate cell growth through interactions with the extracellular matrix and cytokines. Binds calcium and copper, several types of collagen, albumin, thrombospondin, PDGF and cell membranes. | |
| Q15063 | periostin, osteoblast specific factor | Enhances incorporation of BMP1 in the fibronectin matrix of connective tissues, and subsequent proteolytic activation of lysyl oxidase LOX. | |
| P98160 | perlecan | Integral component of basement membranes. Plays essential roles in vascularization. Critical for normal heart development and for regulating the vascular response to injury. Its C-terminus peptides endorepellin and LG3 have anti-angiogenic properties. | |
| Q08629 | sparc/osteonectin, cwcv and kazal-like domains proteoglycan (testican) 1 | May play a role in cell-cell and cell-matrix interactions. May contribute to various neuronal mechanisms in the central nervous system. | |
| P24821 | tenascin C | Extracellular matrix protein implicated in guidance of migrating neurons as well as axons during development, synaptic plasticity as well as neuronal regeneration. Promotes neurite outgrowth from cortical neurons grown on a monolayer of astrocytes. Ligand for integrins alpha-8/beta-1, alpha-9/beta-1, alpha-V/beta-3 and alpha-V/beta-6. | |
| P07996 | thrombospondin 1 | Adhesive glycoprotein that mediates cell-to-cell and cell-to-matrix interactions. Binds heparin. May play a role in dentinogenesis and/or maintenance of dentin and dental pulp By similarity. Ligand for CD36 mediating antiangiogenic properties. Plays a role in ER stress response, via its interaction with the activating transcription factor 6 alpha (ATF6) which produces adaptive ER stress response factors | |
| P35442 | thrombospondin 2 | Adhesive glycoprotein that mediates cell-to-cell and cell-to-matrix interactions. Ligand for CD36 mediating antiangiogenic properties | |
| P13611 | versican | May play a role in intercellular signaling and in connecting cells with the extracellular matrix. May take part in the regulation of cell motility, growth and differentiation. Binds hyaluronic acid. | |
| **Laminins** | | | |
| Q16363 | laminin, alpha 4 | Binding to cells via a high affinity receptor, laminin is thought to mediate the attachment, migration and organization of cells into tissues during embryonic development by interacting with other extracellular matrix components. | |
| P07942 | laminin, beta 1 |  |  |
| P55268 | laminin, beta 2 (laminin S) |  |  |
| P11047 | laminin, gamma 1 |  |  |
| **Peptidase activity GO:0008233** | | | |
| P16870 | carboxypeptidase E | Removes residual C-terminal Arg or Lys remaining after initial endoprotease cleavage during prohormone processing. Processes proinsulin. | |
| P07858 | cathepsin B | Thiol protease which is believed to participate in intracellular degradation and turnover of proteins. Has also been implicated in tumor invasion and metastasis. | |
| P03956 | matrix metallopeptidase 1 (interstitial collagenase) | Cleaves collagens of types I, II, and III at one site in the helical domain. Also cleaves collagens of types VII and X. | |
| P08253 | matrix metallopeptidase 2 (gelatinase A, 72kDa gelatinase, 72kDa type IV collagenase) | Ubiquitinous metalloproteinase that is involved in diverse functions such as remodeling of the vasculature, angiogenesis, tissue repair, tumor invasion, inflammation, and atherosclerotic plaque rupture. Involved in the formation of the fibrovascular tissues in association with MMP14. PEX, the C-terminal non-catalytic fragment of MMP2, posseses anti-angiogenic and anti-tumor properties and inhibits cell migration and cell adhesion to FGF2 and vitronectin. Ligand for integrinv/beta3 on the surface of blood vessels. | |
| P08254 | matrix metallopeptidase 3 (stromelysin 1, progelatinase) | Can degrade fibronectin, laminin, gelatins of type I, III, IV, and V; collagens III, IV, X, and IX, and cartilage proteoglycans. Activates procollagenase. | |
| P30086 | phosphatidylethanolamine binding protein 1 | Serine protease inhibitor which inhibits thrombin, neuropsin and chymotrypsin but not trypsin, tissue type plasminogen activator and elastase. | |
| O14773 | tripeptidyl peptidase I | Lysosomal serine protease with tripeptidyl-peptidase I activity. May act as a non-specific lysosomal peptidase which generates tripeptides from the breakdown products produced by lysosomal proteinases. Requires substrates with an unsubstituted N-terminus | |
| Q8IUX7 | AE binding protein 1 | May positively regulate MAP-kinase activity in adipocytes, leading to enhanced adipocyte proliferation and reduced adipocyte differentiation. | |
| **Endopeptidase inhibitor activity GO:0004866** | | | |
| P01034 | cystatin C | Act as an inhibitor of cysteine proteinases | |
| P05121 | serpin peptidase inhibitor, clade E (nexin, plasminogen activator inhibitor type 1), member 1 | Serine protease inhibitor. This inhibitor acts as 'bait' for tissue plasminogen activator, urokinase, protein C and matriptase-3/TMPRSS7. Its rapid interaction with PLAT may function as a major control point in the regulation of fibrinolysis. | |
| P07093 | serpin peptidase inhibitor, clade E (nexin, plasminogen activator inhibitor type 1), member 2 | Serine protease inhibitor with activity toward thrombin, trypsin, and urokinase. Promotes neurite extension by inhibiting thrombin. Binds heparin. | |
| P01033 | TIMP1 metallopeptidase inhibitor 1 | Metalloproteinase inhibitor that functions by forming one to one complexes with target metalloproteinases, such as collagenases, and irreversibly inactivates them by binding to their catalytic zinc cofactor. Acts on MMP1, MMP2, MMP3, MMP7, MMP8, MMP9, MMP10, MMP11, MMP12, MMP13 and MMP16. Does not act on MMP14. Also functions as a growth factor that regulates cell differentiation, migration and cell death and activates cellular signaling cascades via CD63 and ITGB1. Plays a role in integrin signaling. | |
| P16035 | TIMP metallopeptidase inhibitor 2 | Complexes with metalloproteinases (such as collagenases) and irreversibly inactivates them by binding to their catalytic zinc cofactor. Known to act on MMP-1, MMP-2, MMP-3, MMP-7, MMP-8, MMP-9, MMP-10, MMP-13, MMP-14, MMP-15, MMP-16 and MMP-19 | |
| **Regulation of growth GO:0040008** | | | |
| P09603 | colony stimulating factor 1 (macrophage) | Promotes the release of proinflammatory chemokines, and thereby plays an important role in innate immunity and in inflammatory processes. Plays an important role in the regulation of osteoclast proliferation and differentiation, the regulation of bone resorption, and is required for normal bone development. | |
| P05067 | amyloid beta (A4) precursor protein | On the surface of neurons performs functions relevant to neurite growth, neuronal adhesion and axonogenesis. Can regulate neurite outgrowth through binding to heparin and collagen I and IV. | |
| P17936 | insulin-like growth factor binding protein 3 | IGF-binding proteins prolong the half-life of the IGFs and have been shown to either inhibit or stimulate the growth promoting effects of the IGFs on cell culture. They alter the interaction of IGFs with their cell surface receptors. | |
| Q16270 | insulin-like growth factor binding protein 7 | Binds IGF-I and IGF-II with a relatively low affinity. Stimulates prostacyclin (PGI2) production. Stimulates cell adhesion. | |
| **Regulation of neurogenesis GO:0050767** | | | |
| P36955 | pigment epithelium derived factor | Neurotrophic protein; induces extensive neuronal differentiation in retinoblastoma cells. Potent inhibitor of angiogenesis. | |
| P19022 | cadherin 2, type 1, N-cadherin (neuronal) | Homophilic cell adhesion. CDH2 may be involved in neuronal recognition mechanism. In hippocampal neurons, may regulate dendritic spine density | |
| Q9NRN5 | olfactomedin-like 3 | Secreted scaffold protein that plays an essential role in dorsoventral patterning during early development. Proangiogenic [27] Inhibits neurites outgrowth, modulates Wnt and Notch signaling | |
| **Immune response GO:0006955** | | | |
| P61769 | beta-2-microglobulin | Component of the class I major histocompatibility complex (MHC). Involved in the presentation of peptide antigens to the immune system. | |
| P10909 | clusterin | Functions as extracellular chaperone that prevents aggregation of nonnative proteins. Prevents stress-induced aggregation of blood plasma proteins. Inhibits formation of amyloid fibrils by APP. | |
| P00736 | complement component 1, r subcomponent | C1r B chain is a serine protease that combines with C1q and C1s to form C1, the first component of the classical pathway of the complement system. | |
| P09871 | complement component 1, s subcomponent |  |  |
| Q13822 | ectonucleotide pyrophosphatase/  phosphodiesterase 2 | Hydrolyzes lysophospholipids to produce lysophosphatidic acid (LPA) in extracellular fluids. Produces sphingosine-1-phosphate, a modulator of cell motility during angiogenesis and neurite outgrowth. Acts as an angiogenic factor by stimulating migration of smooth muscle cells and microtubule formation. Possible involvement in cell proliferation and adipose tissue development. | |
| P26022 | pentraxin-related gene, rapidly induced by IL-1 beta | Plays a role in the regulation of innate resistance to pathogens, inflammatory reactions, possibly clearance of self-components and female fertility | |
| Q92626 | peroxidasin homolog (Drosophila) | Displays low peroxidase activity and is likely to participate in H_2_O_2_ metabolism and peroxidative reactions in the cardiovascular system. Plays a role in extracellular matrix formation | |
| O75326 | semaphorin 7A, GPI membrane anchor (John Milton Hagen blood group) | Plays an important role in integrin-mediated signaling and functions both in regulating cell migration and immune responses. Promotes formation of focal adhesion complexes, activation of the protein kinase PTK2/FAK1 and subsequent phosphorylation of MAPK1 and MAPK3. Promotes axon growth in the embryonic olfactory bulb. | |
| P05155 | serpin peptidase inhibitor, clade G (C1 inhibitor), member 1 | Activation of the C1 complex is under control of the C1-inhibitor. It forms a proteolytically inactive stoichiometric complex with the C1r or C1s proteases. May play a potentially crucial role in regulating important physiological pathways including complement activation, blood coagulation, fibrinolysis and the generation of kinins. Very efficient inhibitor of FXIIa. Inhibits chymotrypsin and kallikrein | |
| A1L4H1 | soluble scavenger receptor cysteine-rich domain-containing protein SSC5D | Binds to extracellular matrix proteins. Induces bacterial and fungal aggregation and subsequent inhibition of PAMP-induced cytokine release. May play a role in the innate defense. | |
| **Actin cytoskeleton organization GO:0030036** | | | |
| P60709 | actin, beta | Cytoskeleton component | |
| P12814 | actinin, alpha 1 | F-actin cross-linking protein which is thought to anchor actin to a variety of intracellular structures. This is a bundling protein | |
| O43707 | actinin, alpha 4 | F-actin cross-linking protein which is thought to anchor actin to a variety of intracellular structures. Links MICALL2 to the actin cytoskeleton and recruits it to the tight junctions. | |
| P21333 | filamin A, alpha (actin binding protein 280) | Promotes orthogonal branching of actin filaments and links actin filaments to membrane glycoproteins. Anchors transmembrane proteins to the actin cytoskeleton and serves as a scaffold for a wide range of cytoplasmic signaling proteins. | |
| P06396 | gelsolin (amyloidosis, Finnish type) | Calcium-regulated, actin-modulating protein that binds to the plus (or barbed) ends of actin monomers or filaments, preventing monomer exchange (end-blocking or capping). It can promote the assembly of monomers into filaments (nucleation) as well as sever filaments already formed | |
| P35579 | myosin, heavy chain 9, non-muscle | Cellular myosin that appears to play a role in cytokinesis, cell shape, and specialized functions such as secretion and capping. | |
| P09493 | tropomyosin 1 (alpha) | Binds to actin filaments in muscle and non-muscle cells. Plays a central role, in association with the troponin complex, in the calcium dependent regulation of vertebrate striated muscle contraction. Smooth muscle contraction is regulated by interaction with caldesmon. In non-muscle cells is implicated in stabilizing cytoskeleton actin filaments. | |
| P06753 | tropomyosin 3 |  |  |
| P67936 | tropomyosin 4 |  |  |
| **Secretory granule GO:0030141** | | | |
| P15289 | arylsulfatase A | Hydrolyzes cerebroside sulfate. | |
| P07686 | hexosaminidase B (beta polypeptide) | Responsible for the degradation of GM2 gangliosides, and a variety of other molecules containing terminal N-acetyl hexosamines, in the brain and other tissues. | |
| **Cell surface receptor linked signal transduction GO:0007166** | | | |
| Q6YHK3 | CD109 molecule | Modulates negatively TGFB1 signaling in keratinocytes. Negatively regulates wound healing. | |
| Q9UBP4 | dickkopf homolog 3 (Xenopus laevis) | Antagonizes canonical Wnt signaling by inhibiting LRP5/6 interaction with Wnt and by forming a ternary complex with the transmembrane protein KREMEN that promotes internalization of LRP5/6. Dkks are implicated in bone formation and bone disease, cancer and Alzheimer disease. Promotes fibroblast proliferation and myofibroblast differentiation and regulates expression of angiopoietin-1. | |
| Q12841 | follistatin-like 1 | May modulate the action of some growth factors on cell proliferation and differentiation. A potent regulator of chondrocyte proliferation, differentiation and expression of ECM molecules [28] | |
| **Calcium ion binding GO:0005509** | | | |
| P11021 | hypothetical gene supported by AF216292; NM_005347; heat shock 70kDa protein 5 | Probably plays a role in facilitating the assembly of multimeric protein complexes inside the endoplasmic reticulum. Involved in the correct folding of proteins and degradation of misfolded proteins via its interaction with DNAJC10, probably to facilitate the release of DNAJC10 from its substrate. | |
| Q15293 | reticulocalbin 1, EF-hand calcium binding domain | May regulate calcium-dependent activities in the endoplasmic reticulum lumen or post-ER compartment. | |
| Q9BRK5 | stromal cell derived factor 4 | May regulate calcium-dependent activities in the endoplasmic reticulum lumen or post-ER compartment | |
| O94985 | calsyntenin 1 | Induces KLC1 association with vesicles and functions as a cargo in axonal anterograde transport. Amyloid processing | |
| O43852 | calumenin | Involved in regulation of vitamin K-dependent carboxylation of multiple N-terminal glutamate residues. Seems to inhibit gamma-carboxylase GGCX. | |
| Q02818 | nucleobindin 1 | Major calcium-binding protein of the Golgi. May have a role in calcium homeostasis | |
| P80303 | nucleobindin 2 | Calcium-binding protein. May have a role in calcium homeostasis. | |
| P27797 | calreticulin | Calcium-binding chaperone that promotes folding, oligomeric assembly and quality control in the endoplasmic reticulum (ER) via the calreticulin/calnexin cycle. This lectin interacts transiently with almost all of the monoglucosylated glycoproteins that are synthesized in the ER. | |
| **Intramolecular oxidoreductase activity GO:0016860** | | | |
| P07237 | prolyl 4-hydroxylase, beta polypeptide | At the cell surface, seems to act as a reductase that cleaves disulfide bonds of proteins attached to the cell. At high concentrations, functions as a chaperone that inhibits aggregation of misfolded proteins. At low concentrations, facilitates aggregation (anti-chaperone activity). | |
| P30101 | protein disulfide-isomerase A3 | Resident to ER. Catalyzes the rearrangement of -S-S- bonds in proteins | |
| P41222 | prostaglandin D2 synthase, hematopoietic; prostaglandin D2 synthase 21kDa (brain) | Catalyzes the conversion of PGH2 to PGD2, a prostaglandin involved in smooth muscle contraction/relaxation and a potent inhibitor of platelet aggregation. Binds small non-substrate lipophilic molecules, including biliverdin, bilirubin, retinal, retinoic acid and thyroid hormone, and may act as a scavenger for harmful hydrophopic molecules and as a secretory retinoid and thyroid hormone transporter. | |
| **Unclassified proteins** | | | |
| O14498 | immunoglobulin superfamily containing leucine-rich repeat | Involved in cell adhesion | |
| P07602 | prosaposin | Behaves as a myelinotrophic and neurotrophic factor, these effects are mediated by its G-protein-coupled receptors, GPR37 and GPR37L1, undergoing ligand-mediated internalization followed by ERK phosphorylation signaling | |
| Q4ZHG4 | fibronectin type III domain containing 1 | May be an activator of G protein signaling | |
| O00391 | quiescin Q6 sulfhydryl oxidase 1 | Catalyzes the oxidation of sulfhydryl groups in peptide and protein thiols to disulfides with the reduction of oxygen to hydrogen peroxide. May contribute to disulfide bond formation in a variety of secreted proteins. | |

**Table S4. Proteins identified in 1-10 secretomes of normoxic ADSCs**

| **UNIPROT**  **ACC #** | **Protein Name** | **Number of samples**  **control hypoxia** | | **Number of peptides**  **control**  **M SD** | | **Number of peptides**  **hypoxia**  **M SD** | |
| --- | --- | --- | --- | --- | --- | --- | --- |
| P21589 | 5'-nucleotidase, ecto (CD73) | 3 | 1 | 4,33 | 2,31 | 4,00 | 0,00 |
| Q7Z7G0 | ABI family, member 3 (NESH) binding protein | 9 | 8 | 11,78 | 4,49 | 11,88 | 7,40 |
| O15144 | actin related protein 2/3 complex, subunit 2, 34kDa | 1 | 1 | 4,00 | 0,00 | 3,00 | 0,00 |
| O15511 | actin related protein 2/3 complex, subunit 5, 16kDa | 1 | 0 | 4,00 | 0,00 | 0,00 | 0,00 |
| P60709 | actin, beta | 10 | 8 | 18,80 | 11,23 | 19,13 | 8,94 |
| P12814 | actinin, alpha 1 | 10 | 8 | 23,40 | 8,68 | 24,63 | 7,15 |
| O43707 | actinin, alpha 4 | 10 | 8 | 21,50 | 9,23 | 23,75 | 9,29 |
| O14672 | ADAM metallopeptidase domain 10 | 6 | 5 | 4,17 | 2,79 | 3,80 | 2,49 |
| O43184 | ADAM metallopeptidase domain 12 | 2 | 1 | 2,00 | 0,00 | 2,00 | 0,00 |
| Q13444 | ADAM metallopeptidase domain 15 | 1 | 0 | 2,00 | 0,00 | 0,00 | 0,00 |
| P78536 | ADAM metallopeptidase domain 17 | 2 | 1 | 2,00 | 0,00 | 2,00 | 0,00 |
| Q13443 | ADAM metallopeptidase domain 9 (meltrin gamma) | 8 | 6 | 2,63 | 0,92 | 3,83 | 1,72 |
| Q9UHI8 | ADAM metallopeptidase with thrombospondin type 1 motif, 1 | 1 | 4 | 4,00 | 0,00 | 3,25 | 2,50 |
| O95450 | ADAM metallopeptidase with thrombospondin type 1 motif, 2 | 1 | 3 | 3,00 | 0,00 | 2,33 | 0,58 |
| Q8N6G6 | ADAMTS-like 1 | 2 | 7 | 5,50 | 0,71 | 3,86 | 1,95 |
| O95782 | adaptor-related protein complex 2, alpha 1 subunit | 1 | 1 | 3,00 | 0,00 | 2,00 | 0,00 |
| P55263 | adenosine kinase | 1 | 1 | 2,00 | 0,00 | 2,00 | 0,00 |
| Q8IUX7 | AE binding protein 1 | 10 | 8 | 15,00 | 8,67 | 14,25 | 9,27 |
| P16112 | aggrecan | 4 | 3 | 7,50 | 6,45 | 8,67 | 10,69 |
| O00468 | agrin | 5 | 4 | 11,20 | 7,19 | 11,25 | 6,50 |
| P02768 | albumin | 10 | 9 | 5,70 | 1,89 | 6,67 | 3,24 |
| P04075 | aldolase A, fructose-bisphosphate | 8 | 8 | 8,00 | 3,38 | 7,13 | 3,18 |
| P02765 | alpha-2-HS-glycoprotein | 1 | 1 | 3,00 | 0,00 | 4,00 | 0,00 |
| P01023 | alpha-2-macroglobulin | 9 | 9 | 11,67 | 9,68 | 10,22 | 9,12 |
| P05067 | amyloid beta (A4) precursor protein | 10 | 7 | 6,90 | 4,33 | 9,71 | 4,89 |
| Q06481 | amyloid beta (A4) precursor-like protein 2 | 7 | 6 | 6,43 | 3,05 | 6,17 | 2,99 |
| Q9UKU9 | angiopoietin-like 2 | 3 | 2 | 2,33 | 0,58 | 3,00 | 1,41 |
| Q9BY76 | angiopoietin-like 4 | 1 | 3 | 2,00 | 0,00 | 2,67 | 0,58 |
| P12821 | angiotensin I converting enzyme 1 | 1 | 0 | 2,00 | 0,00 | 0,00 | 0,00 |
| P01019 | angiotensinogen | 1 | 1 | 9,00 | 0,00 | 6,00 | 0,00 |
| P04083 | annexin A1 | 8 | 8 | 5,38 | 3,78 | 6,38 | 4,31 |
| P07355 | annexin A2 | 9 | 8 | 12,78 | 6,87 | 13,00 | 5,21 |
| P09525 | annexin A4 | 1 | 1 | 7,00 | 0,00 | 4,00 | 0,00 |
| P08758 | annexin A5 | 9 | 8 | 8,11 | 5,13 | 9,63 | 4,41 |
| P08133 | annexin A6 | 4 | 5 | 5,75 | 4,86 | 4,20 | 3,90 |
| P20073 | annexin A7 | 1 | 1 | 2,00 | 0,00 | 2,00 | 0,00 |
| Q8NCW5 | apolipoprotein A-I binding protein | 1 | 0 | 3,00 | 0,00 | 0,00 | 0,00 |
| P15289 | arylsulfatase A | 10 | 8 | 5,20 | 2,49 | 4,63 | 2,00 |
| P15848 | arylsulfatase B | 3 | 1 | 3,33 | 1,15 | 2,00 | 0,00 |
| P20933 | aspartylglucosaminidase | 3 | 3 | 4,67 | 2,52 | 4,33 | 2,08 |
| P53396 | ATP citrate lyase | 4 | 3 | 5,75 | 2,99 | 4,33 | 3,21 |
| Q15904 | ATPase, H+ transporting, lysosomal accessory protein 1 | 5 | 6 | 4,00 | 1,22 | 3,50 | 0,55 |
| O75787 | ATPase, H+ transporting, lysosomal accessory protein 2 | 5 | 2 | 4,40 | 3,36 | 4,50 | 2,12 |
| P30530 | AXL receptor tyrosine kinase | 7 | 7 | 5,57 | 1,40 | 4,57 | 2,57 |
| P35613 | basigin (Ok blood group) | 3 | 2 | 2,00 | 0,00 | 2,50 | 0,71 |
| Q07812 | BCL2-associated X protein | 1 | 1 | 4,00 | 0,00 | 2,00 | 0,00 |
| P61769 | beta-2-microglobulin | 10 | 9 | 18,50 | 8,32 | 24,11 | 11,72 |
| P21810 | biglycan | 10 | 9 | 27,90 | 17,87 | 18,67 | 8,15 |
| P30043 | biliverdin reductase B (flavin reductase (NADPH)) | 1 | 0 | 4,00 | 0,00 | 0,00 | 0,00 |
| P43251 | biotinidase | 5 | 5 | 2,60 | 0,89 | 2,60 | 0,89 |
| Q10588 | bone marrow stromal cell antigen 1 | 3 | 4 | 3,33 | 0,58 | 2,75 | 0,96 |
| P13497 | bone morphogenetic protein 1 | 10 | 9 | 4,00 | 2,58 | 4,67 | 2,40 |
| P80723 | brain abundant, membrane attached signal protein 1 | 5 | 4 | 6,00 | 5,70 | 7,75 | 4,92 |
| Q9Y240 | C-type lectin domain family 11, member A | 9 | 8 | 5,11 | 1,96 | 5,88 | 3,18 |
| P05452 | C-type lectin domain family 3, member B | 1 | 3 | 5,00 | 0,00 | 4,00 | 2,65 |
| Q9BXJ0 | C1q and tumor necrosis factor related protein 5 | 5 | 3 | 2,40 | 0,89 | 3,67 | 2,08 |
| P55287 | cadherin 11, type 2, OB-cadherin (osteoblast) | 4 | 4 | 4,25 | 3,20 | 3,00 | 2,00 |
| P55290 | cadherin 13, H-cadherin (heart) | 8 | 8 | 4,00 | 1,77 | 4,25 | 1,16 |
| P19022 | cadherin 2, type 1, N-cadherin (neuronal) | 10 | 9 | 8,00 | 4,50 | 8,33 | 2,78 |
| P27797 | calreticulin | 10 | 8 | 9,50 | 4,45 | 9,50 | 3,89 |
| O94985 | calsyntenin 1 | 10 | 8 | 13,60 | 4,90 | 14,25 | 2,96 |
| O43852 | calumenin | 10 | 9 | 19,30 | 8,73 | 25,00 | 10,51 |
| Q9Y2B0 | canopy 2 homolog (zebrafish) | 4 | 6 | 3,25 | 1,50 | 3,50 | 1,38 |
| Q8N129 | canopy 4 homolog (zebrafish) | 1 | 1 | 2,00 | 0,00 | 2,00 | 0,00 |
| Q01518 | CAP, adenylate cyclase-associated protein 1 (yeast) | 4 | 4 | 5,75 | 4,35 | 3,25 | 1,50 |
| P47756 | capping protein (actin filament) muscle Z-line, beta | 2 | 1 | 3,00 | 1,41 | 4,00 | 0,00 |
| P40121 | capping protein (actin filament), gelsolin-like | 1 | 1 | 14,00 | 0,00 | 7,00 | 0,00 |
| O43570 | carbonic anhydrase XII | 2 | 3 | 3,50 | 2,12 | 4,00 | 2,00 |
| P16152 | carbonyl reductase 1 | 1 | 1 | 7,00 | 0,00 | 3,00 | 0,00 |
| O75828 | carbonyl reductase 3 | 1 | 0 | 3,00 | 0,00 | 0,00 | 0,00 |
| Q9UI42 | carboxypeptidase A4 | 1 | 0 | 2,00 | 0,00 | 0,00 | 0,00 |
| P16870 | carboxypeptidase E | 10 | 8 | 4,80 | 2,04 | 7,38 | 3,96 |
| O75718 | cartilage associated protein | 4 | 0 | 2,50 | 1,00 | 0,00 | 0,00 |
| P49747 | cartilage oligomeric matrix protein | 8 | 9 | 41,13 | 43,11 | 30,00 | 26,58 |
| P21964 | catechol-O-methyltransferase | 1 | 0 | 3,00 | 0,00 | 0,00 | 0,00 |
| P10619 | cathepsin A | 9 | 8 | 7,11 | 4,04 | 6,88 | 3,31 |
| P07858 | cathepsin B | 10 | 9 | 21,30 | 10,53 | 22,33 | 13,36 |
| P53634 | cathepsin C | 1 | 0 | 2,00 | 0,00 | 0,00 | 0,00 |
| P07339 | cathepsin D | 9 | 8 | 15,44 | 8,29 | 15,00 | 7,93 |
| Q9UBX1 | cathepsin F | 7 | 4 | 3,43 | 1,99 | 4,75 | 2,50 |
| P43235 | cathepsin K | 5 | 5 | 5,80 | 5,76 | 5,60 | 3,58 |
| P07711 | cathepsin L1 | 9 | 8 | 9,67 | 6,42 | 10,13 | 6,42 |
| P25774 | cathepsin S | 3 | 3 | 2,33 | 0,58 | 3,67 | 2,08 |
| Q9UBR2 | cathepsin Z | 7 | 8 | 6,29 | 1,60 | 5,25 | 3,11 |
| Q6YHK3 | CD109 molecule | 10 | 7 | 11,60 | 10,21 | 12,43 | 8,44 |
| P08571 | CD14 molecule | 1 | 0 | 2,00 | 0,00 | 0,00 | 0,00 |
| Q9HCU0 | CD248 molecule, endosialin | 7 | 5 | 5,00 | 2,58 | 7,20 | 3,03 |
| P16070 | CD44 molecule (Indian blood group) | 4 | 7 | 2,00 | 0,00 | 2,43 | 1,13 |
| P08174 | CD55 molecule, decay accelerating factor for complement (Cromer blood group) | 2 | 2 | 2,50 | 0,71 | 2,50 | 0,71 |
| P13987 | CD59 molecule, complement regulatory protein | 2 | 3 | 2,00 | 0,00 | 2,00 | 0,00 |
| P60033 | CD81 molecule | 2 | 1 | 2,00 | 0,00 | 2,00 | 0,00 |
| P14209 | CD99 molecule | 1 | 0 | 19,00 | 0,00 | 0,00 | 0,00 |
| Q16543 | cell division cycle 37 homolog (S. cerevisiae) | 1 | 1 | 2,00 | 0,00 | 2,00 | 0,00 |
| Q99674 | cell growth regulator with EF-hand domain 1 | 2 | 2 | 2,50 | 0,71 | 2,00 | 0,00 |
| O75629 | cellular repressor of E1A-stimulated genes 1 | 3 | 4 | 6,33 | 4,51 | 4,50 | 0,58 |
| O75503 | ceroid-lipofuscinosis, neuronal 5 | 1 | 2 | 3,00 | 0,00 | 2,50 | 0,71 |
| P09341 | chemokine (C-X-C motif) ligand 1 (melanoma growth stimulating activity, alpha) | 3 | 3 | 5,67 | 1,53 | 5,67 | 1,15 |
| P19876 | chemokine (C-X-C motif) ligand 3 | 1 | 0 | 3,00 | 0,00 | 0,00 | 0,00 |
| P42830 | chemokine (C-X-C motif) ligand 5 | 2 | 2 | 3,00 | 1,41 | 3,00 | 1,41 |
| P36222 | chitinase 3-like 1 (cartilage glycoprotein-39) | 9 | 8 | 6,11 | 2,85 | 6,63 | 3,93 |
| Q9BWS9 | chitinase domain containing 1 | 1 | 2 | 4,00 | 0,00 | 2,50 | 0,71 |
| Q01459 | chitobiase, di-N-acetyl- | 2 | 3 | 2,50 | 0,71 | 2,33 | 0,58 |
| O00299 | chloride intracellular channel 1 | 3 | 2 | 3,67 | 1,15 | 3,50 | 2,12 |
| Q9Y696 | chloride intracellular channel 4 | 5 | 4 | 3,40 | 0,89 | 3,00 | 1,41 |
| Q6UVK1 | chondroitin sulfate proteoglycan 4 | 1 | 1 | 2,00 | 0,00 | 2,00 | 0,00 |
| Q9HB07 | chromosome 12 open reading frame 10 | 1 | 0 | 2,00 | 0,00 | 0,00 | 0,00 |
| Q969H8 | chromosome 19 open reading frame 10 | 4 | 5 | 2,50 | 0,58 | 2,40 | 0,55 |
| P10909 | clusterin | 10 | 9 | 11,30 | 7,30 | 11,44 | 7,83 |
| Q96KP4 | CNDP dipeptidase 2 (metallopeptidase M20 family) | 1 | 1 | 5,00 | 0,00 | 7,00 | 0,00 |
| Q14019 | coactosin-like 1 (Dictyostelium) | 3 | 4 | 2,33 | 0,58 | 2,50 | 0,58 |
| P00734 | coagulation factor II (thrombin) | 3 | 3 | 2,00 | 0,00 | 9,67 | 13,28 |
| P12259 | coagulation factor V (proaccelerin, labile factor) | 1 | 1 | 2,00 | 0,00 | 2,00 | 0,00 |
| Q76M96 | coiled-coil domain containing 80 | 9 | 8 | 10,11 | 4,96 | 8,75 | 4,56 |
| Q96CG8 | collagen triple helix repeat containing 1 | 4 | 5 | 4,00 | 0,82 | 5,80 | 2,28 |
| P02452 | collagen, type I, alpha 1 | 10 | 9 | 147,80 | 74,91 | 132,44 | 52,19 |
| P08123 | collagen, type I, alpha 2 | 10 | 9 | 166,90 | 52,68 | 161,56 | 57,21 |
| P02461 | collagen, type III, alpha 1 | 10 | 9 | 44,60 | 26,76 | 46,33 | 18,49 |
| P02462 | collagen, type IV, alpha 1 | 8 | 3 | 4,63 | 2,33 | 5,00 | 1,73 |
| P08572 | collagen, type IV, alpha 2 | 9 | 8 | 14,00 | 8,92 | 9,63 | 7,37 |
| P20908 | collagen, type V, alpha 1 | 10 | 9 | 27,30 | 21,02 | 28,22 | 16,52 |
| P05997 | collagen, type V, alpha 2 | 10 | 9 | 16,10 | 9,65 | 13,78 | 7,66 |
| P12109 | collagen, type VI, alpha 1 | 10 | 9 | 58,90 | 23,51 | 52,67 | 24,11 |
| P12110 | collagen, type VI, alpha 2 | 10 | 8 | 21,60 | 10,45 | 22,25 | 10,48 |
| P12111 | collagen, type VI, alpha 3 | 10 | 9 | 67,90 | 25,88 | 68,56 | 41,21 |
| Q02388 | collagen, type VII, alpha 1 | 2 | 1 | 5,00 | 0,00 | 4,00 | 0,00 |
| P27658 | collagen, type VIII, alpha 1 | 1 | 0 | 4,00 | 0,00 | 0,00 | 0,00 |
| P12107 | collagen, type XI, alpha 1 | 6 | 6 | 8,67 | 6,25 | 8,50 | 6,09 |
| Q99715 | collagen, type XII, alpha 1 | 10 | 9 | 98,70 | 49,49 | 82,22 | 46,31 |
| Q05707 | collagen, type XIV, alpha 1 | 4 | 2 | 10,75 | 12,04 | 18,50 | 9,19 |
| P39059 | collagen, type XV, alpha 1 | 6 | 5 | 9,00 | 5,22 | 7,40 | 6,31 |
| Q07092 | collagen, type XVI, alpha 1 | 3 | 3 | 6,00 | 3,00 | 5,00 | 2,65 |
| P39060 | collagen, type XVIII, alpha 1 | 2 | 1 | 2,50 | 0,71 | 3,00 | 0,00 |
| P09603 | colony stimulating factor 1 (macrophage) | 10 | 9 | 7,30 | 3,89 | 8,78 | 3,53 |
| P00736 | complement component 1, r subcomponent | 10 | 10 | 37,90 | 17,23 | 35,60 | 20,11 |
| P09871 | complement component 1, s subcomponent | 10 | 10 | 35,50 | 14,16 | 34,80 | 19,13 |
| P06681 | complement component 2 | 4 | 7 | 3,50 | 1,00 | 3,43 | 2,07 |
| P0C0L4 | complement component 4A (Rodgers blood group) | 1 | 2 | 2,00 | 0,00 | 34,50 | 44,55 |
| P0C0L5 | complement component 4B (Chido blood group) | 1 | 2 | 2,00 | 0,00 | 35,50 | 45,96 |
| P00751 | complement factor B | 8 | 8 | 8,13 | 5,94 | 13,13 | 14,11 |
| P00746 | complement factor D (adipsin) | 7 | 6 | 4,43 | 2,07 | 4,50 | 2,51 |
| P08603 | complement factor H | 5 | 4 | 4,60 | 3,65 | 7,50 | 4,20 |
| P29279 | connective tissue growth factor | 9 | 9 | 4,33 | 2,55 | 4,33 | 1,58 |
| O60888 | cutA divalent cation tolerance homolog (E. coli) | 3 | 3 | 4,33 | 2,08 | 3,67 | 2,08 |
| P01034 | cystatin C | 10 | 9 | 10,00 | 6,06 | 10,00 | 4,44 |
| P01037 | cystatin SN | 1 | 1 | 3,00 | 0,00 | 2,00 | 0,00 |
| P21291 | cysteine and glycine-rich protein 1 | 1 | 1 | 3,00 | 0,00 | 3,00 | 0,00 |
| O00622 | cysteine-rich, angiogenic inducer, 61 | 7 | 8 | 2,86 | 0,69 | 4,13 | 1,36 |
| P30085 | cytidine monophosphate (UMP-CMP) kinase 1, cytosolic | 3 | 3 | 3,00 | 0,00 | 2,33 | 0,58 |
| O75462 | cytokine receptor-like factor 1 | 4 | 3 | 2,50 | 0,58 | 4,00 | 1,00 |
| Q16531 | damage-specific DNA binding protein 1, 127kDa | 1 | 0 | 2,00 | 0,00 | 0,00 | 0,00 |
| P07585 | decorin | 10 | 9 | 26,20 | 16,77 | 22,44 | 12,31 |
| O00115 | deoxyribonuclease II, lysosomal | 4 | 4 | 3,75 | 1,50 | 3,25 | 0,50 |
| P33316 | deoxyuridine triphosphatase | 1 | 1 | 3,00 | 0,00 | 2,00 | 0,00 |
| Q07507 | dermatopontin | 3 | 3 | 5,00 | 3,00 | 4,67 | 2,52 |
| O94907 | dickkopf homolog 1 (Xenopus laevis) | 9 | 8 | 3,33 | 1,41 | 4,13 | 1,13 |
| Q9UBP4 | dickkopf homolog 3 (Xenopus laevis) | 10 | 9 | 7,60 | 3,84 | 9,78 | 4,12 |
| O94760 | dimethylarginine dimethylaminohydrolase 1 | 1 | 1 | 5,00 | 0,00 | 5,00 | 0,00 |
| Q9NY33 | dipeptidyl-peptidase 3 | 1 | 1 | 4,00 | 0,00 | 3,00 | 0,00 |
| P27487 | dipeptidyl-peptidase 4 | 1 | 1 | 4,00 | 0,00 | 3,00 | 0,00 |
| Q9UHL4 | dipeptidyl-peptidase 7 | 5 | 4 | 5,60 | 4,51 | 7,00 | 4,83 |
| Q96PD2 | discoidin, CUB and LCCL domain containing 2 | 1 | 1 | 2,00 | 0,00 | 2,00 | 0,00 |
| Q9UBS4 | DnaJ (Hsp40) homolog, subfamily B, member 11 | 1 | 2 | 2,00 | 0,00 | 2,50 | 0,71 |
| Q13217 | DnaJ (Hsp40) homolog, subfamily C, member 3 | 2 | 2 | 2,00 | 0,00 | 2,50 | 0,71 |
| Q9UJU6 | drebrin-like | 1 | 1 | 3,00 | 0,00 | 2,00 | 0,00 |
| Q13561 | dynactin 2 (p50) | 2 | 1 | 4,50 | 3,54 | 4,00 | 0,00 |
| Q14118 | dystroglycan 1 (dystrophin-associated glycoprotein 1) | 8 | 9 | 4,75 | 1,67 | 5,33 | 3,00 |
| Q15075 | early endosome antigen 1 | 1 | 1 | 12,00 | 0,00 | 5,00 | 0,00 |
| Q13822 | ectonucleotide pyrophosphatase/phosphodiesterase 2 | 10 | 8 | 18,50 | 11,02 | 20,88 | 10,66 |
| Q12805 | EGF-containing fibulin-like extracellular matrix protein 1 | 10 | 9 | 27,70 | 15,43 | 28,67 | 13,58 |
| O95967 | EGF-containing fibulin-like extracellular matrix protein 2 | 10 | 8 | 6,60 | 3,10 | 8,63 | 2,26 |
| P15502 | elastin | 7 | 7 | 16,86 | 16,17 | 21,86 | 23,97 |
| Q9Y6C2 | elastin microfibril interfacer 1 | 9 | 7 | 5,78 | 3,07 | 5,71 | 2,43 |
| Q9BXX0 | elastin microfibril interfacer 2 | 5 | 3 | 4,40 | 1,82 | 4,67 | 1,53 |
| P17813 | endoglin | 4 | 6 | 2,75 | 0,96 | 3,33 | 1,51 |
| Q9NZ08 | endoplasmic reticulum aminopeptidase 1 | 1 | 0 | 2,00 | 0,00 | 0,00 | 0,00 |
| P30040 | endoplasmic reticulum protein 29 | 6 | 6 | 2,50 | 0,84 | 2,83 | 0,98 |
| Q9BS26 | endoplasmic reticulum protein 44 | 2 | 1 | 2,00 | 0,00 | 2,00 | 0,00 |
| Q9NQ30 | endothelial cell-specific molecule 1 | 1 | 1 | 2,00 | 0,00 | 2,00 | 0,00 |
| Q9UM22 | ependymin related protein 1 (zebrafish) | 5 | 7 | 2,80 | 0,84 | 2,14 | 0,38 |
| P00533 | epidermal growth factor receptor (erythroblastic leukemia viral (v-erb-b) oncogene homolog, avian) | 1 | 1 | 2,00 | 0,00 | 2,00 | 0,00 |
| Q12929 | epidermal growth factor receptor pathway substrate 8 | 1 | 1 | 4,00 | 0,00 | 2,00 | 0,00 |
| Q96HE7 | ERO1-like (S. cerevisiae) | 1 | 1 | 2,00 | 0,00 | 3,00 | 0,00 |
| Q93063 | exostoses (multiple) 2 | 5 | 5 | 3,60 | 2,19 | 3,60 | 1,14 |
| Q16610 | extracellular matrix protein 1 | 10 | 9 | 19,30 | 7,26 | 20,78 | 10,05 |
| O94769 | extracellular matrix protein 2, female organ and adipocyte specific | 1 | 0 | 2,00 | 0,00 | 0,00 | 0,00 |
| O75063 | family with sequence similarity 20, member B | 1 | 0 | 2,00 | 0,00 | 0,00 | 0,00 |
| Q8IXL6 | family with sequence similarity 20, member C | 1 | 1 | 2,00 | 0,00 | 2,00 | 0,00 |
| Q92520 | family with sequence similarity 3, member C | 6 | 6 | 4,17 | 2,48 | 4,50 | 2,35 |
| Q16658 | fascin homolog 1, actin-bundling protein (Strongylocentrotus purpuratus) | 4 | 2 | 3,50 | 1,91 | 4,00 | 1,41 |
| P49327 | fatty acid synthase | 2 | 2 | 7,00 | 4,24 | 3,00 | 1,41 |
| P35555 | fibrillin 1 | 10 | 9 | 102,20 | 50,21 | 105,33 | 33,06 |
| P35556 | fibrillin 2 | 7 | 7 | 19,43 | 18,66 | 15,71 | 15,48 |
| P11362 | fibroblast growth factor receptor 1 | 1 | 4 | 2,00 | 0,00 | 2,00 | 0,00 |
| Q06828 | fibromodulin | 7 | 7 | 6,29 | 3,68 | 5,43 | 2,23 |
| P02751 | fibronectin 1 | 10 | 9 | 301,40 | 129,64 | 261,44 | 144,49 |
| Q4ZHG4 | fibronectin type III domain containing 1 | 10 | 8 | 9,30 | 6,53 | 11,13 | 7,94 |
| P23142 | fibulin 1 | 10 | 9 | 24,80 | 11,85 | 22,89 | 11,53 |
| P98095 | fibulin 2 | 10 | 9 | 26,40 | 14,61 | 25,00 | 10,32 |
| Q9UBX5 | fibulin 5 | 10 | 8 | 6,00 | 4,40 | 7,13 | 4,16 |
| P21333 | filamin A, alpha (actin binding protein 280) | 10 | 9 | 38,50 | 20,48 | 41,78 | 23,19 |
| O75369 | filamin B, beta (actin binding protein 278) | 8 | 7 | 13,88 | 12,55 | 14,00 | 11,28 |
| Q14315 | filamin C, gamma (actin binding protein 280) | 9 | 9 | 17,22 | 12,51 | 16,22 | 11,16 |
| Q8TAL6 | fin bud initiation factor homolog (zebrafish) | 1 | 0 | 2,00 | 0,00 | 0,00 | 0,00 |
| Q96AY3 | FK506 binding protein 10, 65 kDa | 6 | 4 | 6,00 | 4,20 | 5,75 | 1,50 |
| P26885 | FK506 binding protein 2, 13kDa | 2 | 3 | 3,00 | 1,41 | 2,00 | 0,00 |
| Q02790 | FK506 binding protein 4, 59kDa | 1 | 0 | 2,00 | 0,00 | 0,00 | 0,00 |
| Q9Y680 | FK506 binding protein 7 | 2 | 2 | 3,00 | 1,41 | 3,00 | 1,41 |
| O95302 | FK506 binding protein 9, 63 kDa | 6 | 8 | 6,00 | 1,67 | 4,00 | 2,27 |
| P19883 | follistatin | 7 | 6 | 3,57 | 1,27 | 4,50 | 2,07 |
| Q12841 | follistatin-like 1 | 10 | 9 | 18,10 | 9,87 | 22,11 | 6,13 |
| O95633 | follistatin-like 3 (secreted glycoprotein) | 5 | 6 | 2,40 | 0,55 | 2,83 | 1,33 |
| P04066 | fucosidase, alpha-L- 1, tissue | 3 | 2 | 2,67 | 0,58 | 2,00 | 0,00 |
| Q9BTY2 | fucosidase, alpha-L- 2, plasma | 3 | 4 | 4,00 | 1,00 | 3,25 | 0,96 |
| P35637 | fusion (involved in t(12;16) in malignant liposarcoma) | 1 | 1 | 3,00 | 0,00 | 2,00 | 0,00 |
| P34059 | galactosamine (N-acetyl)-6-sulfate sulfatase | 7 | 8 | 4,29 | 0,76 | 3,63 | 1,51 |
| P16278 | galactosidase, beta 1 | 2 | 3 | 6,50 | 0,71 | 3,67 | 0,58 |
| P54803 | galactosylceramidase | 1 | 0 | 2,00 | 0,00 | 0,00 | 0,00 |
| Q92820 | gamma-glutamyl hydrolase (conjugase, folylpolygammaglutamyl hydrolase) | 5 | 5 | 4,40 | 1,34 | 4,80 | 1,79 |
| P06396 | gelsolin (amyloidosis, Finnish type) | 10 | 9 | 13,20 | 9,26 | 17,78 | 13,36 |
| P15586 | glucosamine (N-acetyl)-6-sulfatase | 9 | 8 | 8,89 | 5,93 | 9,75 | 6,45 |
| P11413 | glucose-6-phosphate dehydrogenase | 1 | 1 | 2,00 | 0,00 | 2,00 | 0,00 |
| P10253 | glucosidase, alpha; acid | 3 | 3 | 3,33 | 2,31 | 4,33 | 2,52 |
| Q14697 | glucosidase, alpha; neutral AB | 1 | 1 | 3,00 | 0,00 | 3,00 | 0,00 |
| P04062 | glucosidase, beta; acid (includes glucosylceramidase) | 2 | 2 | 4,00 | 1,41 | 3,00 | 1,41 |
| Q16769 | glutaminyl-peptide cyclotransferase | 4 | 4 | 2,25 | 0,50 | 4,00 | 1,41 |
| P78417 | glutathione S-transferase omega 1 | 1 | 1 | 6,00 | 0,00 | 5,00 | 0,00 |
| P09211 | glutathione S-transferase pi 1 | 7 | 7 | 5,43 | 3,36 | 5,14 | 2,04 |
| Q14956 | glycoprotein (transmembrane) nmb | 4 | 4 | 4,75 | 1,89 | 4,00 | 2,16 |
| P41250 | glycyl-tRNA synthetase | 4 | 3 | 2,50 | 1,00 | 2,67 | 0,58 |
| P35052 | glypican 1 | 8 | 4 | 5,50 | 2,73 | 6,25 | 5,97 |
| Q9Y625 | glypican 6 | 2 | 2 | 5,50 | 3,54 | 4,50 | 3,54 |
| P17900 | GM2 ganglioside activator | 2 | 2 | 3,50 | 2,12 | 4,00 | 0,00 |
| Q92896 | golgi apparatus protein 1 | 1 | 1 | 2,00 | 0,00 | 2,00 | 0,00 |
| Q8NBJ4 | golgi membrane protein 1 | 7 | 6 | 4,86 | 1,46 | 6,00 | 2,00 |
| P28799 | granulin | 8 | 8 | 7,50 | 5,13 | 8,75 | 5,44 |
| O60565 | gremlin 1, cysteine knot superfamily, homolog (Xenopus laevis) | 2 | 3 | 3,00 | 1,41 | 2,33 | 0,58 |
| Q9H772 | gremlin 2, cysteine knot superfamily, homolog (Xenopus laevis) | 3 | 3 | 3,00 | 1,00 | 2,33 | 0,58 |
| P54826 | growth arrest-specific 1 | 1 | 0 | 2,00 | 0,00 | 0,00 | 0,00 |
| Q99988 | growth differentiation factor 15 | 4 | 3 | 2,00 | 0,00 | 2,00 | 0,00 |
| P04792 | heat shock 27kDa protein 1 | 7 | 8 | 4,29 | 1,80 | 4,13 | 1,64 |
| P48723 | heat shock protein 70kDa family, member 13 | 7 | 5 | 4,57 | 2,23 | 5,00 | 1,22 |
| P14625 | heat shock protein 90kDa beta (Grp94), member 1 | 9 | 8 | 10,00 | 3,87 | 9,75 | 2,31 |
| Q9NRV9 | heme binding protein 1 | 1 | 1 | 4,00 | 0,00 | 3,00 | 0,00 |
| Q9Y5Z4 | heme binding protein 2 | 3 | 6 | 2,00 | 0,00 | 2,50 | 0,84 |
| Q96RW7 | hemicentin 1 | 4 | 3 | 7,50 | 7,55 | 7,33 | 6,81 |
| P68871 | hemoglobin, beta | 2 | 1 | 2,00 | 0,00 | 2,00 | 0,00 |
| P98160 | heparan sulfate proteoglycan 2 | 10 | 9 | 73,40 | 39,95 | 58,78 | 40,99 |
| O14979 | heterogeneous nuclear ribonucleoprotein D-like | 2 | 2 | 4,50 | 3,54 | 3,00 | 1,41 |
| P06865 | hexosaminidase A (alpha polypeptide) | 9 | 7 | 8,78 | 6,76 | 10,00 | 7,26 |
| P07686 | hexosaminidase B (beta polypeptide) | 10 | 7 | 7,80 | 4,10 | 8,29 | 3,04 |
| O95479 | hexose-6-phosphate dehydrogenase | 1 | 0 | 3,00 | 0,00 | 0,00 | 0,00 |
| P16188 | HLA class I histocompatibility antigen, A-30 alpha chain | 2 | 2 | 6,00 | 5,66 | 5,50 | 0,71 |
| P16190 | HLA class I histocompatibility antigen, A-33 alpha chain | 1 | 1 | 2,00 | 0,00 | 6,00 | 0,00 |
| P30459 | HLA class I histocompatibility antigen, A-74 alpha chain | 1 | 1 | 2,00 | 0,00 | 5,00 | 0,00 |
| Q07000 | HLA class I histocompatibility antigen, Cw-15 alpha chain | 1 | 1 | 2,00 | 0,00 | 4,00 | 0,00 |
| Q29865 | HLA class I histocompatibility antigen, Cw-18 alpha chain | 2 | 1 | 5,50 | 0,71 | 3,00 | 0,00 |
| Q92743 | HtrA serine peptidase 1 | 9 | 7 | 11,00 | 4,90 | 9,57 | 4,86 |
| P83110 | HtrA serine peptidase 3 | 1 | 2 | 3,00 | 0,00 | 4,50 | 0,71 |
| P10915 | hyaluronan and proteoglycan link protein 1 | 4 | 3 | 3,75 | 0,96 | 3,33 | 1,53 |
| Q96S86 | hyaluronan and proteoglycan link protein 3 | 1 | 0 | 2,00 | 0,00 | 0,00 | 0,00 |
| P11021 | hypothetical gene supported by AF216292; NM_005347; heat shock 70kDa protein 5 (glucose-regulated protein, 78kDa) | 10 | 9 | 18,10 | 7,58 | 18,33 | 4,36 |
| O60784 | hypothetical LOC100128526; target of myb1 (chicken) | 1 | 2 | 9,00 | 0,00 | 4,00 | 0,00 |
| P02649 | hypothetical LOC100129500; apolipoprotein E | 1 | 2 | 2,00 | 0,00 | 3,00 | 1,41 |
| Q13740 | hypothetical protein LOC100133690; activated leukocyte cell adhesion molecule | 3 | 5 | 7,00 | 2,65 | 4,20 | 4,38 |
| Q96QK1 | hypothetical protein LOC100133770; vacuolar protein sorting 35 homolog (S. cerevisiae) | 1 | 0 | 2,00 | 0,00 | 0,00 | 0,00 |
| Q9Y4L1 | hypoxia up-regulated 1 | 3 | 4 | 2,33 | 0,58 | 2,75 | 0,96 |
| P22304 | iduronate 2-sulfatase | 2 | 2 | 3,50 | 2,12 | 3,00 | 1,41 |
| P35475 | iduronidase, alpha-L- | 3 | 2 | 3,33 | 1,15 | 3,00 | 0,00 |
| O14498 | immunoglobulin superfamily containing leucine-rich repeat | 10 | 8 | 12,60 | 8,09 | 11,25 | 5,20 |
| P08476 | inhibin, beta A | 8 | 5 | 7,00 | 5,48 | 7,20 | 3,56 |
| P17936 | insulin-like growth factor binding protein 3 | 10 | 9 | 11,20 | 3,97 | 13,11 | 1,69 |
| P22692 | insulin-like growth factor binding protein 4 | 9 | 9 | 8,00 | 3,84 | 8,11 | 3,55 |
| P24593 | insulin-like growth factor binding protein 5 | 8 | 8 | 5,38 | 1,77 | 7,13 | 3,23 |
| P24592 | insulin-like growth factor binding protein 6 | 9 | 9 | 10,22 | 4,92 | 10,67 | 5,27 |
| Q16270 | insulin-like growth factor binding protein 7 | 10 | 9 | 15,60 | 5,38 | 15,11 | 3,02 |
| Q9Y287 | integral membrane protein 2B | 3 | 3 | 3,33 | 1,53 | 2,67 | 1,15 |
| P06756 | integrin, alpha V (vitronectin receptor, alpha polypeptide, antigen CD51) | 1 | 0 | 3,00 | 0,00 | 0,00 | 0,00 |
| P05556 | integrin, beta 1 (fibronectin receptor, beta polypeptide, antigen CD29 includes MDF2, MSK12) | 3 | 1 | 2,00 | 0,00 | 3,00 | 0,00 |
| O95965 | integrin, beta-like 1 (with EGF-like repeat domains) | 9 | 8 | 6,33 | 3,50 | 6,88 | 3,60 |
| P19823 | inter-alpha (globulin) inhibitor H2 | 3 | 7 | 3,33 | 0,58 | 3,14 | 0,90 |
| Q06033 | inter-alpha (globulin) inhibitor H3 | 3 | 5 | 2,67 | 0,58 | 2,60 | 0,89 |
| Q86UX2 | inter-alpha (globulin) inhibitor H5 | 1 | 0 | 3,00 | 0,00 | 0,00 | 0,00 |
| P05362 | intercellular adhesion molecule 1 | 1 | 4 | 2,00 | 0,00 | 3,50 | 1,29 |
| P13284 | interferon, gamma-inducible protein 30 | 3 | 4 | 7,00 | 2,65 | 5,50 | 2,08 |
| P05231 | interleukin 6 (interferon, beta 2) | 6 | 6 | 3,33 | 1,86 | 3,67 | 2,73 |
| P10145 | interleukin 8 | 2 | 3 | 2,50 | 0,71 | 3,33 | 0,58 |
| Q7Z4H8 | KDEL (Lys-Asp-Glu-Leu) containing 2 | 1 | 0 | 3,00 | 0,00 | 0,00 | 0,00 |
| Q8WUJ3 | KIAA1199 | 1 | 1 | 2,00 | 0,00 | 6,00 | 0,00 |
| P21583 | KIT ligand | 2 | 2 | 2,00 | 0,00 | 2,00 | 0,00 |
| P00338 | lactate dehydrogenase A | 9 | 8 | 11,00 | 5,89 | 9,88 | 3,52 |
| P07195 | lactate dehydrogenase B | 7 | 7 | 4,71 | 1,60 | 4,14 | 2,73 |
| P02788 | lactotransferrin | 1 | 1 | 2,00 | 0,00 | 3,00 | 0,00 |
| P02545 | lamin A/C | 9 | 9 | 11,89 | 6,07 | 11,22 | 6,63 |
| P25391 | laminin, alpha 1 | 3 | 4 | 11,00 | 7,00 | 10,25 | 10,05 |
| P24043 | laminin, alpha 2 | 9 | 8 | 11,22 | 7,64 | 11,00 | 8,40 |
| Q16363 | laminin, alpha 4 | 10 | 9 | 33,70 | 12,36 | 33,11 | 15,73 |
| O15230 | laminin, alpha 5 | 1 | 1 | 3,00 | 0,00 | 3,00 | 0,00 |
| P07942 | laminin, beta 1 | 10 | 9 | 30,00 | 14,38 | 29,00 | 12,99 |
| P55268 | laminin, beta 2 (laminin S) | 10 | 8 | 18,90 | 8,53 | 21,00 | 9,30 |
| P11047 | laminin, gamma 1 (formerly LAMB2) | 10 | 9 | 35,10 | 11,13 | 37,56 | 15,26 |
| Q14766 | latent transforming growth factor beta binding protein 1 | 3 | 5 | 12,67 | 8,62 | 7,40 | 6,43 |
| Q14767 | latent transforming growth factor beta binding protein 2 | 10 | 9 | 25,60 | 9,34 | 28,89 | 10,78 |
| Q6UX15 | layilin | 2 | 2 | 2,00 | 0,00 | 2,00 | 0,00 |
| Q08380 | lectin, galactoside-binding, soluble, 3 binding protein | 10 | 9 | 20,80 | 6,30 | 21,11 | 8,80 |
| Q12907 | lectin, mannose-binding 2 | 3 | 4 | 4,00 | 2,65 | 3,00 | 1,41 |
| Q99538 | legumain | 9 | 8 | 7,56 | 4,67 | 8,00 | 3,25 |
| Q8TF66 | leucine rich repeat containing 15 | 1 | 0 | 2,00 | 0,00 | 0,00 | 0,00 |
| Q14392 | leucine rich repeat containing 32 | 1 | 2 | 5,00 | 0,00 | 2,50 | 0,71 |
| Q9UHB6 | LIM domain and actin binding 1 | 1 | 2 | 3,00 | 0,00 | 2,50 | 0,71 |
| P38571 | lipase A, lysosomal acid, cholesterol esterase | 2 | 1 | 3,00 | 1,41 | 3,00 | 0,00 |
| P01130 | low density lipoprotein receptor | 1 | 1 | 4,00 | 0,00 | 7,00 | 0,00 |
| Q07954 | low density lipoprotein-related protein 1 (alpha-2-macroglobulin receptor) | 8 | 8 | 9,75 | 6,92 | 11,38 | 9,12 |
| P51884 | lumican | 10 | 9 | 29,40 | 16,63 | 29,67 | 18,71 |
| P11279 | lysosomal-associated membrane protein 1 | 4 | 5 | 3,25 | 0,96 | 3,60 | 0,55 |
| P13473 | lysosomal-associated membrane protein 2 | 6 | 6 | 2,50 | 0,84 | 2,17 | 0,41 |
| P28300 | lysyl oxidase | 10 | 9 | 7,80 | 3,19 | 8,67 | 5,43 |
| Q08397 | lysyl oxidase-like 1 | 8 | 5 | 3,38 | 1,51 | 4,20 | 2,49 |
| Q9Y4K0 | lysyl oxidase-like 2 | 9 | 7 | 8,56 | 5,08 | 9,86 | 6,09 |
| P14174 | macrophage migration inhibitory factor (glycosylation-inhibiting factor) | 4 | 3 | 5,00 | 5,35 | 4,67 | 2,89 |
| P01891 | major histocompatibility complex, class I, A | 1 | 1 | 2,00 | 0,00 | 5,00 | 0,00 |
| P01892 | major histocompatibility complex, class I, A | 1 | 1 | 2,00 | 0,00 | 4,00 | 0,00 |
| P04439 | major histocompatibility complex, class I, A | 3 | 1 | 5,33 | 4,16 | 5,00 | 0,00 |
| P05534 | major histocompatibility complex, class I, A | 2 | 2 | 2,50 | 0,71 | 3,50 | 2,12 |
| P10314 | major histocompatibility complex, class I, A | 1 | 1 | 2,00 | 0,00 | 5,00 | 0,00 |
| P13746 | major histocompatibility complex, class I, A | 1 | 0 | 2,00 | 0,00 | 0,00 | 0,00 |
| P16189 | major histocompatibility complex, class I, A | 1 | 2 | 2,00 | 0,00 | 4,50 | 0,71 |
| P18462 | major histocompatibility complex, class I, A | 1 | 0 | 2,00 | 0,00 | 0,00 | 0,00 |
| P30443 | major histocompatibility complex, class I, A | 1 | 0 | 2,00 | 0,00 | 0,00 | 0,00 |
| P30447 | major histocompatibility complex, class I, A | 2 | 2 | 2,50 | 0,71 | 3,50 | 2,12 |
| P30450 | major histocompatibility complex, class I, A | 1 | 0 | 2,00 | 0,00 | 0,00 | 0,00 |
| P30453 | major histocompatibility complex, class I, A | 1 | 1 | 2,00 | 0,00 | 6,00 | 0,00 |
| P30455 | major histocompatibility complex, class I, A | 1 | 1 | 2,00 | 0,00 | 4,00 | 0,00 |
| P30456 | major histocompatibility complex, class I, A | 1 | 0 | 2,00 | 0,00 | 0,00 | 0,00 |
| P30457 | major histocompatibility complex, class I, A | 1 | 0 | 2,00 | 0,00 | 0,00 | 0,00 |
| P30512 | major histocompatibility complex, class I, A | 1 | 1 | 2,00 | 0,00 | 5,00 | 0,00 |
| P04222 | major histocompatibility complex, class I, C; major histocompatibility complex, class I, B | 1 | 1 | 2,00 | 0,00 | 5,00 | 0,00 |
| P10321 | major histocompatibility complex, class I, C; major histocompatibility complex, class I, B | 1 | 1 | 5,00 | 0,00 | 3,00 | 0,00 |
| P18465 | major histocompatibility complex, class I, C; major histocompatibility complex, class I, B | 1 | 0 | 3,00 | 0,00 | 0,00 | 0,00 |
| P30501 | major histocompatibility complex, class I, C; major histocompatibility complex, class I, B | 1 | 0 | 2,00 | 0,00 | 0,00 | 0,00 |
| P30504 | major histocompatibility complex, class I, C; major histocompatibility complex, class I, B | 1 | 1 | 2,00 | 0,00 | 4,00 | 0,00 |
| P30508 | major histocompatibility complex, class I, C; major histocompatibility complex, class I, B | 2 | 2 | 4,00 | 3,46 | 5,00 | 1,41 |
| P30510 | major histocompatibility complex, class I, C; major histocompatibility complex, class I, B | 1 | 1 | 2,00 | 0,00 | 4,00 | 0,00 |
| Q29960 | major histocompatibility complex, class I, C; major histocompatibility complex, class I, B | 1 | 1 | 2,00 | 0,00 | 3,00 | 0,00 |
| Q31612 | major histocompatibility complex, class I, C; major histocompatibility complex, class I, B | 2 | 3 | 7,00 | 1,41 | 6,67 | 0,58 |
| Q95604 | major histocompatibility complex, class I, C; major histocompatibility complex, class I, B | 1 | 2 | 2,00 | 0,00 | 5,00 | 1,41 |
| Q14764 | major vault protein | 4 | 2 | 8,25 | 8,66 | 9,00 | 8,49 |
| P48740 | mannan-binding lectin serine peptidase 1 (C4/C2 activating component of Ra-reactive factor) | 2 | 2 | 5,00 | 1,41 | 5,00 | 2,83 |
| Q9UBG0 | mannose receptor, C type 2 | 3 | 4 | 7,33 | 5,03 | 5,25 | 2,63 |
| O00754 | mannosidase, alpha, class 2B, member 1 | 5 | 5 | 4,40 | 3,29 | 4,60 | 2,07 |
| Q9Y2E5 | mannosidase, alpha, class 2B, member 2 | 4 | 4 | 5,00 | 3,16 | 4,00 | 2,45 |
| O00462 | mannosidase, beta A, lysosomal | 8 | 7 | 6,50 | 4,66 | 7,00 | 3,46 |
| O15232 | matrilin 3 | 1 | 0 | 3,00 | 0,00 | 0,00 | 0,00 |
| P03956 | matrix metallopeptidase 1 (interstitial collagenase) | 10 | 7 | 14,60 | 12,10 | 16,57 | 12,55 |
| P50281 | matrix metallopeptidase 14 (membrane-inserted) | 5 | 5 | 3,80 | 1,79 | 4,80 | 3,03 |
| Q99542 | matrix metallopeptidase 19 | 1 | 0 | 2,00 | 0,00 | 0,00 | 0,00 |
| P08253 | matrix metallopeptidase 2 (gelatinase A, 72kDa gelatinase, 72kDa type IV collagenase) | 10 | 9 | 69,10 | 21,65 | 60,78 | 25,02 |
| P08254 | matrix metallopeptidase 3 (stromelysin 1, progelatinase) | 10 | 5 | 12,60 | 13,37 | 14,40 | 8,47 |
| P14780 | matrix metallopeptidase 9 (gelatinase B, 92kDa gelatinase, 92kDa type IV collagenase) | 1 | 1 | 9,00 | 0,00 | 13,00 | 0,00 |
| Q9NR99 | matrix-remodelling associated 5 | 6 | 3 | 5,83 | 7,00 | 7,33 | 8,39 |
| Q9BRK3 | matrix-remodelling associated 8 | 4 | 5 | 4,25 | 1,71 | 3,20 | 0,84 |
| Q5JRA6 | melanoma inhibitory activity family, member 3 | 1 | 2 | 5,00 | 0,00 | 3,00 | 1,41 |
| Q9BXJ0 | membrane frizzled-related protein | 5 | 3 | 2,40 | 0,89 | 3,67 | 2,08 |
| P55145 | mesencephalic astrocyte-derived neurotrophic factor | 4 | 5 | 3,50 | 1,29 | 3,00 | 0,71 |
| Q641Q3 | meteorin, glial cell differentiation regulator-like; similar to meteorin, glial cell differentiation regulator-like | 2 | 2 | 2,50 | 0,71 | 2,00 | 0,00 |
| Q13361 | microfibrillar associated protein 5 | 7 | 7 | 4,71 | 3,15 | 5,14 | 2,79 |
| P55001 | microfibrillar-associated protein 2 | 1 | 4 | 2,00 | 0,00 | 2,00 | 0,00 |
| P55083 | microfibrillar-associated protein 4 | 2 | 4 | 6,00 | 5,66 | 4,75 | 3,59 |
| P78559 | microtubule-associated protein 1A | 1 | 3 | 7,00 | 0,00 | 5,33 | 4,04 |
| Q08431 | milk fat globule-EGF factor 8 protein | 7 | 7 | 6,57 | 2,57 | 5,71 | 3,25 |
| Q6UVY6 | monooxygenase, DBH-like 1 | 1 | 1 | 2,00 | 0,00 | 2,00 | 0,00 |
| Q8NI22 | multiple coagulation factor deficiency 2 | 5 | 5 | 5,60 | 1,52 | 5,60 | 2,30 |
| O75095 | multiple EGF-like-domains 6 | 4 | 3 | 5,25 | 3,40 | 7,67 | 3,51 |
| Q9UNW1 | multiple inositol polyphosphate histidine phosphatase, 1 | 1 | 1 | 2,00 | 0,00 | 3,00 | 0,00 |
| P35580 | myosin, heavy chain 10, non-muscle | 3 | 1 | 3,67 | 2,89 | 2,00 | 0,00 |
| P35579 | myosin, heavy chain 9, non-muscle | 10 | 9 | 13,20 | 9,14 | 15,11 | 12,41 |
| P60660 | myosin, light chain 6, alkali, smooth muscle and non-muscle | 9 | 9 | 4,89 | 2,47 | 5,67 | 2,12 |
| P17050 | N-acetylgalactosaminidase, alpha- | 2 | 1 | 3,50 | 0,71 | 3,00 | 0,00 |
| Q9UJ70 | N-acetylglucosamine kinase | 1 | 0 | 3,00 | 0,00 | 0,00 | 0,00 |
| Q9UJJ9 | N-acetylglucosamine-1-phosphate transferase, gamma subunit | 7 | 3 | 3,29 | 1,89 | 4,67 | 0,58 |
| P54802 | N-acetylglucosaminidase, alpha- | 7 | 7 | 10,29 | 5,94 | 11,29 | 3,55 |
| Q13510 | N-acylsphingosine amidohydrolase (acid ceramidase) 1 | 4 | 5 | 4,75 | 2,75 | 3,00 | 1,73 |
| Q92597 | N-myc downstream regulated 1 | 1 | 1 | 3,00 | 0,00 | 4,00 | 0,00 |
| P51688 | N-sulfoglucosamine sulfohydrolase | 3 | 5 | 5,33 | 2,52 | 3,40 | 0,55 |
| Q13765 | nascent polypeptide-associated complex alpha subunit | 4 | 5 | 2,50 | 0,58 | 3,40 | 1,14 |
| Q92859 | neogenin homolog 1 (chicken) | 2 | 1 | 2,00 | 0,00 | 2,00 | 0,00 |
| P48745 | nephroblastoma overexpressed gene | 4 | 3 | 4,00 | 2,83 | 4,00 | 1,00 |
| Q15843 | neural precursor cell expressed, developmentally down-regulated 8; similar to neural precursor cell expressed, developmentally down-regulated gene 8 | 1 | 1 | 2,00 | 0,00 | 2,00 | 0,00 |
| P41271 | neuroblastoma, suppression of tumorigenicity 1 | 1 | 2 | 2,00 | 0,00 | 2,00 | 0,00 |
| O94856 | neurofascin homolog (chicken) | 2 | 1 | 2,00 | 0,00 | 2,00 | 0,00 |
| Q9UMX5 | neuron derived neurotrophic factor | 1 | 3 | 4,00 | 0,00 | 2,33 | 0,58 |
| Q7Z3B1 | neuronal growth regulator 1 | 1 | 1 | 5,00 | 0,00 | 5,00 | 0,00 |
| O14786 | neuropilin 1 | 9 | 7 | 6,67 | 5,45 | 7,14 | 4,14 |
| O60462 | neuropilin 2 | 1 | 0 | 2,00 | 0,00 | 0,00 | 0,00 |
| P43490 | nicotinamide phosphoribosyltransferase | 1 | 1 | 3,00 | 0,00 | 3,00 | 0,00 |
| P14543 | nidogen 1 | 10 | 8 | 15,40 | 9,18 | 16,13 | 6,64 |
| Q14112 | nidogen 2 (osteonidogen) | 7 | 5 | 9,86 | 7,01 | 9,20 | 8,01 |
| P61916 | Niemann-Pick disease, type C2 | 8 | 7 | 6,50 | 3,51 | 7,29 | 3,04 |
| Q15233 | non-POU domain containing, octamer-binding | 2 | 2 | 5,00 | 4,24 | 3,50 | 2,12 |
| Q04721 | Notch homolog 2 (Drosophila) | 1 | 0 | 3,00 | 0,00 | 0,00 | 0,00 |
| Q02818 | nucleobindin 1 | 10 | 9 | 18,00 | 5,25 | 20,56 | 4,33 |
| P80303 | nucleobindin 2 | 10 | 9 | 6,30 | 2,58 | 7,67 | 2,55 |
| Q9UKK9 | nudix (nucleoside diphosphate linked moiety X)-type motif 5 | 1 | 2 | 6,00 | 0,00 | 2,00 | 0,00 |
| Q86UD1 | OAF homolog (Drosophila) | 3 | 5 | 2,00 | 0,00 | 2,40 | 0,89 |
| Q6UWY5 | olfactomedin-like 1 | 1 | 1 | 6,00 | 0,00 | 5,00 | 0,00 |
| Q9NRN5 | olfactomedin-like 3 | 10 | 7 | 6,80 | 3,19 | 7,57 | 3,99 |
| Q96CV9 | optineurin | 1 | 1 | 2,00 | 0,00 | 2,00 | 0,00 |
| P20774 | osteoglycin | 2 | 0 | 3,50 | 2,12 | 0,00 | 0,00 |
| Q99983 | osteomodulin | 1 | 1 | 4,00 | 0,00 | 2,00 | 0,00 |
| Q13438 | osteosarcoma amplified 9, endoplasmic reticulum associated protein | 1 | 2 | 4,00 | 0,00 | 2,50 | 0,71 |
| Q96FW1 | OTU domain, ubiquitin aldehyde binding 1 | 1 | 0 | 2,00 | 0,00 | 0,00 | 0,00 |
| O95747 | oxidative-stress responsive 1 | 1 | 0 | 2,00 | 0,00 | 0,00 | 0,00 |
| Q13219 | PAPPA antisense RNA (non-protein coding); pregnancy-associated plasma protein A, pappalysin 1 | 4 | 2 | 2,50 | 1,00 | 2,50 | 0,71 |
| Q99497 | Parkinson disease (autosomal recessive, early onset) 7 | 7 | 8 | 4,71 | 2,63 | 4,75 | 1,98 |
| P26022 | pentraxin-related gene, rapidly induced by IL-1 beta | 10 | 9 | 28,60 | 17,77 | 25,67 | 10,23 |
| P12955 | peptidase D | 5 | 4 | 4,20 | 4,38 | 3,00 | 1,41 |
| Q6UXH9 | peptidase domain containing associated with muscle regeneration 1 | 4 | 3 | 6,25 | 3,30 | 4,33 | 2,08 |
| Q6UXB8 | peptidase inhibitor 16 | 8 | 9 | 13,13 | 8,59 | 10,44 | 6,69 |
| P19021 | peptidylglycine alpha-amidating monooxygenase | 4 | 6 | 6,00 | 4,55 | 6,50 | 4,51 |
| P23284 | peptidylprolyl isomerase B (cyclophilin B) | 9 | 8 | 7,33 | 2,55 | 6,00 | 1,85 |
| Q15063 | periostin, osteoblast specific factor | 10 | 9 | 56,30 | 35,90 | 44,11 | 27,54 |
| Q92626 | peroxidasin homolog (Drosophila) | 10 | 8 | 14,70 | 7,32 | 13,75 | 8,31 |
| Q06830 | peroxiredoxin 1 | 7 | 6 | 4,71 | 1,50 | 4,67 | 1,97 |
| P32119 | peroxiredoxin 2 | 5 | 6 | 2,80 | 0,45 | 3,00 | 0,63 |
| P30048 | peroxiredoxin 3 | 5 | 3 | 2,40 | 0,55 | 2,00 | 0,00 |
| Q13162 | peroxiredoxin 4 | 5 | 6 | 4,40 | 1,14 | 2,67 | 0,82 |
| P30044 | peroxiredoxin 5 | 3 | 2 | 4,00 | 3,46 | 4,50 | 2,12 |
| P30041 | peroxiredoxin 6 | 5 | 5 | 4,20 | 3,83 | 3,60 | 2,61 |
| P30086 | phosphatidylethanolamine binding protein 1 | 6 | 6 | 4,83 | 2,56 | 5,17 | 2,32 |
| P36871 | phosphoglucomutase 1 | 1 | 1 | 3,00 | 0,00 | 2,00 | 0,00 |
| P00558 | phosphoglycerate kinase 1 | 6 | 6 | 6,00 | 5,06 | 6,33 | 3,78 |
| P18669 | phosphoglycerate mutase 1 (brain) | 7 | 7 | 6,57 | 1,99 | 7,00 | 2,08 |
| Q96FE7 | phosphoinositide-3-kinase interacting protein 1 | 1 | 3 | 2,00 | 0,00 | 2,33 | 0,58 |
| Q9Y263 | phospholipase A2-activating protein | 1 | 0 | 2,00 | 0,00 | 0,00 | 0,00 |
| Q8NCC3 | phospholipase A2, group XV | 2 | 2 | 4,00 | 1,41 | 5,50 | 2,12 |
| Q8NHP8 | phospholipase B domain containing 2 | 9 | 7 | 5,44 | 3,13 | 6,29 | 3,59 |
| P55058 | phospholipid transfer protein | 5 | 3 | 3,60 | 1,82 | 4,00 | 2,00 |
| Q9Y646 | plasma glutamate carboxypeptidase | 8 | 7 | 4,00 | 1,77 | 4,43 | 1,27 |
| P00749 | plasminogen activator, urokinase | 1 | 0 | 3,00 | 0,00 | 0,00 | 0,00 |
| Q03405 | plasminogen activator, urokinase receptor | 3 | 5 | 2,33 | 0,58 | 3,40 | 1,14 |
| Q9GZP0 | platelet derived growth factor D | 2 | 3 | 2,00 | 0,00 | 2,00 | 0,00 |
| Q15198 | platelet-derived growth factor receptor-like | 2 | 1 | 2,50 | 0,71 | 2,00 | 0,00 |
| P16234 | platelet-derived growth factor receptor, alpha polypeptide | 1 | 1 | 3,00 | 0,00 | 2,00 | 0,00 |
| P09619 | platelet-derived growth factor receptor, beta polypeptide | 4 | 3 | 2,25 | 0,50 | 2,00 | 0,00 |
| O15031 | plexin B2 | 2 | 1 | 2,50 | 0,71 | 2,00 | 0,00 |
| Q6UX71 | plexin domain containing 2 | 1 | 1 | 2,00 | 0,00 | 2,00 | 0,00 |
| Q7Z5L7 | podocan | 6 | 4 | 3,83 | 1,17 | 3,75 | 1,71 |
| Q15365 | poly(rC) binding protein 1 | 1 | 1 | 8,00 | 0,00 | 4,00 | 0,00 |
| Q6NZI2 | polymerase I and transcript release factor | 4 | 3 | 5,00 | 2,16 | 5,67 | 1,53 |
| P26599 | polypyrimidine tract binding protein 1 | 3 | 4 | 4,33 | 4,04 | 3,00 | 2,00 |
| P0CG47 | Polyubiquitin-B | 4 | 1 | 2,25 | 0,50 | 2,00 | 0,00 |
| P0CG48 | Polyubiquitin-C | 4 | 2 | 2,25 | 0,50 | 4,00 | 2,83 |
| P04156 | prion protein | 2 | 3 | 3,50 | 2,12 | 2,00 | 0,00 |
| Q15113 | procollagen C-endopeptidase enhancer | 10 | 8 | 16,80 | 5,98 | 18,00 | 7,93 |
| Q9UKZ9 | procollagen C-endopeptidase enhancer 2 | 1 | 1 | 9,00 | 0,00 | 8,00 | 0,00 |
| Q02809 | procollagen-lysine 1, 2-oxoglutarate 5-dioxygenase 1 | 10 | 8 | 8,10 | 3,41 | 7,25 | 3,37 |
| O00469 | procollagen-lysine, 2-oxoglutarate 5-dioxygenase 2 | 3 | 2 | 3,33 | 2,31 | 6,00 | 2,83 |
| O60568 | procollagen-lysine, 2-oxoglutarate 5-dioxygenase 3 | 9 | 7 | 5,11 | 4,31 | 4,43 | 3,21 |
| O14737 | programmed cell death 5 | 1 | 1 | 2,00 | 0,00 | 2,00 | 0,00 |
| P51888 | proline/arginine-rich end leucine-rich repeat protein | 1 | 2 | 2,00 | 0,00 | 4,00 | 1,41 |
| P07237 | prolyl 4-hydroxylase, beta polypeptide | 10 | 9 | 21,80 | 11,57 | 23,33 | 10,34 |
| P42785 | prolylcarboxypeptidase (angiotensinase C) | 5 | 5 | 3,40 | 1,14 | 3,60 | 1,52 |
| Q92824 | proprotein convertase subtilisin/kexin type 5 | 1 | 0 | 3,00 | 0,00 | 0,00 | 0,00 |
| Q8NBP7 | proprotein convertase subtilisin/kexin type 9 | 8 | 4 | 7,63 | 6,93 | 11,25 | 5,91 |
| P07602 | prosaposin | 10 | 9 | 38,40 | 24,05 | 37,22 | 15,54 |
| P41222 | prostaglandin D2 synthase, hematopoietic; prostaglandin D2 synthase 21kDa (brain) | 10 | 9 | 5,60 | 2,99 | 6,44 | 4,25 |
| Q14914 | prostaglandin reductase 1 | 1 | 1 | 2,00 | 0,00 | 2,00 | 0,00 |
| O95084 | protease, serine, 23 | 5 | 6 | 3,40 | 1,14 | 3,33 | 1,51 |
| Q13200 | proteasome (prosome, macropain) 26S subunit, non-ATPase, 2 | 1 | 0 | 2,00 | 0,00 | 0,00 | 0,00 |
| Q06323 | proteasome (prosome, macropain) activator subunit 1 (PA28 alpha) | 1 | 1 | 5,00 | 0,00 | 7,00 | 0,00 |
| Q9UL46 | proteasome (prosome, macropain) activator subunit 2 (PA28 beta) | 4 | 2 | 2,25 | 0,50 | 2,50 | 0,71 |
| Q9UNN8 | protein C receptor, endothelial (EPCR) | 5 | 4 | 3,60 | 1,82 | 3,75 | 1,26 |
| P30101 | protein disulfide isomerase family A, member 3 | 10 | 9 | 13,90 | 4,09 | 13,44 | 4,03 |
| P13667 | protein disulfide isomerase family A, member 4 | 7 | 6 | 6,86 | 2,97 | 8,17 | 3,06 |
| Q15084 | protein disulfide isomerase family A, member 6 | 9 | 7 | 5,22 | 2,28 | 5,29 | 2,56 |
| P14314 | protein kinase C substrate 80K-H | 9 | 8 | 4,00 | 1,58 | 4,88 | 2,53 |
| P07225 | protein S (alpha) | 3 | 2 | 3,67 | 0,58 | 2,50 | 0,71 |
| P23470 | protein tyrosine phosphatase, receptor type, G | 1 | 3 | 2,00 | 0,00 | 2,33 | 0,58 |
| Q13332 | protein tyrosine phosphatase, receptor type, S | 1 | 1 | 2,00 | 0,00 | 3,00 | 0,00 |
| Q92954 | proteoglycan 4 | 4 | 3 | 4,00 | 3,37 | 5,00 | 4,36 |
| Q13308 | PTK7 protein tyrosine kinase 7 | 5 | 3 | 4,20 | 2,59 | 2,33 | 0,58 |
| Q8NHM4 | Putative trypsin-6 | 1 | 1 | 6,00 | 0,00 | 5,00 | 0,00 |
| O00764 | pyridoxal (pyridoxine, vitamin B6) kinase | 1 | 2 | 4,00 | 0,00 | 3,00 | 1,41 |
| O00391 | quiescin Q6 sulfhydryl oxidase 1 | 10 | 8 | 21,00 | 8,19 | 20,88 | 8,18 |
| P61019 | RAB2A, member RAS oncogene family | 1 | 0 | 2,00 | 0,00 | 0,00 | 0,00 |
| P51149 | RAB7A, member RAS oncogene family | 1 | 1 | 7,00 | 0,00 | 6,00 | 0,00 |
| P61586 | ras homolog gene family, member A | 1 | 0 | 5,00 | 0,00 | 0,00 | 0,00 |
| Q15404 | Ras suppressor protein 1 | 1 | 1 | 5,00 | 0,00 | 2,00 | 0,00 |
| P62070 | related RAS viral (r-ras) oncogene homolog 2 | 1 | 0 | 2,00 | 0,00 | 0,00 | 0,00 |
| Q15293 | reticulocalbin 1, EF-hand calcium binding domain | 10 | 9 | 8,60 | 1,84 | 11,67 | 1,87 |
| Q14257 | reticulocalbin 2, EF-hand calcium binding domain | 4 | 4 | 2,75 | 0,96 | 3,75 | 2,06 |
| Q96D15 | reticulocalbin 3, EF-hand calcium binding domain | 9 | 8 | 4,78 | 2,91 | 6,00 | 2,27 |
| Q9NQC3 | reticulon 4 | 4 | 4 | 3,75 | 2,87 | 3,00 | 1,41 |
| Q99969 | retinoic acid receptor responder (tazarotene induced) 2 | 5 | 5 | 3,80 | 1,92 | 3,40 | 1,52 |
| O95980 | reversion-inducing-cysteine-rich protein with kazal motifs | 2 | 2 | 4,50 | 0,71 | 3,50 | 0,71 |
| Q6NW40 | RGM domain family, member B | 1 | 0 | 3,00 | 0,00 | 0,00 | 0,00 |
| O00584 | ribonuclease T2 | 2 | 4 | 3,00 | 0,00 | 3,25 | 0,96 |
| P05387 | ribosomal protein, large, P2 pseudogene 3; ribosomal protein, large, P2 | 7 | 8 | 4,43 | 1,13 | 4,50 | 1,07 |
| Q99829 | RNA binding motif protein 12; copine I | 1 | 1 | 3,00 | 0,00 | 2,00 | 0,00 |
| Q9Y6N7 | roundabout, axon guidance receptor, homolog 1 | 1 | 0 | 2,00 | 0,00 | 0,00 | 0,00 |
| P55735 | SEC13 homolog (S. cerevisiae) | 1 | 0 | 2,00 | 0,00 | 0,00 | 0,00 |
| Q96HF1 | secreted frizzled-related protein 2 | 2 | 2 | 10,50 | 7,78 | 3,50 | 2,12 |
| Q6FHJ7 | secreted frizzled-related protein 4 | 6 | 7 | 6,00 | 4,29 | 3,14 | 1,07 |
| P10451 | secreted phosphoprotein 1 | 1 | 1 | 2,00 | 0,00 | 2,00 | 0,00 |
| P09486 | secreted protein, acidic, cysteine-rich (osteonectin) | 10 | 9 | 70,30 | 54,69 | 68,44 | 26,88 |
| P13521 | secretogranin II (chromogranin C) | 4 | 5 | 8,25 | 4,19 | 5,20 | 1,79 |
| Q8WWX9 | selenoprotein M | 2 | 3 | 2,50 | 0,71 | 3,00 | 1,00 |
| O75326 | semaphorin 7A, GPI membrane anchor (John Milton Hagen blood group) | 10 | 8 | 7,50 | 4,30 | 8,25 | 4,33 |
| Q15019 | septin 2 | 3 | 2 | 2,00 | 0,00 | 2,50 | 0,71 |
| Q9UHD8 | septin 9 | 2 | 2 | 4,00 | 2,83 | 2,50 | 0,71 |
| P10124 | serglycin | 2 | 2 | 2,00 | 0,00 | 2,00 | 0,00 |
| P01008 | serpin peptidase inhibitor, clade C (antithrombin), member 1 | 1 | 2 | 2,00 | 0,00 | 2,50 | 0,71 |
| P05121 | serpin peptidase inhibitor, clade E (nexin, plasminogen activator inhibitor type 1), member 1 | 10 | 9 | 42,50 | 21,17 | 35,00 | 14,32 |
| P07093 | serpin peptidase inhibitor, clade E (nexin, plasminogen activator inhibitor type 1), member 2 | 10 | 8 | 29,40 | 19,84 | 25,63 | 11,46 |
| P36955 | serpin peptidase inhibitor, clade F (alpha-2 antiplasmin, pigment epithelium derived factor), member 1 | 10 | 9 | 18,50 | 8,78 | 19,00 | 11,20 |
| P05155 | serpin peptidase inhibitor, clade G (C1 inhibitor), member 1 | 10 | 10 | 9,60 | 7,04 | 9,20 | 6,61 |
| P50454 | serpin peptidase inhibitor, clade H (heat shock protein 47), member 1, (collagen binding protein 1) | 10 | 8 | 14,30 | 8,54 | 13,75 | 5,50 |
| Q01105 | SET nuclear oncogene; similar to SET translocation | 6 | 9 | 3,17 | 0,75 | 3,67 | 1,41 |
| Q9HAT2 | sialic acid acetylesterase | 4 | 3 | 4,50 | 2,38 | 5,33 | 2,52 |
| Q99519 | sialidase 1 (lysosomal sialidase) | 3 | 2 | 3,67 | 2,08 | 2,50 | 0,71 |
| Q8IX30 | signal peptide, CUB domain, EGF-like 3 | 2 | 1 | 4,00 | 1,41 | 3,00 | 0,00 |
| P42224 | signal transducer and activator of transcription 1, 91kDa | 1 | 0 | 7,00 | 0,00 | 0,00 | 0,00 |
| Q9H173 | SIL1 homolog, endoplasmic reticulum chaperone (S. cerevisiae) | 1 | 0 | 2,00 | 0,00 | 0,00 | 0,00 |
| P01024 | similar to Complement C3 precursor; complement component 3; hypothetical protein LOC100133511 | 8 | 8 | 14,13 | 13,04 | 35,25 | 51,83 |
| Q14393 | similar to growth arrest-specific 6; growth arrest-specific 6 | 8 | 8 | 7,75 | 4,40 | 8,00 | 5,07 |
| P62937 | similar to TRIMCyp; peptidylprolyl isomerase A (cyclophilin A); peptidylprolyl isomerase A (cyclophilin A)-like 3 | 8 | 8 | 9,38 | 5,07 | 10,00 | 2,00 |
| O75094 | slit homolog 3 (Drosophila) | 3 | 3 | 7,00 | 4,36 | 4,67 | 3,79 |
| A1L4H1 | soluble scavenger receptor cysteine-rich domain-containing protein SSC5D | 10 | 9 | 6,40 | 3,63 | 7,78 | 4,89 |
| Q9UMY4 | sorting nexin 12 | 1 | 0 | 2,00 | 0,00 | 0,00 | 0,00 |
| O60493 | sorting nexin 3 | 1 | 1 | 2,00 | 0,00 | 2,00 | 0,00 |
| Q9Y5X1 | sorting nexin 9 | 1 | 1 | 3,00 | 0,00 | 3,00 | 0,00 |
| Q08629 | sparc/osteonectin, cwcv and kazal-like domains proteoglycan (testican) 1 | 10 | 9 | 8,90 | 4,46 | 10,22 | 5,09 |
| P17405 | sphingomyelin phosphodiesterase 1, acid lysosomal | 2 | 2 | 5,00 | 1,41 | 3,50 | 0,71 |
| Q92484 | sphingomyelin phosphodiesterase, acid-like 3A | 1 | 2 | 3,00 | 0,00 | 2,50 | 0,71 |
| Q9HCB6 | spondin 1, extracellular matrix protein | 2 | 2 | 5,00 | 1,41 | 2,50 | 0,71 |
| Q9BUD6 | spondin 2, extracellular matrix protein | 9 | 7 | 7,33 | 4,21 | 8,00 | 5,00 |
| P52823 | stanniocalcin 1 | 2 | 4 | 3,00 | 0,00 | 3,25 | 0,96 |
| O76061 | stanniocalcin 2 | 9 | 8 | 3,67 | 1,66 | 5,25 | 2,87 |
| P16949 | stathmin 1 | 2 | 3 | 3,00 | 0,00 | 2,33 | 0,58 |
| O95793 | staufen, RNA binding protein, homolog 1 (Drosophila) | 1 | 0 | 3,00 | 0,00 | 0,00 | 0,00 |
| Q9H2G2 | STE20-like kinase (yeast) | 1 | 1 | 2,00 | 0,00 | 3,00 | 0,00 |
| P27105 | stomatin | 1 | 0 | 3,00 | 0,00 | 0,00 | 0,00 |
| P31948 | stress-induced-phosphoprotein 1 | 1 | 1 | 3,00 | 0,00 | 4,00 | 0,00 |
| Q9BRK5 | stromal cell derived factor 4 | 10 | 9 | 6,80 | 3,55 | 8,00 | 2,96 |
| Q8NBK3 | sulfatase modifying factor 1 | 1 | 0 | 2,00 | 0,00 | 0,00 | 0,00 |
| Q8NBJ7 | sulfatase modifying factor 2 | 2 | 2 | 3,00 | 1,41 | 3,00 | 1,41 |
| P08294 | superoxide dismutase 3, extracellular | 6 | 6 | 4,33 | 2,42 | 3,67 | 1,21 |
| P78539 | sushi-repeat-containing protein, X-linked | 4 | 2 | 3,00 | 1,41 | 4,50 | 2,12 |
| O60687 | sushi-repeat-containing protein, X-linked 2 | 1 | 1 | 2,00 | 0,00 | 2,00 | 0,00 |
| Q8TER0 | sushi, nidogen and EGF-like domains 1 | 1 | 1 | 3,00 | 0,00 | 2,00 | 0,00 |
| Q4LDE5 | sushi, von Willebrand factor type A, EGF and pentraxin domain containing 1 | 7 | 5 | 12,29 | 9,71 | 10,60 | 7,77 |
| O15400 | syntaxin 7 | 1 | 1 | 3,00 | 0,00 | 2,00 | 0,00 |
| Q9Y490 | talin 1 | 5 | 5 | 12,80 | 11,26 | 9,60 | 7,89 |
| P24821 | tenascin C | 10 | 8 | 18,20 | 12,25 | 16,00 | 17,85 |
| P22105 | tenascin XB; tenascin XA pseudogene | 6 | 5 | 48,17 | 23,22 | 36,00 | 26,11 |
| O95881 | thioredoxin domain containing 12 (endoplasmic reticulum) | 1 | 0 | 2,00 | 0,00 | 0,00 | 0,00 |
| Q8NBS9 | thioredoxin domain containing 5 (endoplasmic reticulum); muted homolog (mouse) | 7 | 7 | 5,43 | 3,10 | 4,43 | 1,99 |
| Q86V81 | THO complex 4 | 1 | 3 | 5,00 | 0,00 | 4,00 | 1,00 |
| P07996 | thrombospondin 1 | 10 | 10 | 33,10 | 16,82 | 29,60 | 16,04 |
| P35442 | thrombospondin 2 | 10 | 8 | 39,50 | 23,41 | 34,25 | 10,70 |
| P49746 | thrombospondin 3 | 4 | 1 | 3,00 | 1,41 | 5,00 | 0,00 |
| P01033 | TIMP metallopeptidase inhibitor 1 | 10 | 9 | 30,80 | 14,04 | 30,67 | 12,78 |
| P16035 | TIMP metallopeptidase inhibitor 2 | 10 | 9 | 18,30 | 9,58 | 15,33 | 6,58 |
| P10646 | tissue factor pathway inhibitor (lipoprotein-associated coagulation inhibitor) | 4 | 5 | 2,75 | 0,96 | 3,20 | 1,64 |
| P48307 | tissue factor pathway inhibitor 2 | 2 | 2 | 3,00 | 1,41 | 2,00 | 0,00 |
| P20062 | transcobalamin II; macrocytic anemia | 2 | 1 | 2,00 | 0,00 | 2,00 | 0,00 |
| P02787 | transferrin | 1 | 2 | 3,00 | 0,00 | 2,50 | 0,71 |
| Q03167 | transforming growth factor, beta receptor III | 2 | 1 | 3,00 | 1,41 | 4,00 | 0,00 |
| Q15582 | transforming growth factor, beta-induced, 68kDa | 10 | 9 | 45,50 | 36,09 | 46,11 | 33,37 |
| P29401 | transketolase | 7 | 6 | 6,14 | 4,67 | 4,83 | 2,56 |
| P02766 | transthyretin | 1 | 1 | 2,00 | 0,00 | 3,00 | 0,00 |
| O14773 | tripeptidyl peptidase I | 10 | 8 | 14,00 | 8,03 | 14,13 | 7,10 |
| P09493 | tropomyosin 1 (alpha) | 10 | 9 | 9,40 | 4,58 | 12,11 | 4,70 |
| P06753 | tropomyosin 3 | 10 | 8 | 6,10 | 3,28 | 6,75 | 2,12 |
| P67936 | tropomyosin 4 | 10 | 9 | 11,50 | 6,98 | 13,33 | 7,55 |
| O75347 | tubulin folding cofactor A | 2 | 3 | 2,00 | 0,00 | 2,00 | 0,00 |
| Q9GZM7 | tubulointerstitial nephritis antigen-like 1 | 1 | 1 | 4,00 | 0,00 | 5,00 | 0,00 |
| O00300 | tumor necrosis factor receptor superfamily, member 11b | 1 | 1 | 3,00 | 0,00 | 2,00 | 0,00 |
| Q9NP84 | tumor necrosis factor receptor superfamily, member 12A | 3 | 3 | 3,67 | 1,15 | 4,33 | 1,53 |
| P98066 | tumor necrosis factor, alpha-induced protein 6 | 3 | 2 | 3,33 | 1,53 | 2,50 | 0,71 |
| Q6IBS0 | twinfilin, actin-binding protein, homolog 2 (Drosophila) | 1 | 1 | 5,00 | 0,00 | 2,00 | 0,00 |
| P61088 | ubiquitin-conjugating enzyme E2N (UBC13 homolog, yeast) | 1 | 0 | 4,00 | 0,00 | 0,00 | 0,00 |
| P22314 | ubiquitin-like modifier activating enzyme 1 | 2 | 3 | 7,50 | 4,95 | 4,67 | 4,62 |
| Q9NYU2 | UDP-glucose ceramide glucosyltransferase-like 1 | 1 | 1 | 2,00 | 0,00 | 2,00 | 0,00 |
| O60701 | UDP-glucose dehydrogenase | 2 | 1 | 8,00 | 7,07 | 7,00 | 0,00 |
| Q10471 | UDP-N-acetyl-alpha-D-galactosamine:polypeptide N-acetylgalactosaminyltransferase 2 (GalNAc-T2) | 4 | 4 | 3,75 | 1,26 | 4,00 | 2,31 |
| Q7Z7M9 | UDP-N-acetyl-alpha-D-galactosamine:polypeptide N-acetylgalactosaminyltransferase 5 (GalNAc-T5) | 1 | 1 | 2,00 | 0,00 | 5,00 | 0,00 |
| Q86SR1 | UDP-N-acetyl-alpha-D-galactosamine:polypeptide N-acetylgalactosaminyltransferase 10 (GalNAc-T10) | 1 | 1 | 5,00 | 0,00 | 3,00 | 0,00 |
| P46108 | v-crk sarcoma virus CT10 oncogene homolog (avian) | 1 | 1 | 4,00 | 0,00 | 5,00 | 0,00 |
| Q9P0L0 | VAMP (vesicle-associated membrane protein)-associated protein A, 33kDa | 1 | 1 | 3,00 | 0,00 | 2,00 | 0,00 |
| P49767 | vascular endothelial growth factor C | 3 | 3 | 2,67 | 1,15 | 4,67 | 1,15 |
| P50552 | vasodilator-stimulated phosphoprotein | 1 | 0 | 4,00 | 0,00 | 0,00 | 0,00 |
| Q6EMK4 | vasorin | 9 | 8 | 9,67 | 5,43 | 8,63 | 2,92 |
| P13611 | versican | 10 | 9 | 21,20 | 9,13 | 19,44 | 6,42 |
| Q99536 | vesicle amine transport protein 1 homolog (T. californica) | 2 | 2 | 6,00 | 2,83 | 4,50 | 2,12 |
| P04004 | vitronectin | 2 | 3 | 3,00 | 1,41 | 2,67 | 0,58 |
| O75083 | WD repeat domain 1 | 1 | 1 | 5,00 | 0,00 | 3,00 | 0,00 |
| O76076 | WNT1 inducible signaling pathway protein 2 | 7 | 6 | 5,00 | 2,58 | 5,50 | 2,26 |
| P12956 | X-ray repair complementing defective repair in Chinese hamster cells 6; similar to ATP-dependent DNA helicase II, 70 kDa subunit | 2 | 1 | 2,50 | 0,71 | 2,00 | 0,00 |
| P13010 | X-ray repair complementing defective repair in Chinese hamster cells 5 (double-strand-break rejoining) | 2 | 1 | 2,50 | 0,71 | 4,00 | 0,00 |

**Table S5. List of extracellular matrix proteins found in secretomes of normoxic ADSCs**

ADAM metallopeptidase with thrombospondin type 1 motif, 1

ADAM metallopeptidase with thrombospondin type 1 motif, 2

ADAMTS-like 1

aggrecan

agrin

angiopoietin-like 4

annexin A2 pseudogene 3; annexin A2; annexin A2 pseudogene 1

biglycan

calreticulin

**cartilage associated protein**

cartilage oligomeric matrix protein

CD248 molecule, endosialin

chitinase 3-like 1 (cartilage glycoprotein-39)

coiled-coil domain containing 80

collagen triple helix repeat containing 1

collagen, type I, alpha 1

collagen, type I, alpha 2

collagen, type III, alpha 1

collagen, type IV, alpha 1

collagen, type IV, alpha 2

collagen, type V, alpha 1

collagen, type V, alpha 2

collagen, type VI, alpha 1

collagen, type VI, alpha 2

collagen, type VI, alpha 3

collagen, type VII, alpha 1

**collagen, type VIII, alpha 1**

collagen, type XI, alpha 1

collagen, type XII, alpha 1

collagen, type XIV, alpha 1

collagen, type XV, alpha 1

collagen, type XVI, alpha 1

collagen, type XVIII, alpha 1

connective tissue growth factor

decorin

dermatopontin

dystroglycan 1 (dystrophin-associated glycoprotein 1)

EGF-containing fibulin-like extracellular matrix protein 1

EGF-containing fibulin-like extracellular matrix protein 2

elastin

elastin microfibril interfacer 1

elastin microfibril interfacer 2

extracellular matrix protein 1

**extracellular matrix protein 2, female organ and adipocyte specific**

fibrillin 1

fibrillin 2

fibromodulin

fibronectin 1

fibulin 1

fibulin 2

fibulin 5

glypican 1

glypican 6

hemicentin 1

heparan sulfate proteoglycan 2

hyaluronan and proteoglycan link protein 1

**hyaluronan and proteoglycan link protein 3**

laminin, alpha 1

laminin, alpha 2

laminin, alpha 4

laminin, alpha 5

laminin, beta 1

laminin, beta 2 (laminin S)

laminin, gamma 1 (formerly LAMB2)

latent transforming growth factor beta binding protein 1

latent transforming growth factor beta binding protein 2

lectin, galactoside-binding, soluble, 3 binding protein

lumican

lysyl oxidase

lysyl oxidase-like 1

**matrilin 3**

matrix metallopeptidase 1 (interstitial collagenase)

matrix metallopeptidase 14 (membrane-inserted)

**matrix metallopeptidase 19**

matrix metallopeptidase 2 (gelatinase A, 72kDa gelatinase, 72kDa type IV collagenase)

matrix metallopeptidase 3 (stromelysin 1, progelatinase)

matrix metallopeptidase 9 (gelatinase B, 92kDa gelatinase, 92kDa type IV collagenase)

microfibrillar associated protein 5

microfibrillar-associated protein 2

microfibrillar-associated protein 4

nidogen 1

nidogen 2 (osteonidogen)

**osteoglycin**

osteomodulin

periostin, osteoblast specific factor

podocan

proline/arginine-rich end leucine-rich repeat protein

secreted protein, acidic, cysteine-rich (osteonectin)

sparc/osteonectin, cwcv and kazal-like domains proteoglycan (testican) 1

spondin 1, extracellular matrix protein

spondin 2, extracellular matrix protein

tenascin C

tenascin XB; tenascin XA pseudogene

TIMP metallopeptidase inhibitor 1

TIMP metallopeptidase inhibitor 2

tissue factor pathway inhibitor 2

transferrin

transforming growth factor, beta-induced, 68kDa

tumor necrosis factor receptor superfamily, member 11b

versican

vitronectin

*Proteins labelled by **bold** font were not found in hypoxic samples

In hypoxic samples were additionally found:

glypican 4

leucine proline-enriched proteoglycan (leprecan) 1

matrix metallopeptidase 8 (neutrophil collagenase)

serpin peptidase inhibitor, clade A (alpha-1 antiproteinase, antitrypsin), member 1

**Table S6. List of proteins involved in blood vessel development, which were found in secretomes of normoxic ADSCs**

angiopoietin-like 4

angiotensin I converting enzyme (peptidyl-dipeptidase A) 1

angiotensinogen (serpin peptidase inhibitor, clade A, member 8)

annexin A2

biglycan

cadherin 13, H-cadherin (heart)

cadherin 2, type 1, N-cadherin (neuronal)

CD44 molecule (Indian blood group)

chondroitin sulfate proteoglycan 4

collagen, type I, alpha 1

collagen, type I, alpha 2

collagen, type III, alpha 1

collagen, type V, alpha 1

collagen, type XV, alpha 1

collagen, type XVIII, alpha 1

connective tissue growth factor

cysteine-rich, angiogenic inducer, 61

endoglin

endoplasmic reticulum aminopeptidase 1

apolipoprotein E

integrin, alpha V (vitronectin receptor, alpha polypeptide, antigen CD51)

interleukin 8

laminin, alpha 4

laminin, alpha 5

lysyl oxidase

matrix metallopeptidase 14 (membrane-inserted)

matrix metallopeptidase 19

matrix metallopeptidase 2 (gelatinase A, 72kDa gelatinase, 72kDa type IV collagenase)

myosin, heavy chain 9, non-muscle

neuropilin 1

neuropilin 2

plasminogen activator, urokinase

reticulon 4

reversion-inducing-cysteine-rich protein with kazal motifs

roundabout, axon guidance receptor, homolog 1 (Drosophila); similar to roundabout 1 isoform b

secretogranin II (chromogranin C)

thrombospondin 1

transforming growth factor, beta receptor III

tumor necrosis factor receptor superfamily, member 12A

vascular endothelial growth factor C

**Table S7. List of proteins involved in neuron differentiation, which were found in secretomes of normoxic ADSCs**

actin, beta

agrin

amyloid beta (A4) precursor protein

CD44 molecule (Indian blood group)

ceroid-lipofuscinosis, neuronal 5

clusterin

epidermal growth factor receptor

fibroblast growth factor receptor 1

growth arrest-specific 1

activated leukocyte cell adhesion molecule (ALCAM)

interleukin 6 (interferon, beta 2)

laminin, beta 1

laminin, beta 2 (laminin S)

myosin, heavy chain 10, non-muscle

neurofascin homolog (chicken)

neuropilin 1

neuropilin 2

neurotrimin

proprotein convertase subtilisin/kexin type 9

roundabout, axon guidance receptor 1 isoform b

septin 2

slit homolog 3

spondin 2, extracellular matrix protein

vasodilator-stimulated phosphoprotein

versican

**Table S8. Proteins found only in secretomes of hypoxic ADSCs**

| **UNIPROT_**  **ACCESSION** | **Protein Name** | **Number of samples** | **Number of peptides**  **M SD** | |
| --- | --- | --- | --- | --- |
| Q8TCD5 | 5', 3'-nucleotidase, cytosolic | 1 | 2,0 | NA |
| P35318 | adrenomedullin | 3 | 2,3 | 0,58 |
| P09972 | aldolase C, fructose-bisphosphate | 1 | 5,0 | NA |
| Q9H6X2 | anthrax toxin receptor 1 | 1 | 2,00 | NA |
| P02647 | apolipoprotein A-I | 1 | 19,0 | NA |
| P02652 | apolipoprotein A-II | 1 | 2,0 | NA |
| P04114 | apolipoprotein B (including Ag(x) antigen) | 1 | 10,0 | NA |
| O14791 | apolipoprotein L, 1 | 1 | 3,0 | NA |
| Q9H4A4 | arginyl aminopeptidase (aminopeptidase B) | 1 | 2,0 | NA |
| Q9ULA0 | aspartyl aminopeptidase | 1 | 3,0 | NA |
| Q96IY4 | carboxypeptidase B2 (plasma) | 1 | 7,0 | NA |
| Q8N436 | carboxypeptidase X (M14 family), member 2 | 1 | 2,0 | NA |
| P43234 | cathepsin O | 1 | 2,0 | NA |
| Q5ZPR3 | CD276 molecule | 1 | 2,0 | NA |
| P00450 | ceruloplasmin (ferroxidase) | 1 | 19,0 | NA |
| P13500 | chemokine (C-C motif) ligand 2 | 1 | 2,0 | NA |
| P02778 | chemokine (C-X-C motif) ligand 10 | 2 | 2,0 | 0,00 |
| P80162 | chemokine (C-X-C motif) ligand 6 (granulocyte chemotactic protein 2) | 1 | 2,0 | NA |
| P00748 | coagulation factor XII (Hageman factor) | 1 | 5,0 | NA |
| P09919 | colony stimulating factor 3 (granulocyte) | 1 | 2,0 | NA |
| P01031 | complement component 5 | 1 | 42,0 | NA |
| P13671 | complement component 6 | 1 | 12,0 | NA |
| P10643 | complement component 7 | 1 | 5,0 | NA |
| P07358 | complement component 8, beta polypeptide | 1 | 15,0 | NA |
| P02748 | complement component 9 | 1 | 17,0 | NA |
| Q9NZV1 | cysteine rich transmembrane BMP regulator 1 (chordin-like) | 2 | 4,00 | 1,41 |
| Q6E0U4 | dermokine | 1 | 2,0 | NA |
| Q14203 | dynactin 1 (p150, glued homolog, Drosophila) | 1 | 2,0 | NA |
| O43854 | EGF-like repeats and discoidin I-like domains 3 | 3 | 2,3 | 0,58 |
| Q14517 | FAT tumor suppressor homolog 1 (Drosophila) | 2 | 2,0 | 0,00 |
| P02671 | fibrinogen alpha chain | 1 | 10,0 | NA |
| O75636 | ficolin (collagen/fibrinogen domain containing) 3 (Hakata antigen) | 1 | 2,0 | NA |
| O75487 | glypican 4 | 1 | 2,0 | NA |
| P02774 | group-specific component (vitamin D binding protein) | 1 | 2,0 | NA |
| Q6KF10 | growth differentiation factor 6 | 1 | 2,0 | NA |
| Q9ULI3 | HEG homolog 1 (zebrafish) | 1 | 2,00 | NA |
| P02790 | hemopexin | 1 | 14,0 | NA |
| Q29940 | HLA class I histocompatibility antigen, B-59 alpha chain | 1 | 3,0 | NA |
| P01857 | immunoglobulin heavy constant gamma 1 (G1m marker); | 1 | 5,0 | NA |
| P11717 | insulin-like growth factor 2 receptor | 1 | 2,0 | NA |
| P35858 | insulin-like growth factor binding protein, acid labile subunit | 1 | 3,0 | NA |
| P19827 | inter-alpha (globulin) inhibitor H1 | 1 | 2,0 | NA |
| Q14624 | inter-alpha (globulin) inhibitor H4 (plasma Kallikrein-sensitive glycoprotein) | 1 | 10,0 | NA |
| P03952 | kallikrein B, plasma (Fletcher factor) 1 | 1 | 2,0 | NA |
| P01042 | kininogen 1 | 1 | 8,0 | NA |
| Q9NS15 | latent transforming growth factor beta binding protein 3 | 1 | 2,0 | NA |
| P41159 | leptin | 1 | 2,0 | NA |
| Q32P28 | leucine proline-enriched proteoglycan (leprecan) 1 | 2 | 2,0 | 0,00 |
| P15018 | leukemia inhibitory factor (cholinergic differentiation factor) | 1 | 2,0 | NA |
| P58215 | lysyl oxidase-like 3 | 1 | 3,0 | NA |
| P10319 | major histocompatibility complex, class I, C; major histocompatibility complex, class I, B | 1 | 3,0 | NA |
| P18463 | major histocompatibility complex, class I, C; major histocompatibility complex, class I, B | 1 | 2,0 | NA |
| P30460 | major histocompatibility complex, class I, C; major histocompatibility complex, class I, B | 1 | 3,0 | NA |
| P30462 | major histocompatibility complex, class I, C; major histocompatibility complex, class I, B | 1 | 2,0 | NA |
| P30466 | major histocompatibility complex, class I, C; major histocompatibility complex, class I, B | 1 | 2,0 | NA |
| P30479 | major histocompatibility complex, class I, C; major histocompatibility complex, class I, B | 2 | 4,0 | 1,41 |
| P30480 | major histocompatibility complex, class I, C; major histocompatibility complex, class I, B | 1 | 3,0 | NA |
| P30492 | major histocompatibility complex, class I, C; major histocompatibility complex, class I, B | 1 | 2,0 | NA |
| P30493 | major histocompatibility complex, class I, C; major histocompatibility complex, class I, B | 1 | 3,0 | NA |
| P30499 | major histocompatibility complex, class I, C; major histocompatibility complex, class I, B | 1 | 3,0 | NA |
| P30505 | major histocompatibility complex, class I, C; major histocompatibility complex, class I, B | 2 | 5,5 | 3,54 |
| Q29963 | major histocompatibility complex, class I, C; major histocompatibility complex, class I, B | 1 | 3,0 | NA |
| Q9TNN7 | major histocompatibility complex, class I, C; major histocompatibility complex, class I, B | 1 | 3,00 | NA |
| P26572 | mannosyl (alpha-1,3-)-glycoprotein beta-1,2-N-acetylglucosaminyltransferase | 2 | 2,0 | 0,00 |
| P22894 | matrix metallopeptidase 8 (neutrophil collagenase) | 1 | 2,0 | NA |
| P21741 | midkine (neurite growth-promoting factor 2) | 1 | 2,0 | NA |
| Q7Z7M0 | multiple EGF-like-domains 8 | 1 | 2,0 | NA |
| Q9NTK5 | Obg-like ATPase 1 | 1 | 2,00 | NA |
| Q68BL8 | olfactomedin-like 2B | 1 | 3,0 | NA |
| P27169 | paraoxonase 1 | 1 | 2,0 | NA |
| P11216 | phosphorylase, glycogen; brain | 1 | 2,0 | NA |
| P00747 | plasminogen | 1 | 12,0 | NA |
| P00750 | plasminogen activator, tissue | 1 | 2,0 | NA |
| O15460 | prolyl 4-hydroxylase, alpha polypeptide II | 1 | 2,0 | NA |
| P07477 | protease, serine, 1 (trypsin 1); trypsinogen C | 1 | 2,0 | NA |
| P62745 | ras homolog gene family, member B | 1 | 2,0 | NA |
| P07998 | ribonuclease, RNase A family, 1 (pancreatic) | 1 | 2,0 | NA |
| P34096 | ribonuclease, RNase A family, 4 | 3 | 2,0 | 0,00 |
| P01009 | serpin peptidase inhibitor, clade A (alpha-1 antiproteinase, antitrypsin), member 1 | 1 | 9,0 | NA |
| P05546 | serpin peptidase inhibitor, clade D (heparin cofactor), member 1 | 1 | 3,0 | NA |
| Q8N114 | shisa homolog 5 (Xenopus laevis) | 1 | 2,0 | NA |
| P78324 | Tyrosine-protein phosphatase non-receptor type substrate 1 | 3 | 2,0 | 0,00 |
| Q5TFQ8 | signal-regulatory protein beta 1 | 1 | 2,0 | NA |
| O94813 | slit homolog 2 (Drosophila) | 1 | 2,0 | NA |
| P31431 | syndecan 4 | 1 | 2,0 | NA |
