# Supplementary figures and images for: Characterization of secretomes provides evidence for adipose-derived mesenchymal stromal cells subtypes
Source: Stem Cell Res Ther. 2015 Nov 11;6:221. doi: 10.1186/s13287-015-0209-8 (PMC4642680; doi:10.1186/s13287-015-0209-8)

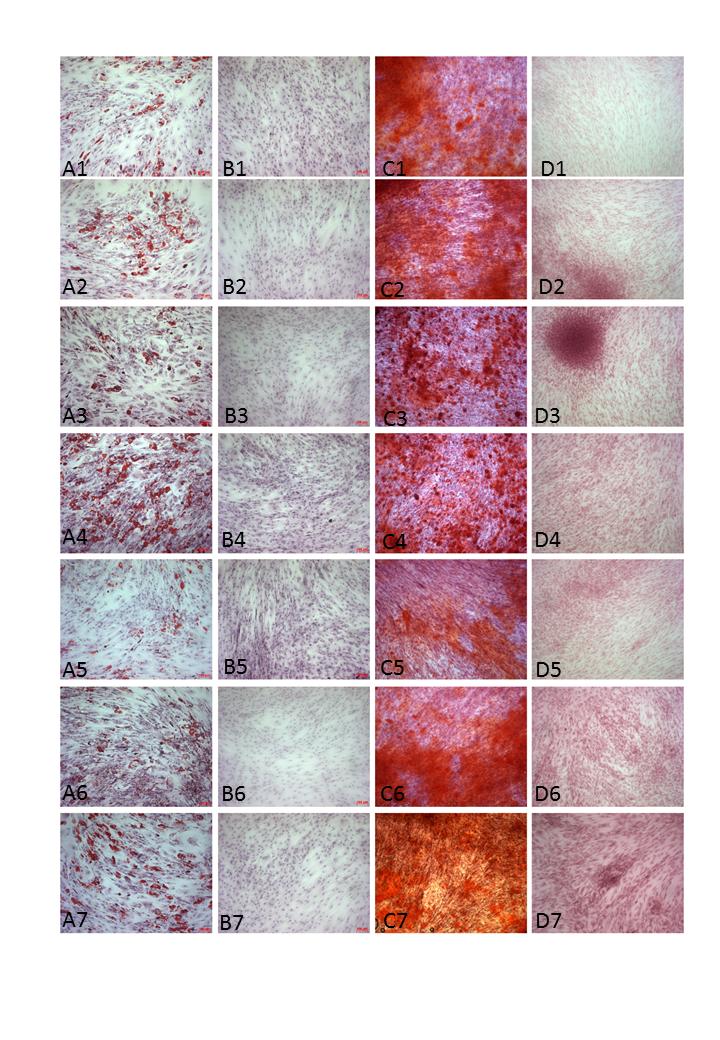

Supplement: Additional file 2: Figure S1. — Representative images of ADSCs undergoing adipogenic or osteogenic differentiation. A1-A7. – Cells incubated in adipogenesis inducing medium for 27 days. C1-C7. - Cells incubated in osteogenesis inducing medium for 21 day. A, B. – Cells stained with Oil-red O to detect lipid accumulations. C, D. - Cells stained with Alizarin red S to detect calcium accumulations. Nuclei were counterstained by hematoxilin. Scale = 100 μm. (TIFF 1643 kb) [file 13287_2015_209_MOESM2_ESM.tiff]

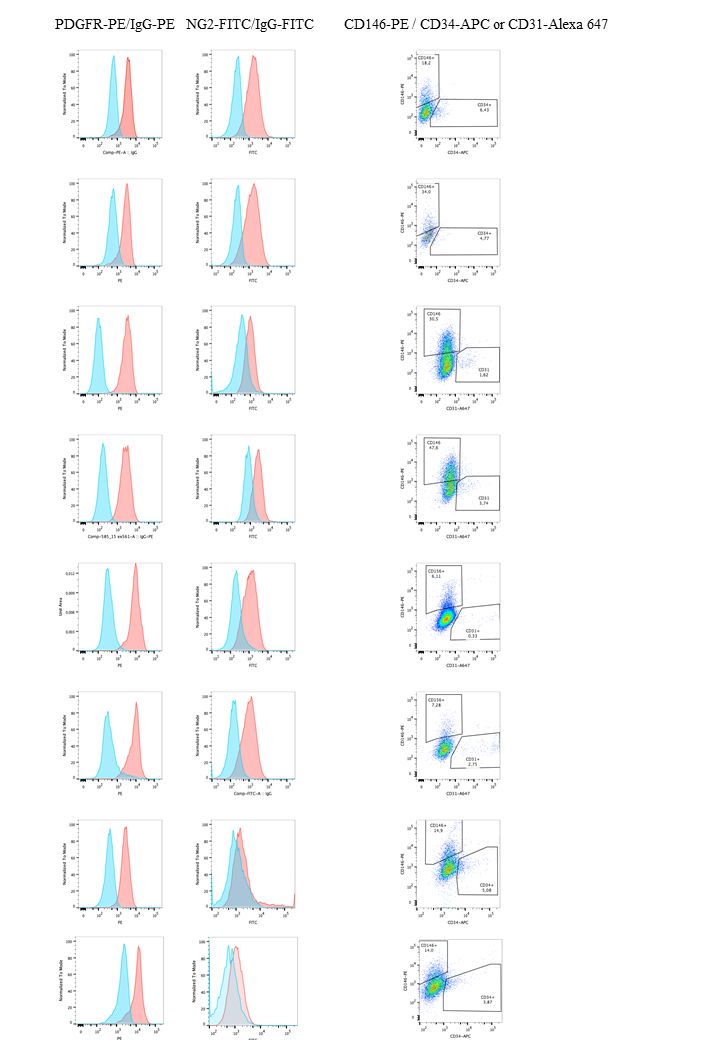

Supplement: Additional file 3: Figure S2. — Representative flow cytometry charts and plots of additional surface markers expression on ADSC obtained from eight donors (#2-9). A. - Expression of pericyte markers, PDGFRβ and NG2 glycoprotein. Pink areas – specific antibodies, blue areas – isotype antibodies. B. – Expression of CD146 together with CD34 or CD31. (TIFF 262 kb) [file 13287_2015_209_MOESM3_ESM.tiff]

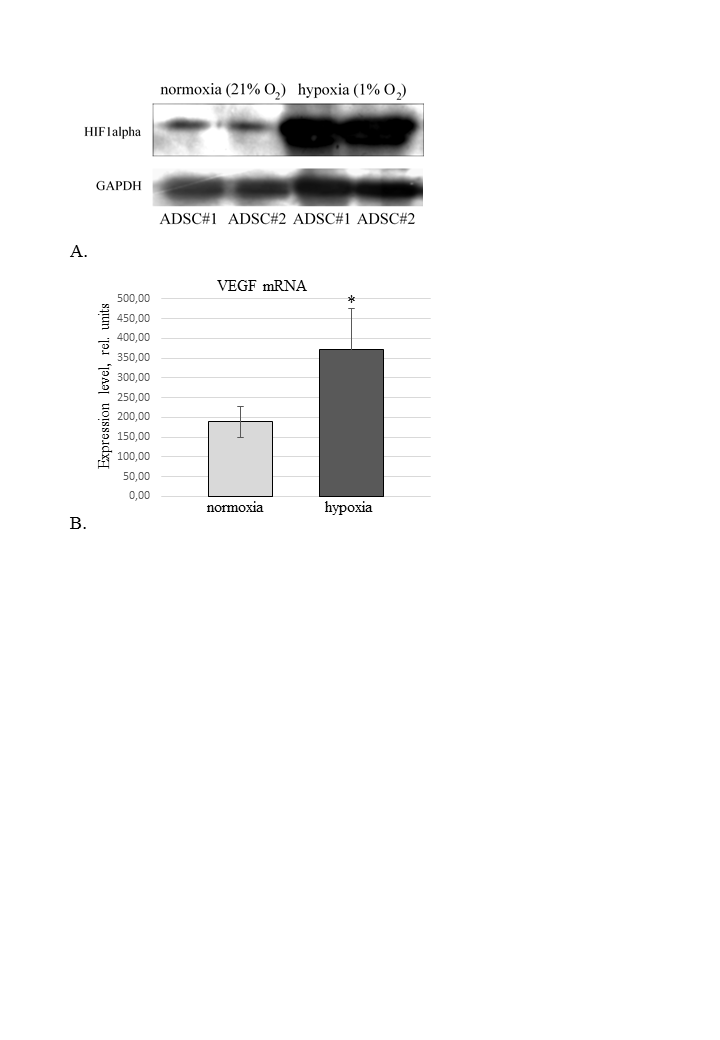

Supplement: Additional file 5: Figure S3. — ADSC response to hypoxia. A. – Representative Western blots showing the accumulation of HIF-1alpha after the incubation at 1 % O2 during 48 h. B. – Real time PCR analysis of VEGF mRNA in hypoxic and normoxic ADSCs (n = 20, * - p <0.05 vs. normoxia). (TIFF 100 kb) [file 13287_2015_209_MOESM5_ESM.tiff]
