# Supplementary material for: Characterization of secretomes provides evidence for adipose-derived mesenchymal stromal cells subtypes
Source: Stem Cell Res Ther. 2015 Nov 11;6:221. doi: 10.1186/s13287-015-0209-8 (PMC4642680; doi:10.1186/s13287-015-0209-8)
Supplement: Additional file 4: Table S3. — Data of transcriptome profiling of normoxic ADSCs. (DOCX 87 kb) [file 13287_2015_209_MOESM4_ESM.docx]

Table S3. Data of transcriptome profiling of normoxic ADSCs

| Uniprot acc # | Name | Mean signal | Signal SD | p value <0.05 | TRANSCRIPT | REFSEQ_ID |
| --- | --- | --- | --- | --- | --- | --- |
| P21589 | 5'-nucleotidase, ecto (CD73) | 231,5 | 70,7 | yes | ILMN_28610 | NM_002526.1 |
| O15144 | actin related protein 2/3 complex, subunit 2, 34kDa | 3662,8 | 515,0 | yes | ILMN_1434 | NM_005731.2 |
| O15511 | actin related protein 2/3 complex, subunit 5, 16kDa | 725,8 | 265,3 | yes | ILMN_13489 | NM_005717.2 |
| P60709 | actin, beta | 10459,3 | 5412,2 | yes | ILMN_2565 | NM_001101.2 |
| P12814 | actinin, alpha 1 | 4109,5 | 1834,4 | yes | ILMN_28160 | NM_001102.2 |
| O43707 | actinin, alpha 4 | 548,6 | 147,4 | yes | ILMN_2269 | NM_004924.3 |
| O14672 | ADAM metallopeptidase domain 10 | 51,5 | 8,5 | no | ILMN_169204 | NM_001110.2 |
| O43184 | ADAM metallopeptidase domain 12 | 70,9 | 9,2 | yes | ILMN_29338 | NM_021641.2 |
| Q13444 | ADAM metallopeptidase domain 15 | 129,4 | 33,0 | yes | ILMN_23734 | NM_003815.3 |
| P78536 | ADAM metallopeptidase domain 17 | 152,0 | 32,8 | yes | ILMN_165100 | NM_003183.4 |
| Q13443 | ADAM metallopeptidase domain 9 (meltrin gamma) | 143,8 | 32,0 | yes | ILMN_922 | NM_003816.2 |
| Q9UHL4 | ADAM metallopeptidase with thrombospondin type 1 motif, 1 | 148,1 | 89,6 | yes | ILMN_11081 | NM_006988.3 |
| O95450 | ADAM metallopeptidase with thrombospondin type 1 motif, 2 | 132,4 | 26,7 | yes | ILMN_29307 | NM_014244.2 |
| Q8N6G6 | ADAMTS-like 1 | 131,1 | 30,5 | yes | ILMN_7160 | NM_052866.3 |
| O95782 | adaptor-related protein complex 2, alpha 1 subunit | 88,2 | 15,7 | yes | ILMN_16566 | NM_130787.2 |
| P55263 | adenosine kinase | 353,8 | 61,2 | yes | ILMN_4107 | NM_001123.2 |
| Q8IUX7 | AE binding protein 1 | 2053,5 | 1232,8 | yes | ILMN_179474 | NM_001129.3 |
| P16112 | aggrecan | 48,5 | 5,2 | no | ILMN_182189 | NM_013227.2 |
| O00468 | agrin | 47,7 | 3,2 | no | ILMN_29539 | NM_198576.2 |
| P02768 | albumin | 44,7 | 2,4 | no | ILMN_28973 | NM_000477.3 |
| P04075 | aldolase A, fructose-bisphosphate | 53,1 | 2,3 | no | ILMN_20463 | NM_000034.2 |
| P02765 | alpha-2-HS-glycoprotein | 49,0 | 6,7 | no | ILMN_20116 | NM_001622.1 |
| P01023 | alpha-2-macroglobulin | 59,0 | 16,5 | yes | ILMN_8023 | NM_000014.4 |
| P05067 | amyloid beta (A4) precursor protein | 2159,7 | 322,4 | yes | ILMN_23272 | NM_201414.1 |
| Q06481 | amyloid beta (A4) precursor-like protein 2 | 61,1 | 6,7 | yes | ILMN_19935 | NM_001642.1 |
| Q9UKZ9 | angiopoietin-like 2 | 1812,8 | 1078,3 | yes | ILMN_26679 | NM_012098.2 |
| Q9GZM7 | angiopoietin-like 4 | 45,1 | 3,3 | no | ILMN_20440 | NM_139314.1 |
| P12821 | angiotensin I converting enzyme (peptidyl-dipeptidase A) 1 | 45,1 | 4,2 | no | ILMN_27028 | NM_152831.1 |
| P01019 | angiotensinogen | 113,7 | 91,1 | yes | ILMN_1261 | NM_000029.2 |
| P04083 | annexin A1 | 3811,0 | 681,1 | yes | ILMN_14184 | NM_000700.1 |
| P07355 | annexin A2 | 97,5 | 28,9 | yes | ILMN_6148 | NR_001446.2 |
| P09525 | annexin A4 | 56,5 | 4,6 | yes | ILMN_23425 | NM_001153.2 |
| P08758 | annexin A5 | 9196,6 | 1543,0 | yes | ILMN_26606 | NM_001154.2 |
| P08133 | annexin A6 | 133,2 | 27,8 | yes | ILMN_3910 | NM_004033.2 |
| P20073 | annexin A7 | 45,2 | 5,9 | no | ILMN_14787 | NM_004034.1 |
| Q9H772 | anthrax toxin receptor 1 | 1229,9 | 398,5 | yes | ILMN_14947 | NM_032208.1 |
| Q8NCW5 | apolipoprotein A-I binding protein | 744,0 | 71,4 | yes | ILMN_24882 | NM_144772.1 |
| P15289 | arylsulfatase A | 99,0 | 23,7 | yes | ILMN_17279 | NM_000487.3 |
| P15848 | arylsulfatase B | 48,0 | 2,9 | no | ILMN_27054 | NM_198709.1 |
| P20933 | aspartylglucosaminidase | 108,9 | 8,7 | yes | ILMN_165746 | NM_000027.2 |
| P53396 | ATP citrate lyase | 1932,8 | 723,7 | yes | ILMN_1374 | NM_198830.1 |
| Q15904 | ATPase, H+ transporting, lysosomal accessory protein 1 | 2641,0 | 695,6 | yes | ILMN_42218 | NM_001183.4 |
| O75787 | ATPase, H+ transporting, lysosomal accessory protein 2 | 1166,2 | 320,8 | yes | ILMN_2566 | NM_005765.2 |
| P30530 | AXL receptor tyrosine kinase | 910,8 | 203,8 | yes | ILMN_28087 | NM_021913.2 |
| P35613 | basigin (Ok blood group) | 2289,6 | 593,0 | yes | ILMN_21413 | NM_198590.1 |
| Q07812 | BCL2-associated X protein | 1718,3 | 315,3 | yes | ILMN_11763 | NM_138765.2 |
| P61769 | beta-2-microglobulin | 4312,6 | 961,2 | yes | ILMN_19648 | NM_004048.2 |
| P21810 | biglycan | 19619,7 | 1979,6 | yes | ILMN_6146 | NM_001711.3 |
| P30043 | biliverdin reductase B (flavin reductase (NADPH)) | 107,1 | 33,2 | yes | ILMN_11803 | NM_000713.1 |
| P43251 | biotinidase | 123,5 | 31,4 | yes | ILMN_24816 | NM_000060.2 |
| Q10588 | bone marrow stromal cell antigen 1 | 111,9 | 21,9 | yes | ILMN_26226 | NM_004334.1 |
| P13497 | bone morphogenetic protein 1 | 491,4 | 102,7 | yes | ILMN_6483 | NM_006129.2 |
| P80723 | brain abundant, membrane attached signal protein 1 | 4799,7 | 1386,2 | yes | ILMN_28962 | NM_006317.3 |
| Q9BXX0 | C1q and tumor necrosis factor related protein 5 | 1317,9 | 578,2 | yes | ILMN_23052 | NM_015645.2 |
| P55287 | cadherin 11, type 2, OB-cadherin (osteoblast) | 1447,8 | 591,6 | yes | ILMN_11789 | NM_001797.2 |
| P55290 | cadherin 13, H-cadherin (heart) | 267,8 | 100,6 | yes | ILMN_26240 | NM_001257.3 |
| P19022 | cadherin 2, type 1, N-cadherin (neuronal) | 2323,3 | 1613,2 | yes | ILMN_28694 | NM_001792.2 |
| P27797 | calreticulin | 342,8 | 134,2 | yes | ILMN_18909 | NM_004343.2 |
| O94985 | calsyntenin 1 | 1831,4 | 655,3 | yes | ILMN_29098 | NM_001009566.1 |
| O43852 | calumenin | 1762,9 | 588,0 | yes | ILMN_24697 | NM_001219.2 |
| Q8N129 | canopy 4 homolog (zebrafish) | 98,3 | 17,7 | yes | ILMN_15383 | NM_152755.1 |
| Q01518 | CAP, adenylate cyclase-associated protein 1 (yeast) | 2319,4 | 967,1 | yes | ILMN_175047 | NM_006367.2 |
| P47756 | capping protein (actin filament) muscle Z-line, beta | 682,5 | 115,5 | yes | ILMN_139201 | NM_004930.1 |
| P40121 | capping protein (actin filament), gelsolin-like | 81,0 | 16,9 | yes | ILMN_2712 | NM_001747.2 |
| O43570 | carbonic anhydrase XII | 1412,2 | 1053,0 | yes | ILMN_796 | NM_001218.3 |
| P16152 | carbonyl reductase 1 | 179,7 | 18,3 | yes | ILMN_18628 | NM_001757.2 |
| O75828 | carbonyl reductase 3 | 697,9 | 505,5 | yes | ILMN_8256 | NM_001236.3 |
| Q9UJ70 | carboxypeptidase A4 | 91,8 | 53,6 | yes | ILMN_164642 | NM_016352.2 |
| P16870 | carboxypeptidase E | 91,8 | 53,6 | yes | ILMN_164642 | NM_016352.2 |
| O75718 | cartilage associated protein | 2051,3 | 175,9 | yes | ILMN_180917 | NM_006371.3 |
| P49747 | cartilage oligomeric matrix protein | 1384,8 | 1174,0 | yes | ILMN_15049 | NM_000095.2 |
| P21964 | catechol-O-methyltransferase | 4056,5 | 424,8 | yes | ILMN_26337 | NM_007310.1 |
| P10619 | cathepsin A | 1725,3 | 356,3 | yes | ILMN_10174 | NM_000308.2 |
| P07858 | cathepsin B | 4263,5 | 843,9 | yes | ILMN_6129 | NM_001908.3 |
| P53634 | cathepsin C | 167,8 | 42,0 | yes | ILMN_14007 | NM_001814.2 |
| P07339 | cathepsin D | 2008,3 | 641,1 | yes | ILMN_15639 | NM_001909.3 |
| Q9UBX5 | cathepsin F | 791,8 | 202,0 | yes | ILMN_138148 | NM_003793.2 |
| P43235 | cathepsin K | 10082,7 | 2432,8 | yes | ILMN_16923 | NM_000396.2 |
| P07711 | cathepsin L1 | 5920,4 | 2089,8 | yes | ILMN_23619 | NM_001912.3 |
| P25774 | cathepsin S | 64,5 | 24,4 | no | ILMN_178498 | NM_004079.3 |
| Q9UBS4 | cathepsin Z | 561,8 | 240,5 | yes | ILMN_23976 | NM_001336.2 |
| Q6YHK3 | CD109 molecule | 88,5 | 24,9 | yes | ILMN_166918 | NM_133493.2 |
| P08571 | CD14 molecule | 368,7 | 201,6 | yes | ILMN_175125 | NM_001040021.1 |
| Q9NP84 | CD248 molecule, endosialin | 6434,3 | 1648,0 | yes | ILMN_25766 | NM_020404.2 |
| P16070 | CD44 molecule (Indian blood group) | 3131,1 | 1169,0 | yes | ILMN_10947 | NM_001001391.1 |
| P08174 | CD55 molecule | 127,5 | 19,6 | yes | ILMN_18297 | NM_000574.2 |
| P13987 | CD59 molecule, complement regulatory protein | 696,1 | 344,6 | yes | ILMN_27805 | NM_000611.4 |
| P60033 | CD81 molecule | 20847,4 | 2798,1 | yes | ILMN_29556 | NM_004356.3 |
| P14209 | CD99 molecule | 942,7 | 242,5 | yes | ILMN_9657 | NM_002414.3 |
| Q16543 | cell division cycle 37 homolog (S. cerevisiae) | 5524,3 | 538,5 | yes | ILMN_19197 | NM_007065.3 |
| Q99674 | cell growth regulator with EF-hand domain 1 | 55,6 | 6,5 | no | ILMN_11483 | NM_006569.3 |
| O75629 | cellular repressor of E1A-stimulated genes 1 | 1526,3 | 435,6 | yes | ILMN_11645 | NM_003851.2 |
| O75503 | ceroid-lipofuscinosis, neuronal 5 | 334,5 | 43,8 | yes | ILMN_23994 | NM_006493.1 |
| P09341 | chemokine (C-X-C motif) ligand 1 | 44,9 | 5,8 | no | ILMN_17166 | NM_001511.1 |
| P19876 | chemokine (C-X-C motif) ligand 3 | 48,8 | 5,8 | no | ILMN_26666 | NM_002090.2 |
| P42830 | chemokine (C-X-C motif) ligand 5 | 66,6 | 10,8 | yes | ILMN_19896 | NM_032966.1 |
| P36222 | chitinase 3-like 1 (cartilage glycoprotein-39) | 49,0 | 5,1 | no | ILMN_23515 | NM_001276.1 |
| Q9BWS9 | chitinase domain containing 1 | 47,7 | 2,7 | no | ILMN_19377 | NM_023947.2 |
| Q01459 | chitobiase, di-N-acetyl- | 171,3 | 17,5 | yes | ILMN_14531 | NM_004388.1 |
| O00299 | chloride intracellular channel 1 | 2136,8 | 593,1 | yes | ILMN_29900 | NM_001288.4 |
| Q9Y696 | chloride intracellular channel 4 | 898,6 | 310,9 | yes | ILMN_3174 | NM_013943.1 |
| Q6UVK1 | chondroitin sulfate proteoglycan 4 | 69,1 | 12,9 | yes | ILMN_1596 | NM_001897.4 |
| Q9HCB6 | chromosome 12 open reading frame 10 | 872,6 | 238,0 | yes | ILMN_29960 | NM_021640.3 |
| Q969H8 | chromosome 19 open reading frame 10 | 641,9 | 199,3 | yes | ILMN_14819 | NM_019107.3 |
| P10909 | clusterin | 49,5 | 5,0 | no | ILMN_181193 | NM_203339.1 |
| Q96KP4 | CNDP dipeptidase 2 (metallopeptidase M20 family) | 1595,3 | 389,3 | yes | ILMN_4139 | NM_018235.1 |
| Q14019 | coactosin-like 1 (Dictyostelium) | 50,1 | 5,6 | no | ILMN_3414 | NM_021149.2 |
| P12259 | coagulation factor V (proaccelerin, labile factor) | 59,7 | 10,1 | yes | ILMN_174093 | NM_000130.4 |
| Q76M96 | coiled-coil domain containing 80 | 276,5 | 218,6 | yes | ILMN_164623 | NM_199511.1 |
| Q96CG8 | collagen triple helix repeat containing 1 | 3762,3 | 1014,0 | yes | ILMN_7282 | NM_138455.2 |
| P02452 | collagen, type I, alpha 1 | 16991,4 | 3303,4 | yes | ILMN_11548 | NM_000088.3 |
| P08123 | collagen, type I, alpha 2 | 17807,4 | 1311,4 | yes | ILMN_23060 | NM_000089.3 |
| P02461 | collagen, type III, alpha 1 | 2978,8 | 551,3 | yes | ILMN_182795 | NM_000090.3 |
| P02462 | collagen, type IV, alpha 1 | 837,6 | 326,1 | yes | ILMN_24359 | NM_001845.4 |
| P08572 | collagen, type IV, alpha 2 | 183,2 | 74,3 | yes | ILMN_179856 | NM_001846.2 |
| P20908 | collagen, type V, alpha 1 | 4483,4 | 1196,0 | yes | ILMN_31902 | NM_000093.3 |
| P05997 | collagen, type V, alpha 2 | 1968,9 | 584,9 | yes | ILMN_42782 | NM_000393.3 |
| P12109 | collagen, type VI, alpha 1 | 4358,0 | 1220,8 | yes | ILMN_179738 | NM_001848.2 |
| P12110 | collagen, type VI, alpha 2 | 3702,0 | 703,3 | yes | ILMN_21525 | NM_001849.3 |
| P12111 | collagen, type VI, alpha 3 | 8197,1 | 1381,0 | yes | ILMN_29206 | NM_057165.2 |
| Q02388 | collagen, type VII, alpha 1 | 213,9 | 45,6 | yes | ILMN_24830 | NM_000094.2 |
| P27658 | collagen, type VIII, alpha 1 | 2308,8 | 1968,9 | yes | ILMN_10408 | NM_020351.2 |
| P12107 | collagen, type XI, alpha 1 | 358,6 | 202,1 | yes | ILMN_3994 | NM_001854.3 |
| Q99715 | collagen, type XII, alpha 1 | 3325,8 | 1350,5 | yes | ILMN_12229 | NM_080645.2 |
| Q05707 | collagen, type XIV, alpha 1 | 147,6 | 52,8 | yes | ILMN_175659 | NM_021110.1 |
| P39059 | collagen, type XV, alpha 1 | 535,2 | 255,9 | yes | ILMN_178882 | NM_001855.3 |
| Q07092 | collagen, type XVI, alpha 1 | 1544,1 | 506,6 | yes | ILMN_171740 | NM_001856.3 |
| P39060 | collagen, type XVIII, alpha 1 | 312,6 | 130,5 | yes | ILMN_13022 | NM_130445.2 |
| P09603 | colony stimulating factor 1 (macrophage) | 72,8 | 13,3 | yes | ILMN_19274 | NM_172212.1 |
| P00736 | complement component 1, r subcomponent | 1940,1 | 644,5 | yes | ILMN_19834 | NM_001733.4 |
| P09871 | complement component 1, s subcomponent | 3694,2 | 1311,4 | yes | ILMN_29321 | NM_001734.2 |
| P0C0L4 | complement component 4A (Rodgers blood group) | 46,5 | 3,6 | no | ILMN_10388 | NM_007293.2 |
| P0C0L5 | complement component 4B (Chido blood group) | 149,2 | 43,1 | yes | ILMN_11559 | NM_000592.4 |
| P00751 | complement factor D (adipsin) | 151,0 | 23,5 | yes | ILMN_29029 | NM_001928.2 |
| P00746 | complement factor H | 153,8 | 22,5 | yes | ILMN_12111 | NM_001014975.1 |
| P08603 | connective tissue growth factor | 4366,9 | 3505,1 | yes | ILMN_3374 | NM_001901.2 |
| P29279 | C-type lectin domain family 11, member A | 461,8 | 134,8 | yes | ILMN_29894 | NM_002975.2 |
| Q9Y263 | C-type lectin domain family 3, member B | 130,4 | 137,7 | yes | ILMN_1287 | NM_003278.1 |
| P05452 | cutA divalent cation tolerance homolog (E. coli) | 3942,1 | 878,2 | yes | ILMN_6058 | NM_001014838.1 |
| O60888 | cystatin C | 8150,6 | 929,5 | yes | ILMN_15664 | NM_000099.2 |
| P01034 | cystatin SN | 46,8 | 6,3 | no | ILMN_14080 | NM_001898.2 |
| P01037 | cysteine and glycine-rich protein 1 | 1056,5 | 484,3 | yes | ILMN_25451 | NM_004078.1 |
| P21291 | cysteine rich transmembrane BMP regulator 1 (chordin-like) | 491,6 | 202,4 | yes | ILMN_10588 | NM_016441.1 |
| Q9P0L0 | cysteine-rich, angiogenic inducer, 61 | 5050,5 | 2833,3 | yes | ILMN_21983 | NM_001554.3 |
| O00622 | cytidine monophosphate (UMP-CMP) kinase 1, cytosolic | 144,5 | 30,8 | yes | ILMN_12452 | NM_016308.1 |
| P30085 | cytokine receptor-like factor 1 | 192,1 | 76,8 | yes | ILMN_4898 | NM_004750.2 |
| O75462 | damage-specific DNA binding protein 1, 127kDa | 4183,3 | 738,3 | yes | ILMN_27784 | NM_001923.3 |
| Q16531 | decorin | 20262,9 | 4768,8 | yes | ILMN_29913 | NM_133505.2 |
| P07585 | deoxyribonuclease II, lysosomal | 1618,4 | 165,7 | yes | ILMN_27899 | NM_001375.2 |
| O00115 | deoxyuridine triphosphatase | 1618,4 | 165,7 | yes | ILMN_27899 | NM_001375.2 |
| P33316 | dermatopontin | 290,2 | 238,1 | yes | ILMN_23209 | NM_001937.3 |
| Q07507 | dickkopf homolog 1 (Xenopus laevis) | 2273,0 | 880,7 | yes | ILMN_22862 | NM_012242.2 |
| O94907 | dickkopf homolog 3 (Xenopus laevis) | 1215,2 | 652,0 | yes | ILMN_4127 | NM_013253.4 |
| Q9UBR2 | dimethylarginine dimethylaminohydrolase 1 | 261,3 | 169,4 | yes | ILMN_182204 | NM_012137.2 |
| O94760 | dipeptidyl-peptidase 3 | 184,0 | 31,6 | yes | ILMN_37188 | NM_005700.3 |
| Q9NYU2 | dipeptidyl-peptidase 4 | 159,5 | 43,5 | yes | ILMN_166089 | NM_001935.3 |
| P27487 | dipeptidyl-peptidase 7 | 1085,1 | 343,9 | yes | ILMN_6361 | NM_013379.2 |
| Q9UI42 | discoidin, CUB and LCCL domain containing 2 | 814,6 | 297,6 | yes | ILMN_175741 | NM_080927.3 |
| Q96PD2 | DnaJ (Hsp40) homolog, subfamily B, member 11 | 943,9 | 335,9 | yes | ILMN_14179 | NM_016306.4 |
| Q9UBX1 | DnaJ (Hsp40) homolog, subfamily C, member 3 | 60,2 | 8,4 | yes | ILMN_26093 | NM_006260.2 |
| Q13217 | drebrin-like | 1287,6 | 327,2 | yes | ILMN_26610 | NM_001014436.1 |
| Q9UKK9 | dynactin 2 (p50) | 2805,3 | 449,1 | yes | ILMN_11887 | NM_006400.3 |
| Q13561 | dystroglycan 1 (dystrophin-associated glycoprotein 1) | 613,8 | 217,8 | yes | ILMN_16432 | NM_004393.2 |
| Q14118 | early endosome antigen 1 | 164,1 | 22,2 | yes | ILMN_2943 | NM_003566.2 |
| Q15075 | ectonucleotide pyrophosphatase/phosphodiesterase 2 | 1326,4 | 687,8 | yes | ILMN_177018 | NM_001040092.1 |
| Q13822 | EGF-containing fibulin-like extracellular matrix protein 1 | 9542,5 | 2571,6 | yes | ILMN_41104 | NM_001039348.1 |
| Q12805 | EGF-containing fibulin-like extracellular matrix protein 2 | 2582,5 | 430,8 | yes | ILMN_42286 | NM_016938.2 |
| O95967 | elastin | 233,1 | 157,4 | yes | ILMN_167035 | NM_001081754.1 |
| P15502 | elastin microfibril interfacer 1 | 411,5 | 54,6 | yes | ILMN_18157 | NM_007046.1 |
| Q9Y6C2 | elastin microfibril interfacer 2 | 624,7 | 250,4 | yes | ILMN_1610 | NM_032048.2 |
| Q9BY76 | endoglin | 477,6 | 113,5 | yes | ILMN_18184 | NM_000118.1 |
| P17813 | endoplasmic reticulum aminopeptidase 1 | 68,7 | 6,6 | yes | ILMN_177321 | NM_001040458.1 |
| Q9NZN4 | endoplasmic reticulum protein 29 | 5132,3 | 1033,8 | yes | ILMN_8686 | NM_001034025.1 |
| P30040 | endothelial cell-specific molecule 1 | 61,6 | 13,9 | yes | ILMN_168618 | NM_007036.3 |
| Q9NQC3 | ependymin related protein 1 (zebrafish) | 1773,0 | 731,9 | yes | ILMN_170238 | NM_017549.3 |
| Q9UMX5 | epidermal growth factor receptor (erythroblastic leukemia viral (v-erb-b) oncogene homolog, avian) | 226,0 | 54,6 | yes | ILMN_164647 | NM_005228.3 |
| P00533 | epidermal growth factor receptor pathway substrate 8 | 130,8 | 28,9 | yes | ILMN_17717 | NM_004447.4 |
| Q12929 | ERO1-like (S. cerevisiae) | 173,3 | 23,1 | yes | ILMN_4958 | NM_014584.1 |
| Q96HE7 | exostoses (multiple) 2 | 383,9 | 100,6 | yes | ILMN_16578 | NM_000401.2 |
| Q93063 | extracellular matrix protein 1 | 393,7 | 74,1 | yes | ILMN_6103 | NM_004425.2 |
| Q16610 | extracellular matrix protein 2, female organ and adipocyte specific | 298,4 | 118,0 | yes | ILMN_7147 | NM_001393.2 |
| O94769 | family with sequence similarity 20, member B | 391,0 | 138,9 | yes | ILMN_9442 | NM_014864.2 |
| O75063 | family with sequence similarity 20, member C | 702,2 | 174,8 | yes | ILMN_5460 | NM_020223.2 |
| Q8IXL6 | family with sequence similarity 3, member C | 291,7 | 78,2 | yes | ILMN_180739 | NM_001040020.1 |
| Q92520 | fascin homolog 1, actin-bundling protein | 3752,0 | 1189,8 | yes | ILMN_177947 | NM_003088.2 |
| Q16658 | fatty acid synthase | 8164,0 | 3905,4 | yes | ILMN_16128 | NM_004104.4 |
| P49327 | fibrillin 1 | 149,7 | 39,2 | yes | ILMN_4226 | NM_000138.3 |
| P35555 | fibrillin 2 | 975,1 | 828,3 | yes | ILMN_22082 | NM_001999.3 |
| P35556 | fibroblast growth factor receptor 1 | 104,3 | 11,2 | yes | ILMN_9604 | NM_023107.2 |
| P11362 | fibromodulin | 345,2 | 184,0 | yes | ILMN_29801 | NM_002023.3 |
| Q06828 | fibronectin 1 | 233,5 | 82,1 | yes | ILMN_20090 | NM_002026.2 |
| P02751 | fibronectin type III domain containing 1 | 1391,7 | 628,2 | yes | ILMN_17340 | NM_032532.2 |
| Q4ZHG4 | fibulin 1 | 12459,6 | 3277,9 | yes | ILMN_12209 | NM_006486.2 |
| P23142 | fibulin 2 | 3046,3 | 1280,3 | yes | ILMN_29279 | NM_001998.2 |
| P98095 | fibulin 5 | 958,8 | 326,7 | yes | ILMN_29187 | NM_006329.2 |
| Q9UHB6 | filamin A, alpha (actin binding protein 280) | 63,6 | 10,7 | yes | ILMN_178124 | NM_001456.2 |
| P21333 | filamin B, beta (actin binding protein 278) | 811,9 | 318,2 | yes | ILMN_21371 | NM_001457.1 |
| O75369 | filamin C, gamma (actin binding protein 280) | 495,7 | 246,2 | yes | ILMN_167330 | NM_001458.3 |
| Q14315 | fin bud initiation factor homolog (zebrafish) | 67,2 | 15,7 | yes | ILMN_173664 | NM_203371.1 |
| Q8TAL6 | FK506 binding protein 10, 65 kDa | 103,1 | 33,7 | yes | ILMN_8804 | NM_021939.2 |
| Q96AY3 | FK506 binding protein 2, 13kDa | 381,2 | 51,0 | yes | ILMN_17464 | NM_004470.2 |
| P26885 | FK506 binding protein 4, 59kDa | 139,1 | 15,3 | yes | ILMN_9429 | NM_002014.2 |
| Q02790 | FK506 binding protein 7 | 62,2 | 3,7 | yes | ILMN_5958 | NM_181342.1 |
| Q9Y680 | FK506 binding protein 9, 63 kDa | 119,4 | 33,8 | yes | ILMN_27143 | NM_007270.2 |
| O95302 | follistatin | 2471,5 | 831,0 | yes | ILMN_11736 | NM_013409.1 |
| P19883 | follistatin-like 1 | 1228,4 | 503,4 | yes | ILMN_15510 | NM_007085.3 |
| Q12841 | follistatin-like 3 (secreted glycoprotein) | 169,9 | 95,7 | yes | ILMN_13221 | NM_005860.2 |
| O95633 | fucosidase, alpha-L- 1, tissue | 181,8 | 39,6 | yes | ILMN_14069 | NM_000147.3 |
| P04066 | fucosidase, alpha-L- 2, plasma | 344,8 | 41,5 | yes | ILMN_13837 | NM_032020.3 |
| Q9BTY2 | fusion (involved in t(12;16) in malignant liposarcoma) | 80,9 | 17,8 | yes | ILMN_22748 | NM_004960.2 |
| P35637 | galactosamine (N-acetyl)-6-sulfate sulfatase | 59,1 | 6,8 | no | ILMN_8615 | NM_000512.3 |
| P34059 | galactosidase, beta 1 | 2082,4 | 424,9 | yes | ILMN_173609 | NM_001079811.1 |
| P16278 | galactosylceramidase | 321,5 | 59,6 | yes | ILMN_28156 | NM_000153.2 |
| P54803 | gamma-glutamyl hydrolase | 178,6 | 40,8 | yes | ILMN_9870 | NM_003878.1 |
| Q92820 | gelsolin (amyloidosis, Finnish type) | 555,9 | 79,2 | yes | ILMN_46419 | NM_198252.2 |
| P06396 | glucosamine (N-acetyl)-6-sulfatase | 9058,5 | 1104,0 | yes | ILMN_177670 | NM_002076.2 |
| P15586 | glucose-6-phosphate dehydrogenase | 1757,6 | 566,7 | yes | ILMN_162326 | NM_000402.3 |
| P11413 | glucosidase, alpha; acid | 298,9 | 32,8 | yes | ILMN_182951 | NM_001079804.1 |
| P10253 | glucosidase, alpha; neutral AB | 156,3 | 43,9 | yes | ILMN_29263 | NM_198334.1 |
| Q14697 | glucosidase, beta; acid (includes glucosylceramidase) | 1211,8 | 244,3 | yes | ILMN_28933 | NM_001005742.1 |
| P04062 | glutaminyl-peptide cyclotransferase | 439,9 | 163,9 | yes | ILMN_6510 | NM_012413.3 |
| Q16769 | glutathione S-transferase omega 1 | 2363,8 | 366,4 | yes | ILMN_17696 | NM_004832.1 |
| P78417 | glutathione S-transferase pi 1 | 3890,3 | 248,3 | yes | ILMN_10475 | NM_000852.2 |
| P09211 | glycoprotein (transmembrane) nmb | 4391,9 | 944,2 | yes | ILMN_20483 | NM_001005340.1 |
| Q14956 | glycyl-tRNA synthetase | 2756,8 | 1258,3 | yes | ILMN_174609 | NM_002047.2 |
| P41250 | glypican 1 | 233,7 | 52,1 | yes | ILMN_15161 | NM_002081.1 |
| P35052 | glypican 6 | 187,2 | 38,5 | yes | ILMN_16550 | NM_005708.2 |
| Q9Y646 | GM2 ganglioside activator | 118,3 | 64,1 | yes | ILMN_8836 | NM_000405.3 |
| P17900 | golgi apparatus protein 1 | 2513,6 | 300,4 | yes | ILMN_29553 | NM_012201.4 |
| Q92896 | golgi membrane protein 1 | 178,0 | 8,2 | yes | ILMN_16902 | NM_177937.1 |
| Q8NBJ4 | granulin | 2840,6 | 555,4 | yes | ILMN_18655 | NM_002087.2 |
| P28799 | gremlin 1 | 1556,5 | 867,8 | yes | ILMN_16591 | NM_013372.5 |
| O60565 | gremlin 2 | 92,4 | 25,0 | yes | ILMN_24616 | NM_022469.3 |
| Q9HAT2 | growth arrest-specific 1 | 4832,3 | 629,2 | yes | ILMN_175833 | NM_002048.1 |
| P54826 | growth differentiation factor 15 | 1847,8 | 503,0 | yes | ILMN_2688 | NM_004864.1 |
| Q99988 | heat shock 27kDa protein-like 2 pseudogene; heat shock 27kDa protein 1 | 3542,7 | 464,9 | yes | ILMN_28967 | NM_001540.2 |
| P04792 | heat shock protein 90kDa beta (Grp94), member 1 | 2982,0 | 1169,2 | yes | ILMN_27563 | NM_003299.1 |
| P14625 | HEG homolog 1 (zebrafish) | 297,8 | 176,4 | yes | ILMN_306841 | NM_020733.1 |
| Q9UM22 | heme binding protein 1 | 658,0 | 190,9 | yes | ILMN_4128 | NM_015987.3 |
| Q9NS15 | heme binding protein 2 | 409,7 | 176,7 | yes | ILMN_21130 | NM_014320.2 |
| Q9Y625 | hemicentin 1 | 50,3 | 5,5 | yes | ILMN_170646 | NM_031935.2 |
| Q96RW7 | hemoglobin, beta | 49,8 | 1,2 | no | ILMN_28875 | NM_000518.4 |
| P68871 | heparan sulfate proteoglycan 2 | 76,6 | 16,6 | yes | ILMN_734 | NM_005529.5 |
| P98160 | heterogeneous nuclear ribonucleoprotein D-like | 359,8 | 30,4 | yes | ILMN_179128 | NR_003249.1 |
| O14979 | hexosaminidase A (alpha polypeptide) | 193,7 | 50,5 | yes | ILMN_27287 | NM_000520.3 |
| P06865 | hexosaminidase B (beta polypeptide) | 4817,1 | 692,1 | yes | ILMN_647 | NM_000521.2 |
| P07686 | hexose-6-phosphate dehydrogenase (glucose 1-dehydrogenase) | 122,3 | 36,0 | yes | ILMN_182713 | NM_004285.3 |
| O95479 | HtrA serine peptidase 1 | 8459,7 | 985,0 | yes | ILMN_10981 | NM_002775.3 |
| Q92743 | HtrA serine peptidase 3 | 55,3 | 9,1 | no | ILMN_28233 | NM_053044.2 |
| P83110 | hyaluronan and proteoglycan link protein 1 | 113,8 | 57,4 | yes | ILMN_162137 | NM_001884.2 |
| P10915 | hyaluronan and proteoglycan link protein 3 | 108,2 | 32,2 | yes | ILMN_19816 | NM_178232.2 |
| Q96S86 | hypothetical gene supported by AF216292; NM_005347; heat shock 70kDa protein 5 (glucose-regulated protein, 78kDa) | 446,2 | 126,6 | yes | ILMN_16546 | NM_005347.2 |
| P11021 | hypothetical LOC100128526; target of myb1 (chicken) | 784,7 | 221,0 | yes | ILMN_27313 | NM_005488.1 |
| O60784 | hypothetical LOC100129500; apolipoprotein E | 2174,3 | 2366,7 | yes | ILMN_11525 | NM_000041.2 |
| P02649 | hypothetical protein LOC100133690; activated leukocyte cell adhesion molecule | 145,6 | 41,1 | yes | ILMN_164638 | NM_001627.2 |
| Q13740 | hypothetical protein LOC100133770; vacuolar protein sorting 35 homolog (S. cerevisiae) | 898,4 | 187,8 | yes | ILMN_21093 | NM_018206.3 |
| Q96QK1 | hypoxia up-regulated 1 | 165,5 | 53,6 | yes | ILMN_167916 | NM_006389.2 |
| Q9Y5X1 | iduronate 2-sulfatase | 494,5 | 149,8 | yes | ILMN_2023 | NM_000202.3 |
| P22304 | iduronidase, alpha-L- | 646,6 | 213,7 | yes | ILMN_11556 | NM_000203.3 |
| P35475 | immunoglobulin superfamily containing leucine-rich repeat | 443,6 | 152,1 | yes | ILMN_22549 | NM_005545.3 |
| O14498 | inhibin, beta A | 47,2 | 3,1 | no | ILMN_2320 | NM_002192.2 |
| P08476 | insulin-like growth factor binding protein 3 | 1833,0 | 949,3 | yes | ILMN_28010 | NM_000598.4 |
| P17936 | insulin-like growth factor binding protein 4 | 1929,5 | 365,1 | yes | ILMN_9309 | NM_001552.2 |
| P22692 | insulin-like growth factor binding protein 5 | 3062,3 | 2888,9 | yes | ILMN_168089 | NM_000599.2 |
| P24593 | insulin-like growth factor binding protein 6 | 4605,9 | 2681,1 | yes | ILMN_5216 | NM_002178.2 |
| P24592 | insulin-like growth factor binding protein 7 | 4767,1 | 1259,6 | yes | ILMN_894 | NM_001553.1 |
| Q16270 | integral membrane protein 2B | 5463,9 | 1314,1 | yes | ILMN_3199 | NM_021999.2 |
| Q9Y2B0 | integrin, alpha V (vitronectin receptor, alpha polypeptide, antigen CD51) | 719,6 | 168,6 | yes | ILMN_182431 | NM_002210.2 |
| P06756 | integrin, beta 1 (fibronectin receptor, beta polypeptide, antigen CD29 includes MDF2, MSK12) | 4879,3 | 1401,5 | yes | ILMN_178157 | NM_002211.2 |
| P05556 | integrin, beta-like 1 (with EGF-like repeat domains) | 56,7 | 7,7 | no | ILMN_22983 | NM_004791.1 |
| O95965 | inter-alpha (globulin) inhibitor H2 | 45,2 | 4,9 | no | ILMN_8972 | NM_002216.2 |
| P19823 | inter-alpha (globulin) inhibitor H3 | 44,3 | 3,3 | no | ILMN_1303 | NM_002217.3 |
| Q06033 | inter-alpha (globulin) inhibitor H5 | 8663,4 | 3109,2 | yes | ILMN_9175 | NM_001001851.1 |
| Q86UX2 | intercellular adhesion molecule 1 | 45,3 | 3,8 | no | ILMN_17682 | NM_000201.1 |
| P05362 | interferon, gamma-inducible protein 30 | 110,7 | 35,9 | yes | ILMN_23180 | NM_006332.3 |
| P13284 | interleukin 6 (interferon, beta 2) | 799,9 | 577,3 | yes | ILMN_6469 | NM_000600.1 |
| P05231 | interleukin 8 | 1881,0 | 3311,2 | yes | ILMN_179575 | NM_000584.2 |
| P10145 | KDEL (Lys-Asp-Glu-Leu) containing 2 | 271,3 | 66,2 | yes | ILMN_23677 | NM_153705.4 |
| Q7Z4H8 | KIAA1199 | 1450,1 | 677,5 | yes | ILMN_10606 | NM_018689.1 |
| Q8WUJ3 | KIT ligand | 152,8 | 56,2 | yes | ILMN_172829 | NM_000899.3 |
| P07195 | lactotransferrin | 44,3 | 3,2 | no | ILMN_20816 | NM_002343.2 |
| P02788 | lamin A/C | 2300,5 | 865,3 | yes | ILMN_12442 | NM_005572.3 |
| P02545 | laminin, alpha 1 | 196,0 | 83,7 | yes | ILMN_27530 | NM_005559.2 |
| P25391 | laminin, alpha 2 | 278,4 | 95,2 | yes | ILMN_176777 | NM_001079823.1 |
| P24043 | laminin, alpha 4 | 941,2 | 250,1 | yes | ILMN_4021 | NM_002290.2 |
| Q16363 | laminin, alpha 5 | 166,3 | 56,8 | yes | ILMN_12588 | NM_005560.3 |
| O15230 | laminin, beta 1 | 181,7 | 41,2 | yes | ILMN_182874 | NM_002291.1 |
| P07942 | laminin, beta 2 (laminin S) | 296,8 | 79,8 | yes | ILMN_177956 | NM_002292.3 |
| P55268 | laminin, gamma 1 (formerly LAMB2) | 4096,2 | 984,7 | yes | ILMN_182622 | NM_002293.2 |
| P11047 | latent transforming growth factor beta binding protein 1 | 50,5 | 8,9 | no | ILMN_17036 | NM_000627.2 |
| Q14766 | latent transforming growth factor beta binding protein 2 | 945,9 | 203,2 | yes | ILMN_918 | NM_021070.2 |
| Q14767 | layilin | 745,9 | 212,5 | yes | ILMN_15062 | NM_178834.3 |
| Q6UX15 | lectin, galactoside-binding, soluble, 3 binding protein | 993,9 | 396,3 | yes | ILMN_24091 | NM_005567.2 |
| Q08380 | lectin, mannose-binding 2 | 82,8 | 14,3 | yes | ILMN_9504 | NM_006816.1 |
| Q12907 | legumain | 1227,3 | 369,1 | yes | ILMN_183373 | NM_001008530.1 |
| Q99538 | leucine rich repeat containing 15 | 165,5 | 73,6 | yes | ILMN_28543 | NM_130830.2 |
| Q8TF66 | leucine rich repeat containing 32 | 1496,5 | 196,7 | yes | ILMN_3568 | NM_005512.1 |
| Q14392 | LIM domain and actin binding 1 | 947,1 | 327,7 | yes | ILMN_6603 | NM_016357.3 |
| Q9UHD8 | lipase A, lysosomal acid, cholesterol esterase | 2185,6 | 518,0 | yes | ILMN_17379 | NM_000235.2 |
| P38571 | low density lipoprotein receptor | 3368,3 | 1120,6 | yes | ILMN_10126 | NM_000527.2 |
| P01130 | low density lipoprotein-related protein 1 (alpha-2-macroglobulin receptor) | 138,5 | 28,1 | yes | ILMN_169590 | NM_002332.2 |
| Q07954 | lumican | 7688,2 | 2619,1 | yes | ILMN_4223 | NM_002345.3 |
| P51884 | lysosomal-associated membrane protein 1 | 8046,1 | 1317,1 | yes | ILMN_27826 | NM_005561.2 |
| P11279 | lysosomal-associated membrane protein 2 | 2398,4 | 345,2 | yes | ILMN_29225 | NM_002294.1 |
| P13473 | lysyl oxidase | 1534,4 | 585,3 | yes | ILMN_11693 | NM_002317.3 |
| P28300 | lysyl oxidase-like 1 | 245,5 | 65,0 | yes | ILMN_7655 | NM_005576.2 |
| Q08397 | lysyl oxidase-like 2 | 44,4 | 4,3 | no | ILMN_22633 | NM_002318.2 |
| Q9Y4L1 | macrophage migration inhibitory factor (glycosylation-inhibiting factor) | 1874,1 | 384,9 | yes | ILMN_26688 | NM_002415.1 |
| P14174 | major histocompatibility complex, class I, A | 9195,0 | 1861,4 | yes | ILMN_7985 | NM_002116.5 |
| P30443 | major histocompatibility complex, class I, A | 1931,8 | 381,8 | yes | ILMN_168174 | NM_001080840.1 |
| P04439 | major histocompatibility complex, class I, A | 1249,5 | 544,6 | yes | ILMN_18149 | NM_005514.5 |
| P30447 | major histocompatibility complex, class I, C; major histocompatibility complex, class I, B | 137,3 | 48,3 | yes | ILMN_23822 | NM_002117.4 |
| P30460 | major vault protein | 1710,8 | 507,7 | yes | ILMN_3769 | NM_005115.3 |
| Q14764 | mannan-binding lectin serine peptidase 1 (C4/C2 activating component of Ra-reactive factor) | 140,2 | 64,9 | yes | ILMN_9215 | NM_139125.2 |
| P48740 | mannose receptor, C type 2 | 172,0 | 39,1 | yes | ILMN_20561 | NM_006039.3 |
| Q9UBP4 | mannosidase, alpha, class 2B, member 1 | 218,2 | 56,4 | yes | ILMN_15122 | NM_000528.2 |
| O00754 | mannosidase, alpha, class 2B, member 2 | 242,6 | 52,0 | yes | ILMN_14776 | NM_015274.1 |
| Q9Y490 | mannosidase, beta A, lysosomal | 648,0 | 261,6 | yes | ILMN_2657 | NM_005908.3 |
| O00462 | matrilin 3 | 71,3 | 28,6 | yes | ILMN_165156 | NM_002381.4 |
| O15232 | matrix metallopeptidase 1 (interstitial collagenase) | 67,4 | 22,3 | yes | ILMN_26214 | NM_002421.2 |
| P03956 | matrix metallopeptidase 14 (membrane-inserted) | 236,1 | 28,4 | yes | ILMN_179693 | NM_004995.2 |
| P50281 | matrix metallopeptidase 19 | 59,3 | 4,8 | no | ILMN_7546 | NM_001032360.1 |
| Q99542 | matrix metallopeptidase 2 (gelatinase A, 72kDa gelatinase, 72kDa type IV collagenase) | 540,7 | 157,6 | yes | ILMN_19632 | NM_004530.2 |
| P08253 | matrix metallopeptidase 3 (stromelysin 1, progelatinase) | 242,2 | 188,1 | yes | ILMN_1300 | NM_002422.3 |
| P08254 | matrix metallopeptidase 9 (gelatinase B, 92kDa gelatinase, 92kDa type IV collagenase) | 574,5 | 638,7 | yes | ILMN_28136 | NM_004994.2 |
| P14780 | matrix-remodelling associated 5 | 1694,7 | 1572,7 | yes | ILMN_29922 | NM_015419.2 |
| Q9NRN5 | matrix-remodelling associated 8 | 917,1 | 294,1 | yes | ILMN_20155 | NM_032348.2 |
| Q9BRK3 | melanoma inhibitory activity family, member 3 | 115,8 | 16,4 | yes | ILMN_181799 | NM_198551.2 |
| Q5JRA6 | membrane frizzled-related protein | 45,8 | 2,2 | yes | ILMN_8121 | NM_031433.1 |
| Q9BXJ0 | mesencephalic astrocyte-derived neurotrophic factor | 1898,7 | 558,0 | yes | ILMN_28822 | NM_006010.2 |
| P55145 | meteorin, glial cell differentiation regulator-like; similar to meteorin, glial cell differentiation regulator-like | 186,5 | 55,2 | yes | ILMN_25836 | NM_024042.2 |
| Q641Q3 | microfibrillar associated protein 5 | 2784,9 | 866,5 | yes | ILMN_19364 | NM_003480.2 |
| Q13361 | microfibrillar-associated protein 2 | 441,2 | 82,4 | yes | ILMN_14313 | NM_017459.1 |
| P55001 | microfibrillar-associated protein 4 | 5867,6 | 1265,8 | yes | ILMN_20369 | NM_002404.1 |
| P55083 | microtubule-associated protein 1A | 576,6 | 173,2 | yes | ILMN_13521 | NM_002373.4 |
| P78559 | milk fat globule-EGF factor 8 protein | 12190,6 | 3656,5 | yes | ILMN_11368 | NM_005928.1 |
| Q08431 | monooxygenase, DBH-like 1 | 652,9 | 496,8 | yes | ILMN_25648 | NM_015529.2 |
| Q6UVY6 | multiple coagulation factor deficiency 2 | 276,8 | 96,1 | yes | ILMN_1677 | NM_139279.3 |
| Q8NI22 | multiple inositol polyphosphate histidine phosphatase, 1 | 62,7 | 8,4 | yes | ILMN_29353 | NM_004897.2 |
| Q9Y240 | myosin, heavy chain 10, non-muscle | 3029,1 | 1877,4 | yes | ILMN_23305 | NM_005964.1 |
| P35580 | myosin, heavy chain 9, non-muscle | 4768,4 | 1843,8 | yes | ILMN_183555 | NM_002473.3 |
| P35579 | myosin, light chain 6, alkali, smooth muscle and non-muscle | 9959,0 | 3317,9 | yes | ILMN_33047 | NM_021019.3 |
| P60660 | N-acetylgalactosaminidase, alpha- | 67,3 | 6,2 | yes | ILMN_21273 | NM_000262.1 |
| P17050 | N-acetylglucosamine kinase | 863,2 | 161,7 | yes | ILMN_4544 | NM_017567.2 |
| Q9UJJ9 | N-acetylglucosamine-1-phosphate transferase, gamma subunit | 568,3 | 39,9 | yes | ILMN_28173 | NM_032520.3 |
| P54802 | N-acylsphingosine amidohydrolase (acid ceramidase) 1 | 136,7 | 26,0 | yes | ILMN_162236 | NM_000263.3 |
| Q13510 | nascent polypeptide-associated complex alpha subunit | 5631,6 | 998,4 | yes | ILMN_17162 | NM_005594.2 |
| Q13765 | neogenin homolog 1 (chicken) | 114,7 | 13,7 | yes | ILMN_8819 | NM_002499.1 |
| Q92859 | nephroblastoma overexpressed gene | 226,7 | 89,3 | yes | ILMN_21404 | NM_002514.2 |
| P48745 | neural precursor cell expressed, developmentally down-regulated 8; similar to neural precursor cell expressed, developmentally down-regulated gene 8 | 5621,9 | 1325,0 | yes | ILMN_21274 | NM_006156.1 |
| Q15843 | neuroblastoma, suppression of tumorigenicity 1 | 2964,0 | 693,4 | yes | ILMN_20462 | NM_182744.1 |
| P41271 | neurofascin homolog (chicken) | 104,1 | 22,4 | yes | ILMN_138485 | NM_001005389.1 |
| O94856 | neuron derived neurotrophic factor | 1887,8 | 324,3 | yes | ILMN_29086 | NM_013349.3 |
| Q9UMY4 | neuronal growth regulator 1 | 49,9 | 2,9 | no | ILMN_20523 | NM_173808.2 |
| Q7Z3B1 | neuropilin 1 | 393,4 | 111,0 | yes | ILMN_17483 | NM_003873.4 |
| O14786 | neuropilin 2 | 60,6 | 9,1 | yes | ILMN_23292 | NM_018534.3 |
| O60462 | nicotinamide phosphoribosyltransferase | 140,8 | 35,9 | yes | ILMN_170702 | NM_005746.2 |
| P43490 | nidogen 1 | 145,3 | 22,0 | yes | ILMN_3650 | NM_002508.2 |
| P14543 | nidogen 2 (osteonidogen) | 53,6 | 3,8 | no | ILMN_28115 | NM_007361.3 |
| Q14112 | Niemann-Pick disease, type C2 | 12935,1 | 1664,2 | yes | ILMN_26452 | NM_006432.3 |
| P61916 | non-POU domain containing, octamer-binding | 1777,2 | 407,2 | yes | ILMN_172992 | NM_007363.3 |
| Q15233 | Notch homolog 2 (Drosophila) | 138,9 | 30,7 | yes | ILMN_177954 | NM_024408.2 |
| Q04721 | N-sulfoglucosamine sulfohydrolase | 1376,2 | 491,1 | yes | ILMN_7542 | NM_000199.2 |
| P51688 | nucleobindin 1 | 8842,2 | 1658,2 | yes | ILMN_29645 | NM_006184.3 |
| Q02818 | nucleobindin 2 | 161,6 | 39,7 | yes | ILMN_23510 | NM_005013.2 |
| P80303 | nudix (nucleoside diphosphate linked moiety X)-type motif 5 | 462,9 | 130,6 | yes | ILMN_1656 | NM_014142.2 |
| Q9UKU9 | OAF homolog (Drosophila) | 1812,5 | 541,0 | yes | ILMN_12751 | NM_178507.2 |
| Q86UD1 | Obg-like ATPase 1 | 48,4 | 2,0 | no | ILMN_178120 | NM_013341.3 |
| Q9NY33 | olfactomedin-like 1 | 337,0 | 204,1 | yes | ILMN_1368 | NM_198474.2 |
| Q6UWY5 | olfactomedin-like 3 | 1487,8 | 632,9 | yes | ILMN_20333 | NM_020190.2 |
| Q9NRV9 | optineurin | 1075,9 | 255,4 | yes | ILMN_23644 | NM_001008213.1 |
| Q96CV9 | osteoglycin | 69,3 | 24,5 | yes | ILMN_5435 | NM_014057.3 |
| P20774 | osteomodulin | 109,9 | 42,0 | yes | ILMN_11996 | NM_005014.1 |
| Q99983 | osteosarcoma amplified 9, endoplasmic reticulum associated protein | 125,5 | 18,9 | yes | ILMN_26656 | NM_006812.2 |
| Q13438 | OTU domain, ubiquitin aldehyde binding 1 | 72,5 | 16,2 | yes | ILMN_12271 | NM_017670.1 |
| Q96FW1 | oxidative-stress responsive 1 | 621,0 | 139,6 | yes | ILMN_13172 | NM_005109.2 |
| O95747 | pappalysin 1 | 648,8 | 248,6 | yes | ILMN_177957 | NM_002581.3 |
| Q13219 | Parkinson disease (autosomal recessive, early onset) 7 | 1836,5 | 439,6 | yes | ILMN_6178 | NM_007262.3 |
| Q99497 | pentraxin-related gene, rapidly induced by IL-1 beta | 78,7 | 18,6 | yes | ILMN_13254 | NM_002852.2 |
| P26022 | peptidase D | 1841,6 | 291,9 | yes | ILMN_22829 | NM_000285.2 |
| P12955 | peptidase inhibitor 16 | 3480,9 | 2029,3 | yes | ILMN_26089 | NM_153370.2 |
| Q6UXB8 | peptidylglycine alpha-amidating monooxygenase | 4622,5 | 351,6 | yes | ILMN_2475 | NM_138821.1 |
| P19021 | peptidylprolyl isomerase B (cyclophilin B) | 59,5 | 6,4 | yes | ILMN_25884 | NM_000942.4 |
| P23284 | periostin, osteoblast specific factor | 205,8 | 83,9 | yes | ILMN_7238 | NM_006475.1 |
| Q15063 | peroxidasin homolog (Drosophila) | 431,4 | 189,7 | yes | ILMN_165066 | NM_012293.1 |
| Q92626 | peroxiredoxin 1 | 3884,2 | 928,5 | yes | ILMN_18066 | NM_181696.1 |
| Q06830 | peroxiredoxin 2 | 1014,3 | 147,0 | yes | ILMN_8956 | NM_181738.1 |
| P32119 | peroxiredoxin 3 | 1120,3 | 241,5 | yes | ILMN_28385 | NM_006793.2 |
| P30048 | peroxiredoxin 4 | 1460,5 | 320,9 | yes | ILMN_22146 | NM_006406.1 |
| Q13162 | peroxiredoxin 5 | 3165,6 | 866,3 | yes | ILMN_19409 | NM_012094.3 |
| P30044 | peroxiredoxin 6 | 2481,6 | 677,2 | yes | ILMN_1676 | NM_004905.2 |
| P30041 | phosphatidylethanolamine binding protein 1 | 370,7 | 88,9 | yes | ILMN_16687 | NM_002567.2 |
| P30086 | phosphoglucomutase 1 | 2247,1 | 510,1 | yes | ILMN_20106 | NM_002633.2 |
| P36871 | phosphoglycerate kinase 1 | 463,1 | 183,6 | yes | ILMN_180949 | NM_000291.2 |
| P00558 | phosphoglycerate mutase 1 (brain) | 1442,4 | 215,4 | yes | ILMN_26357 | NM_002629.2 |
| P18669 | phosphoinositide-3-kinase interacting protein 1 | 934,7 | 409,2 | yes | ILMN_15026 | NM_052880.3 |
| Q96FE7 | phospholipase A2-activating protein | 62,4 | 6,0 | yes | ILMN_14096 | NM_001031689.1 |
| Q9Y287 | phospholipid transfer protein | 512,4 | 248,5 | yes | ILMN_20706 | NM_182676.1 |
| P55058 | plasma glutamate carboxypeptidase | 1155,1 | 193,6 | yes | ILMN_1988 | NM_016134.2 |
| Q9Y646 | plasminogen activator, urokinase | 203,0 | 61,4 | yes | ILMN_24167 | NM_002658.2 |
| P00749 | plasminogen activator, urokinase receptor | 337,9 | 112,4 | yes | ILMN_24538 | NM_001005376.1 |
| Q03405 | platelet derived growth factor D | 309,2 | 134,7 | yes | ILMN_28048 | NM_025208.4 |
| Q9H173 | platelet-derived growth factor receptor, alpha polypeptide | 1086,2 | 612,8 | yes | ILMN_165232 | NM_006206.3 |
| P16234 | platelet-derived growth factor receptor, beta polypeptide | 4837,4 | 1579,8 | yes | ILMN_25767 | NM_002609.3 |
| P09619 | platelet-derived growth factor receptor-like | 1339,3 | 272,5 | yes | ILMN_178894 | NM_006207.1 |
| Q15198 | plexin B2 | 1006,4 | 190,9 | yes | ILMN_308861 | NM_012401.2 |
| O15031 | plexin domain containing 2 | 415,9 | 203,4 | yes | ILMN_29366 | NM_032812.7 |
| Q6UX71 | podocan | 1795,7 | 511,2 | yes | ILMN_28316 | NM_153703.3 |
| Q7Z5L7 | poly(rC) binding protein 1 | 3120,4 | 646,1 | yes | ILMN_7900 | NM_006196.2 |
| Q15365 | polymerase I and transcript release factor | 3162,0 | 1334,8 | yes | ILMN_22301 | NM_012232.3 |
| Q6NZI2 | polypyrimidine tract binding protein 1 | 2645,1 | 566,9 | yes | ILMN_20993 | NM_002819.3 |
| P26599 | pregnancy specific beta-1-glycoprotein 3 | 226,9 | 184,1 | yes | ILMN_14040 | NM_021016.3 |
| Q16557 | pregnancy specific beta-1-glycoprotein 5 | 261,6 | 226,3 | yes | ILMN_21422 | NM_002781.2 |
| Q15238 | pregnancy specific beta-1-glycoprotein 6 | 54,3 | 12,1 | no | ILMN_29461 | NM_002782.3 |
| Q00889 | pregnancy specific beta-1-glycoprotein 9 | 78,3 | 33,4 | yes | ILMN_17742 | NM_002784.2 |
| Q00887 | pregnancy-zone protein | 47,7 | 3,6 | no | ILMN_16323 | NM_002864.1 |
| P20742 | prion protein | 1351,1 | 448,5 | yes | ILMN_11177 | NM_183079.2 |
| P04156 | procollagen C-endopeptidase enhancer | 3346,7 | 973,6 | yes | ILMN_13969 | NM_002593.2 |
| Q15113 | procollagen C-endopeptidase enhancer 2 | 263,3 | 195,8 | yes | ILMN_14782 | NM_013363.2 |
| Q9UL46 | procollagen-lysine 1, 2-oxoglutarate 5-dioxygenase 1 | 2410,4 | 486,7 | yes | ILMN_20559 | NM_000302.2 |
| Q02809 | procollagen-lysine, 2-oxoglutarate 5-dioxygenase 2 | 1497,6 | 642,1 | yes | ILMN_25982 | NM_000935.2 |
| O00469 | procollagen-lysine, 2-oxoglutarate 5-dioxygenase 3 | 1831,1 | 492,1 | yes | ILMN_9049 | NM_001084.4 |
| O60568 | programmed cell death 5 | 478,0 | 108,3 | yes | ILMN_8109 | NM_004708.2 |
| O14737 | proline/arginine-rich end leucine-rich repeat protein | 79,4 | 36,5 | yes | ILMN_18101 | NM_201348.1 |
| P51888 | prolyl 4-hydroxylase, beta polypeptide | 4325,8 | 677,7 | yes | ILMN_27535 | NM_000918.3 |
| P07237 | prolylcarboxypeptidase (angiotensinase C) | 2411,5 | 417,6 | yes | ILMN_174053 | NM_199418.2 |
| P42785 | proprotein convertase subtilisin/kexin type 5 | 229,0 | 104,2 | yes | ILMN_6597 | NM_006200.2 |
| Q92824 | proprotein convertase subtilisin/kexin type 9 | 151,5 | 50,3 | yes | ILMN_15492 | NM_174936.2 |
| Q8NBP7 | prosaposin | 7588,9 | 836,4 | yes | ILMN_3081 | NM_002778.2 |
| P07602 | prostaglandin D2 synthase, hematopoietic; prostaglandin D2 synthase 21kDa (brain) | 1080,5 | 1189,2 | yes | ILMN_19248 | NM_000954.5 |
| P41222 | prostaglandin reductase 1 | 682,6 | 149,0 | yes | ILMN_8394 | NM_012212.2 |
| Q14914 | protease, serine, 23 | 387,5 | 41,2 | yes | ILMN_22272 | NM_007173.4 |
| O95084 | proteasome 26S subunit, non-ATPase, 2 | 561,6 | 120,9 | yes | ILMN_13906 | NM_002808.3 |
| Q13200 | proteasome (prosome, macropain) activator subunit 1 (PA28 alpha) | 1298,0 | 476,6 | yes | ILMN_16076 | NM_006263.2 |
| Q06323 | proteasome (prosome, macropain) activator subunit 2 (PA28 beta) | 221,6 | 53,7 | yes | ILMN_19572 | NM_002818.2 |
| Q9ULA0 | protein C receptor, endothelial (EPCR) | 133,5 | 22,9 | yes | ILMN_21711 | NM_006404.3 |
| Q9UNW1 | protein disulfide isomerase family A, member 3 | 47,2 | 4,4 | no | ILMN_177518 | NM_005313.4 |
| P30101 | protein disulfide isomerase family A, member 4 | 92,3 | 13,1 | yes | ILMN_4575 | NM_004911.3 |
| P13667 | protein disulfide isomerase family A, member 6 | 406,5 | 104,9 | yes | ILMN_9638 | NM_005742.2 |
| Q15084 | protein kinase C substrate 80K-H | 659,0 | 67,6 | yes | ILMN_27924 | NM_001001329.1 |
| P14314 | protein S (alpha) | 647,5 | 148,2 | yes | ILMN_3398 | NM_000313.1 |
| P07225 | protein tyrosine phosphatase, receptor type, G | 58,9 | 7,3 | yes | ILMN_17149 | NM_002841.2 |
| P23470 | protein tyrosine phosphatase, receptor type, S | 53,2 | 4,7 | no | ILMN_1165 | NM_130855.2 |
| Q13332 | proteoglycan 4 | 69,1 | 12,9 | yes | ILMN_1596 | NM_001897.4 |
| Q92954 | PTK7 protein tyrosine kinase 7 | 344,5 | 42,3 | yes | ILMN_898 | NM_152882.2 |
| Q13308 | pyridoxal (pyridoxine, vitamin B6) kinase | 2649,1 | 783,4 | yes | ILMN_24569 | NM_003681.4 |
| O00764 | quiescin Q6 sulfhydryl oxidase 1 | 314,6 | 127,7 | yes | ILMN_17337 | NM_001004128.2 |
| O00391 | RAB2A, member RAS oncogene family | 131,8 | 13,5 | yes | ILMN_18037 | NM_002865.1 |
| P61019 | RAB7A, member RAS oncogene family | 3178,8 | 647,8 | yes | ILMN_30038 | NM_004637.5 |
| P51149 | ras homolog gene family, member A | 1718,5 | 354,8 | yes | ILMN_179821 | NM_001664.2 |
| P61586 | Ras suppressor protein 1 | 287,8 | 38,7 | yes | ILMN_21628 | NM_012425.3 |
| Q15404 | related RAS viral (r-ras) oncogene homolog 2; similar to related RAS viral (r-ras) oncogene homolog 2 | 237,7 | 31,0 | yes | ILMN_165732 | NM_012250.3 |
| P62070 | reticulocalbin 1, EF-hand calcium binding domain | 3269,2 | 913,0 | yes | ILMN_8159 | NM_002901.1 |
| Q15293 | reticulocalbin 2, EF-hand calcium binding domain | 1103,2 | 177,4 | yes | ILMN_8519 | NM_002902.1 |
| Q14257 | reticulocalbin 3, EF-hand calcium binding domain | 539,5 | 136,0 | yes | ILMN_7768 | NM_020650.2 |
| Q96D15 | reticulon 4 | 4932,0 | 961,6 | yes | ILMN_19851 | NM_007008.2 |
| Q9NR99 | retinoic acid receptor responder (tazarotene induced) 2 | 3034,2 | 1532,0 | yes | ILMN_15763 | NM_002889.2 |
| Q99969 | reversion-inducing-cysteine-rich protein with kazal motifs | 264,6 | 75,1 | yes | ILMN_23308 | NM_021111.1 |
| O95980 | RGM domain family, member B | 97,3 | 36,0 | yes | ILMN_501 | NM_001012761.1 |
| Q6NW40 | ribonuclease T2 | 1324,7 | 278,6 | yes | ILMN_412 | NM_003730.3 |
| O00584 | ribosomal protein, large, P2 pseudogene 3; ribosomal protein, large, P2 | 10086,1 | 1831,5 | yes | ILMN_138635 | NM_001004.2 |
| P05387 | RNA binding motif protein 12; copine I | 517,8 | 209,5 | yes | ILMN_6266 | NM_152931.1 |
| Q99829 | roundabout, axon guidance receptor, homolog 1 (Drosophila); similar to roundabout 1 isoform b | 47,5 | 3,6 | no | ILMN_21474 | NM_133631.1 |
| Q9Y6N7 | secreted frizzled-related protein 2 | 1127,5 | 906,8 | yes | ILMN_24078 | NM_003013.2 |
| Q96HF1 | secreted frizzled-related protein 4 | 1618,1 | 1363,8 | yes | ILMN_13024 | NM_003014.2 |
| Q6FHJ7 | secreted phosphoprotein 1 | 281,2 | 219,4 | yes | ILMN_9394 | NM_000582.2 |
| P10451 | secreted protein, acidic, cysteine-rich (osteonectin) | 3467,4 | 1376,8 | yes | ILMN_1780 | NM_003118.2 |
| P09486 | secretogranin II (chromogranin C) | 197,0 | 114,7 | yes | ILMN_17827 | NM_003469.3 |
| P13521 | selenoprotein M | 1278,9 | 215,7 | yes | ILMN_1005 | NM_080430.2 |
| Q8WWX9 | semaphorin 7A, GPI membrane anchor (John Milton Hagen blood group) | 92,2 | 38,4 | yes | ILMN_17564 | NM_003612.2 |
| O75326 | septin 2 | 5107,8 | 674,4 | yes | ILMN_4091 | NM_004404.3 |
| Q15019 | septin 9 | 4383,6 | 1123,0 | yes | ILMN_14584 | NM_006640.3 |
| Q9UHI8 | serglycin | 214,1 | 62,6 | yes | ILMN_2142 | NM_002727.2 |
| P10124 | serpin peptidase inhibitor, clade C (antithrombin), member 1 | 44,2 | 2,7 | no | ILMN_18442 | NM_000488.2 |
| P01008 | serpin peptidase inhibitor, clade E (nexin, plasminogen activator inhibitor type 1), member 1 | 1403,6 | 1115,0 | yes | ILMN_6244 | NM_000602.1 |
| P05121 | serpin peptidase inhibitor, clade E (nexin, plasminogen activator inhibitor type 1), member 2 | 16851,9 | 7785,4 | yes | ILMN_1946 | NM_006216.2 |
| P07093 | serpin peptidase inhibitor, clade F (alpha-2 antiplasmin, pigment epithelium derived factor), member 1 | 487,4 | 173,5 | yes | ILMN_19076 | NM_002615.4 |
| P36955 | serpin peptidase inhibitor, clade G (C1 inhibitor), member 1 | 512,4 | 216,7 | yes | ILMN_15074 | NM_001032295.1 |
| P05155 | serpin peptidase inhibitor, clade H (heat shock protein 47), member 1, (collagen binding protein 1) | 3289,4 | 969,6 | yes | ILMN_28241 | NM_001235.2 |
| P50454 | SET nuclear oncogene; similar to SET translocation | 296,4 | 112,0 | yes | ILMN_180677 | NM_003011.2 |
| Q01105 | sialic acid acetylesterase | 51,5 | 4,7 | no | ILMN_10502 | NM_170601.3 |
| Q9HB07 | sialidase 1 (lysosomal sialidase) | 646,7 | 115,0 | yes | ILMN_23298 | NM_000434.2 |
| Q99519 | signal peptide, CUB domain, EGF-like 3 | 48,6 | 3,4 | no | ILMN_844 | NM_152753.2 |
| Q8IX30 | signal transducer and activator of transcription 1, 91kDa | 1809,1 | 361,3 | yes | ILMN_163602 | NM_007315.2 |
| P42224 | SIL1 homolog, endoplasmic reticulum chaperone (S. cerevisiae) | 735,7 | 145,6 | yes | ILMN_10838 | NM_001037633.1 |
| Q9H2G2 | similar to Complement C3 precursor; complement component 3; hypothetical protein LOC100133511 | 105,8 | 76,5 | yes | ILMN_5682 | NM_000064.1 |
| P01024 | similar to growth arrest-specific 6; growth arrest-specific 6 | 6820,0 | 2681,6 | yes | ILMN_10723 | NM_000820.1 |
| Q14393 | similar to TRIMCyp; peptidylprolyl isomerase A (cyclophilin A); peptidylprolyl isomerase A (cyclophilin A)-like 3 | 71,4 | 7,1 | yes | ILMN_25214 | NM_021130.3 |
| P62937 | slit homolog 3 (Drosophila) | 1718,5 | 325,1 | yes | ILMN_18656 | NM_003062.1 |
| O75094 | sorting nexin 12 | 71,0 | 6,8 | yes | ILMN_14112 | NM_013346.2 |
| Q9UNN8 | sorting nexin 3 | 3564,2 | 555,2 | yes | ILMN_3389 | NM_003795.3 |
| O60493 | sorting nexin 9 | 46,1 | 1,7 | no | ILMN_23646 | NM_016224.3 |
| Q9Y5Z4 | sparc/osteonectin, cwcv and kazal-like domains proteoglycan (testican) 1 | 4544,4 | 1540,2 | yes | ILMN_25886 | NM_004598.3 |
| Q08629 | sphingomyelin phosphodiesterase 1, acid lysosomal | 173,2 | 43,6 | yes | ILMN_10742 | NM_000543.3 |
| P17405 | sphingomyelin phosphodiesterase, acid-like 3A | 812,4 | 296,8 | yes | ILMN_16204 | NM_006714.2 |
| Q92484 | spondin 1, extracellular matrix protein | 735,1 | 534,9 | yes | ILMN_7397 | NM_006108.2 |
| Q9HCU0 | spondin 2, extracellular matrix protein | 341,0 | 95,7 | yes | ILMN_17453 | NM_012445.1 |
| Q9BUD6 | stanniocalcin 1 | 58,3 | 6,5 | yes | ILMN_16225 | NM_003155.2 |
| P52823 | stanniocalcin 2 | 988,9 | 358,0 | yes | ILMN_28725 | NM_003714.2 |
| O76061 | stathmin 1 | 121,3 | 31,9 | yes | ILMN_12586 | NM_203401.1 |
| P16949 | staufen, RNA binding protein, homolog 1 (Drosophila) | 2967,2 | 302,1 | yes | ILMN_23551 | NM_017454.2 |
| O95793 | stomatin | 689,0 | 218,0 | yes | ILMN_183628 | NM_004099.4 |
| P27105 | stress-induced-phosphoprotein 1 | 551,0 | 61,4 | yes | ILMN_28761 | NM_006819.1 |
| P31948 | stromal cell derived factor 4 | 1639,1 | 264,0 | yes | ILMN_25183 | NM_016547.1 |
| Q9BRK5 | sulfatase modifying factor 1 | 635,5 | 133,0 | yes | ILMN_162213 | NM_182760.2 |
| Q8NBK3 | sulfatase modifying factor 2 | 3148,2 | 556,1 | yes | ILMN_164657 | NM_001042470.1 |
| Q8NBJ7 | superoxide dismutase 3, extracellular | 129,7 | 67,5 | yes | ILMN_184013 | NM_003102.2 |
| P08294 | sushi, nidogen and EGF-like domains 1 | 46,0 | 3,0 | no | ILMN_172231 | NM_001080437.1 |
| Q8TER0 | sushi, von Willebrand factor type A, EGF and pentraxin domain containing 1 | 2228,4 | 1083,0 | yes | ILMN_179703 | NM_153366.2 |
| Q4LDE5 | sushi-repeat-containing protein, X-linked | 2187,2 | 466,4 | yes | ILMN_26705 | NM_006307.3 |
| P78539 | sushi-repeat-containing protein, X-linked 2 | 228,8 | 58,5 | yes | ILMN_23854 | NM_014467.2 |
| O60687 | syntaxin 7 | 165,7 | 24,7 | yes | ILMN_23974 | NM_003569.1 |
| O15400 | talin 1 | 522,0 | 164,6 | yes | ILMN_18029 | NM_006289.2 |
| Q9Y4K0 | tenascin C | 941,4 | 364,1 | yes | ILMN_14948 | NM_002160.2 |
| P24821 | tenascin XB; tenascin XA pseudogene | 59,6 | 18,6 | yes | ILMN_23726 | NR_001284.1 |
| P22105 | thioredoxin domain containing 12 (endoplasmic reticulum) | 1051,1 | 262,4 | yes | ILMN_22592 | NM_015913.2 |
| O95881 | thioredoxin domain containing 5 (endoplasmic reticulum); muted homolog (mouse) | 2533,9 | 744,1 | yes | ILMN_24968 | NM_030810.2 |
| Q8NBS9 | THO complex 4 | 435,7 | 64,5 | yes | ILMN_166855 | XM_001134346.1 |
| Q86V81 | thrombospondin 1 | 7060,3 | 6165,8 | yes | ILMN_182705 | NM_003246.2 |
| P07996 | thrombospondin 2 | 4770,2 | 2547,2 | yes | ILMN_16098 | NM_003247.2 |
| P35442 | thrombospondin 3 | 89,6 | 20,6 | yes | ILMN_10000 | NM_007112.3 |
| P49746 | TIMP metallopeptidase inhibitor 1 | 21002,9 | 3899,4 | yes | ILMN_3162 | NM_003254.2 |
| P01033 | TIMP metallopeptidase inhibitor 2 | 13078,6 | 3125,6 | yes | ILMN_1046 | NM_003255.4 |
| P16035 | tissue factor pathway inhibitor (lipoprotein-associated coagulation inhibitor) | 309,1 | 71,6 | yes | ILMN_17834 | NM_006287.4 |
| P10646 | tissue factor pathway inhibitor 2 | 50,0 | 4,0 | no | ILMN_17570 | NM_006528.2 |
| P48307 | transcobalamin II; macrocytic anemia | 149,2 | 47,5 | yes | ILMN_6136 | NM_000355.2 |
| P20062 | transferrin | 55,3 | 5,7 | no | ILMN_25028 | NM_001063.2 |
| P02787 | transforming growth factor, beta receptor III | 823,7 | 567,9 | yes | ILMN_22620 | NM_003243.2 |
| Q03167 | transforming growth factor, beta-induced, 68kDa | 5457,6 | 4812,8 | yes | ILMN_24587 | NM_000358.1 |
| Q15582 | transketolase | 4368,1 | 1143,3 | yes | ILMN_2726 | NM_001064.1 |
| P29401 | transthyretin | 58,2 | 3,1 | yes | ILMN_2710 | NM_000371.1 |
| P02766 | tripeptidyl peptidase I | 1335,7 | 383,1 | yes | ILMN_19883 | NM_000391.3 |
| O14773 | tropomyosin 1 (alpha) | 4095,6 | 2774,7 | yes | ILMN_8136 | NM_001018004.1 |
| P09493 | tropomyosin 3 | 277,0 | 84,8 | yes | ILMN_5807 | NM_152263.2 |
| P06753 | tropomyosin 4 | 362,4 | 132,6 | yes | ILMN_9334 | NM_003290.1 |
| P67936 | tubulin folding cofactor A | 3716,6 | 598,1 | yes | ILMN_8008 | NM_004607.2 |
| O75347 | tubulointerstitial nephritis antigen-like 1 | 47,9 | 4,6 | no | ILMN_28140 | NM_022164.1 |
| Q9GZP0 | tumor necrosis factor receptor superfamily, member 11b | 164,3 | 76,1 | yes | ILMN_6495 | NM_002546.3 |
| O00300 | tumor necrosis factor receptor superfamily, member 12A | 500,4 | 328,6 | yes | ILMN_11654 | NM_016639.1 |
| Q9NQ30 | tumor necrosis factor, alpha-induced protein 6 | 418,8 | 75,8 | yes | ILMN_11686 | NM_007115.2 |
| P98066 | twinfilin, actin-binding protein, homolog 2 (Drosophila) | 758,1 | 179,2 | yes | ILMN_717 | NM_007284.3 |
| Q6IBS0 | ubiquitin-conjugating enzyme E2N (UBC13 homolog, yeast) | 346,7 | 79,3 | yes | ILMN_173917 | NM_003348.3 |
| P61088 | ubiquitin-like modifier activating enzyme 1 | 1003,4 | 161,6 | yes | ILMN_10467 | NM_003334.2 |
| P22314 | UDP-glucose ceramide glucosyltransferase-like 1 | 151,9 | 33,8 | yes | ILMN_17794 | NM_020120.2 |
| Q9NZ08 | UDP-glucose dehydrogenase | 435,7 | 97,0 | yes | ILMN_3906 | NM_003359.2 |
| O60701 | UDP-N-acetyl-alpha-D-galactosamine:polypeptide N-acetylgalactosaminyltransferase 10 (GalNAc-T10) | 229,7 | 89,3 | yes | ILMN_8911 | NM_017540.3 |
| Q86SR1 | UDP-N-acetyl-alpha-D-galactosamine:polypeptide N-acetylgalactosaminyltransferase 2 (GalNAc-T2) | 58,0 | 5,5 | yes | ILMN_2160 | NM_004481.2 |
| Q10471 | UDP-N-acetyl-alpha-D-galactosamine:polypeptide N-acetylgalactosaminyltransferase 5 (GalNAc-T5) | 63,0 | 16,4 | yes | ILMN_18326 | NM_014568.1 |
| Q7Z7M9 | VAMP (vesicle-associated membrane protein)-associated protein A, 33kDa | 88,8 | 10,7 | yes | ILMN_27400 | NM_003574.4 |
| Q9P121 | vascular endothelial growth factor C | 455,5 | 109,8 | yes | ILMN_161973 | NM_005429.2 |
| P49767 | vasodilator-stimulated phosphoprotein | 58,0 | 10,1 | yes | ILMN_28263 | NM_003370.3 |
| P50552 | vasorin | 3620,4 | 1097,2 | yes | ILMN_31069 | NM_138440.2 |
| Q6EMK4 | v-crk sarcoma virus CT10 oncogene homolog (avian) | 626,9 | 182,5 | yes | ILMN_25875 | NM_016823.2 |
| P46108 | versican | 1097,2 | 472,5 | yes | ILMN_25778 | NM_004385.2 |
| P13611 | vesicle amine transport protein 1 homolog (T. californica) | 823,5 | 257,1 | yes | ILMN_26285 | NM_006373.3 |
| Q99536 | vitronectin | 47,7 | 5,5 | no | ILMN_38193 | NM_000638.3 |
| P04004 | WD repeat domain 1 | 3236,5 | 1111,4 | yes | ILMN_14401 | NM_005112.4 |
| O75083 | WNT1 inducible signaling pathway protein 2 | 3100,6 | 1232,0 | yes | ILMN_7098 | NM_003881.2 |
| O76076 | X-ray repair complementing defective repair in Chinese hamster cells 5 (double-strand-break rejoining) | 162,2 | 48,9 | yes | ILMN_14826 | NM_021141.2 |
| P13010 | X-ray repair complementing defective repair in Chinese hamster cells 6; similar to ATP-dependent DNA helicase II, 70 kDa subunit | 324,1 | 58,0 | yes | ILMN_13892 | NM_001469.3 |
